# Boron-Functionalised *exo*-Norbornene Monomers for ROMP: Toward Coordination-Active Poly(norbornene-B-arylene-dioxaborepine)s

**DOI:** 10.3390/molecules31183151

**Published:** 2026-09-08

**Authors:** Jerzy Garbarek, Mariusz Majchrzak, Maciej Kubicki

**Affiliations:** 1 Department of Organometallic Chemistry, Faculty of Chemistry, Adam Mickiewicz University in Poznań, 8 Uniwersytetu Poznańskiego Str., 61-614 Poznań, Poland; jerzy.garbarek@amu.edu.pl; 2 Crystallography Department, Faculty of Chemistry, Adam Mickiewicz University in Poznań, 8 Uniwersytetu Poznańskiego Str., 61-614 Poznań, Poland; maciej.kubicki@amu.edu.pl

**Keywords:** boron, boronic ester, Grubbs ruthenium catalysts, polymer, ring-opening polymerisation, ROMP

## Abstract

A series of boron-functionalised *exo*-norbornene monomers bearing boronic ester groups were synthesised via a condensation reaction and subsequently polymerised through ring-opening metathesis polymerisation (ROMP) to afford well-defined poly(norbornene-B-arylene-dioxaborepine)s. The incorporation of Lewis acidic boron centres into the norbornene-derived polymer backbone enables access to coordination-active macromolecular systems with tuneable interactions towards nucleophilic species. The resulting polymers demonstrate enhanced structural definition and stability, thereby confirming the robustness of the synthetic approach and the successful incorporation of boron functionalities into ROMP-derived architectures. This work establishes a general and efficient strategy for the preparation of boron-containing ROMP polymers, providing a versatile platform for the development of coordination-responsive polymeric materials.

## 1. Introduction

In recent years, there has been increased interest in ring-opening metathesis polymerisation as an exquisite tool for acquiring linear-based polymers from strained, electron-rich cyclic unsaturated molecules [1]. This process allows for facile synthesis of polymeric or copolymeric linear chains bearing diverse features with different side-chain-substituted functional groups, for purposes such as conducting polymers [2,3,4,5,6,7], stereoregular fluoropolymers [8,9], electroluminescent materials [10,11,12,13] or graft copolymers [14,15,16]. With the discovery of carbene-type ruthenium catalysts (Grubbs catalysts), which, to some extent, are tolerant to polar groups and water, it has become more prevalent to use these initiators in ROMP [6,7,12,13,15,16]. What is more, the reaction can be easily controlled, resulting in low-polydispersity index of forming polymeric chains [17,18,19,20]. Boronic esters are an interesting group of compounds obtained by reaction between diol moieties and either boronic acids in the condensation process or corresponding dibromides in the process of alcoholysis [21,22]. These two methods exhibited the highest product yields in comparison to others. In the past, these compounds had their application in the synthesis of optically active aldehydes and alcohols in high optical purity [23] or as protective groups in carbohydrate chemistry [24]. The presence of alkoxy groups and relatively stable boron–oxygen bonds allows the preparation of durable chemical compounds linked with different functional groups. To this day, one of the most widespread applications of boronic ester-containing compounds is the Suzuki–Miyaura cross-coupling reaction, which enables the formation of new carbon–carbon bonds between an organohalide and a boronic ester. Such boronic esters are often prepared via Miyaura borylation using bis(pinacolato)diboron (B_2_pin_2_) [25].

In addition, boron atoms present in structure of both monomers and polymer backbones may prove to be of interest, e.g., by binding with nucleophilic compounds such as amines [26,27,28,29,30] or phosphines [31] thus forming B-N or B-P coordination bonds. To date, the fact that boro-organic compounds exhibit electrophilic properties has been used in medicine by binding dopamine [32,33,34] and saccharide [35,36,37,38,39,40] molecules, in the reduction of amides, aldehydes and ketones [41,42,43], in improving conductivity in polymers (polianiline) [44] and reducing steric hindrance of others (PBZT) [45], as sensors [46,47] or in BNCT therapy [48,49]. However, to the best of our knowledge, there is very little information on norbornene-based polymeric materials containing boron-based functional groups in various structural configurations, prepared via ROMP polymerisation [50,51,52,53,54]. Our previous studies regarding the norbornene-based polymers bearing silicon [55], organo-silicon-styryl-based boronic esters [56] and ferrocene-boronic derived material [57] confirm the possibility of further development of the subject presented herein. With regard to the organometallic polymerisation of norbornene-type structures it is worth mentioning the importance of *endo*-/*exo*-isomers of substituted norbornene. In studies on the reactivity of dicyclopentadiene, the *endo*-isomer was found to be less than a magnitude reactive than its *exo*-form. This can be attributed to steric interactions between the endo substituent of the approaching monomer and the substituents of the cyclopentane ring, to which the propagating centre is bound. A second reason for this phenomenon is the formation of an intramolecular complex between the ruthenium centre (in case of ROMP) and the adjacent vinylene double bond [58].

What is more, Lewis acidic boron centres have emerged as versatile functional motifs in polymeric materials owing to their ability to reversibly coordinate nucleophilic species through Lewis acid-base interactions. Such interactions provide an effective strategy for tuning the electronic structure and physicochemical properties of boron-containing materials. In particular, triarylborane-based polymers and porous polymer networks have demonstrated selective recognition and binding of fluoride and other nucleophilic anions, resulting in substantial modulation of charge-transfer processes and photophysical properties [59,60,61,62]. Coordination of nucleophiles to electron-deficient boron centres can induce pronounced changes in absorption and fluorescence, enabling the development of colorimetric and fluorometric sensors, molecular recognition systems, and stimuli-responsive luminescent materials with tuneable optical outputs [59,60,61,62,63].

Beyond sensing applications, reversible boron-donor coordination offers unique opportunities for the design of adaptive and dynamic polymeric materials. Dynamic B-N interactions have been shown to facilitate bond exchange processes, regulate network rearrangement, and promote self-healing behaviour through reversible association and dissociation mechanisms [64,65,66]. Such interactions can simultaneously enhance mechanical robustness and enable autonomous recovery, thereby providing access to polymeric systems with tuneable mechanical and functional properties [66]. Consequently, coordination-active boron centres represent far more than simple recognition sites and constitute valuable structural elements for the development of responsive optical materials, self-healing polymers, molecular recognition platforms, and other advanced functional systems based on controllable Lewis acid-base interactions [59,60,61,62,63,64,65,66].

**Scheme 1:**
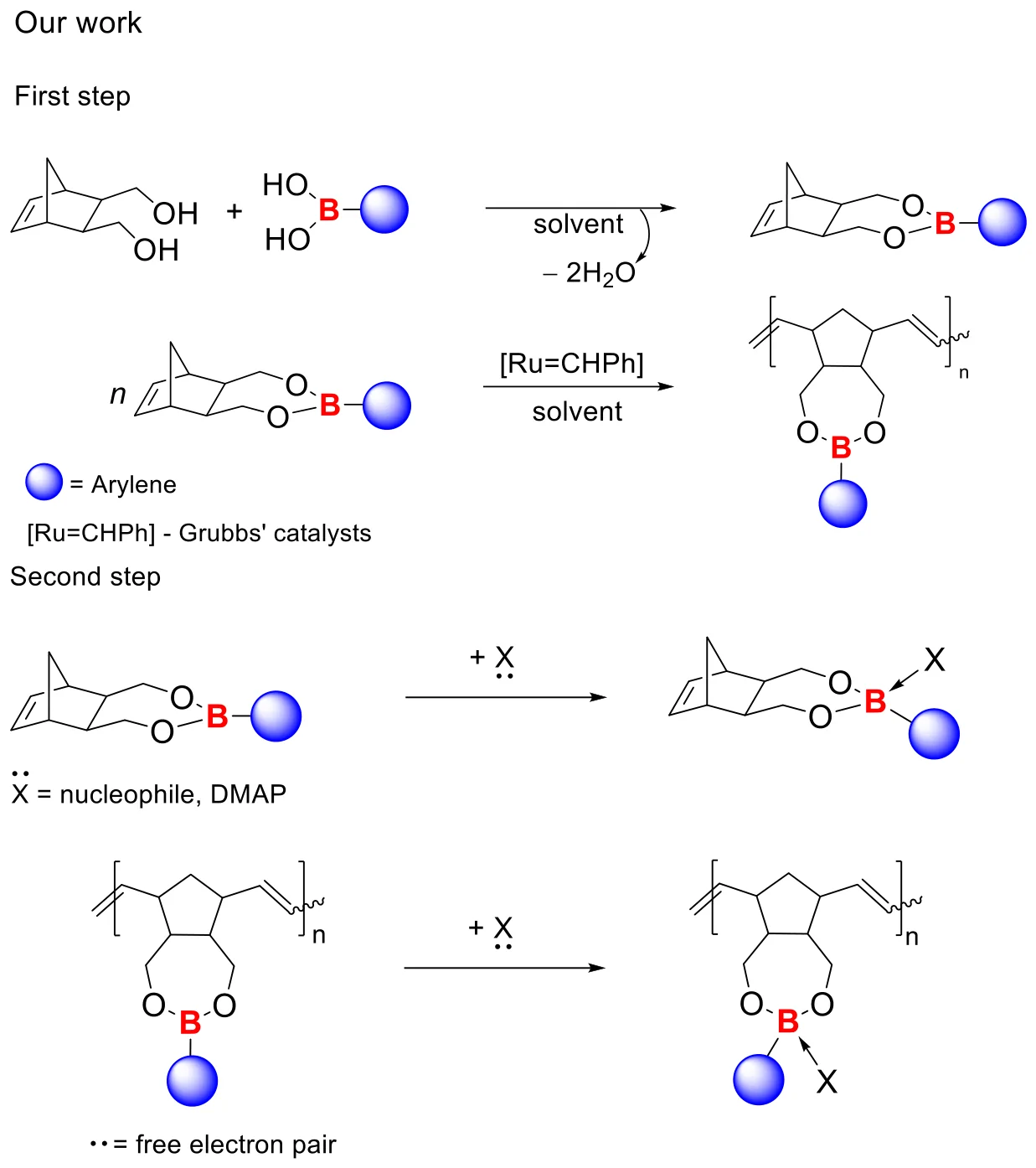
General overview of research.

This paper presents an efficient method for the synthesis of new 5-norbornene-2-*exo*-3-*exo*-dimethyl-[(dioxaborepine)] derivatives by condensation reaction followed by ring-opening metathesis polymerisation, resulting in polymer chains with high yield of products, see Scheme 1. An example of coordination process will also be presented as an application for binding nucleophilic type moieties.

An example of the coordination process will also be presented as an application for binding nucleophilic molecules. This process may demonstrate the effective removal of potentially hazardous substances from the environment.

## 2. Results and Discussion

### 2.1. Preliminary Studies

**Scheme 2:**
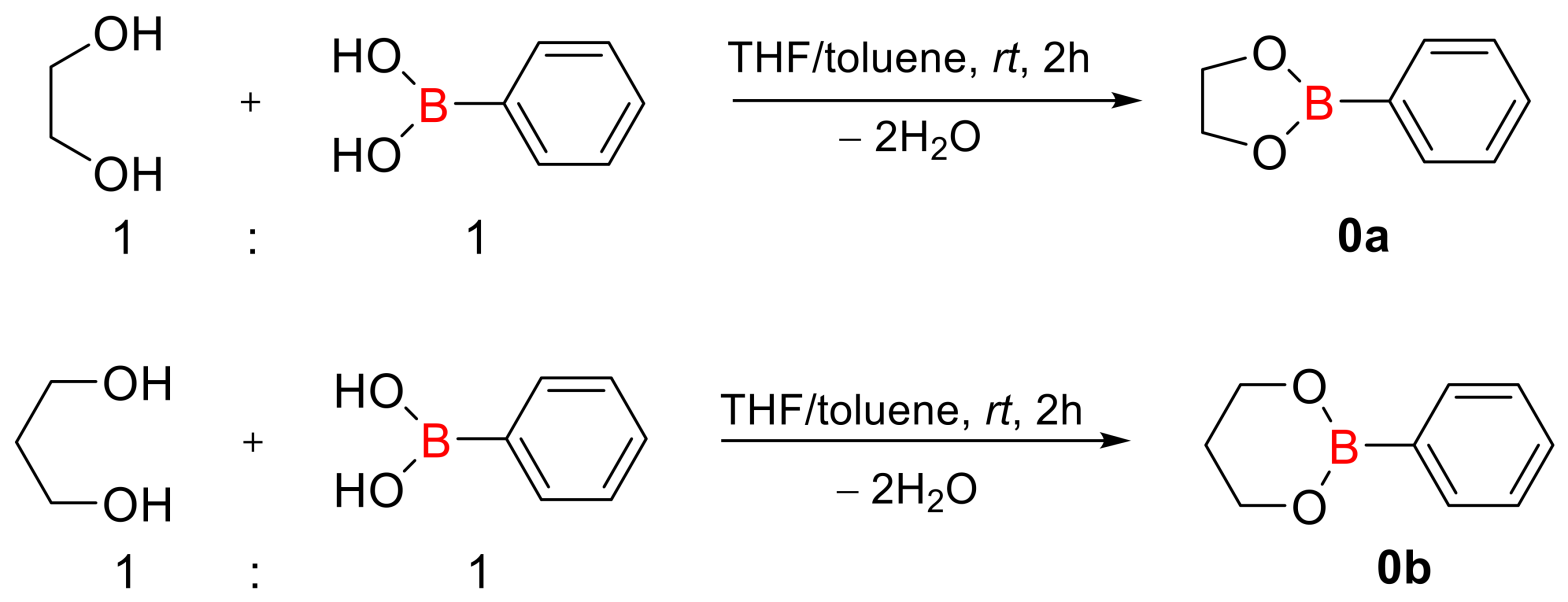
Preliminary studies: the condensation reaction of phenylboronic acid with diols.

The main objective of the preliminary studies was to develop an effective and selective synthetic pathway that would be feasible for all of the boronic acids combined in this work. In order to achieve that goal, ethylene glycol and 1,3-propanediol have been separately mixed with phenylboronic acid in a weakly polar solvent—toluene and highly polar reaction medium—THF for 2 h at room temperature, see Scheme 2. The resulting boronic esters 0a and 0b are commercially available substances; however, it was important to oversee the progress of the reaction and especially the time required for full condensation. After approximately 2 h, both GC-MS and infrared spectroscopy indicated absence of ethylene glycol or 1,3-propanediol (conversion over > 99%) and the presence of the desired product. Moreover, the phenylboronic acid was also completely consumed (over > 99%). The IR monitored the disappearance of the broad C-OH bands of both substrates in the range of 3000 cm^−1^ and 3650 cm^−1^ and formation of an intense peak between 1100 cm^−1^ and 1300 cm^−1^ that corresponds to the C-O stretching vibration that can be attributed to the -H_2_C-O-B-ether part. The secondary purpose of this research was to observe if and to what extent cyclic boroxines could be formed as by-products via GC-MS. In this case, no formation of any by-products was visible (other than H_2_O). The use of both analytical methods provided information on the composition of the reaction mixture and allowed optimisation of the process with full control over the conditions. The tests in THF and toluene showed the latter to be better in terms of isolation of the final product, which has been analysed with standard spectroscopic methods (FT-IR, ^1^H NMR, ^13^C NMR) along with GC-MS. While infrared spectroscopy, mass spectrometry and proton nuclear magnetic resonance provided confirmation of the synthesis of desired product, the ^13^C NMR lacked one peak responsible for the B-C bond. Investigation on the subject provided information that this connection is not always visible via carbon nuclear magnetic resonance, which has been acknowledged by the literature references [67,68,69,70]. This phenomenon was observed further on in the case of the monomers and polymers (for the results, see the Supplementary Materials).

The optimisation tests of the reaction conditions described above indicated that toluene was the optimal environment. In this solvent, following complete conversion of the reactants, a two-phase system was observed, in which the presence of water did not cause the formation of a byproduct. Moreover, this elementary synthetic protocol does not necessitate the utilisation of a Dean–Stark trap. In the case of THF, the drying step with MgSO_4_ necessitated an extended drying time and additional rapid filtration through SiO_2_.

### 2.2. Synthesis of Monomers

Condensation reaction proved to be a very easy and efficient way to synthesise ROMP-prone monomers (M1–M7) (Table 1). In each case, optimisation of reaction conditions was performed [71]. The conditions vary in temperature and time, depending on solubility of specific boronic acid. Only in the case of M1 was full dissolution of boronic acid observed at room temperature.

**Figure 1:**
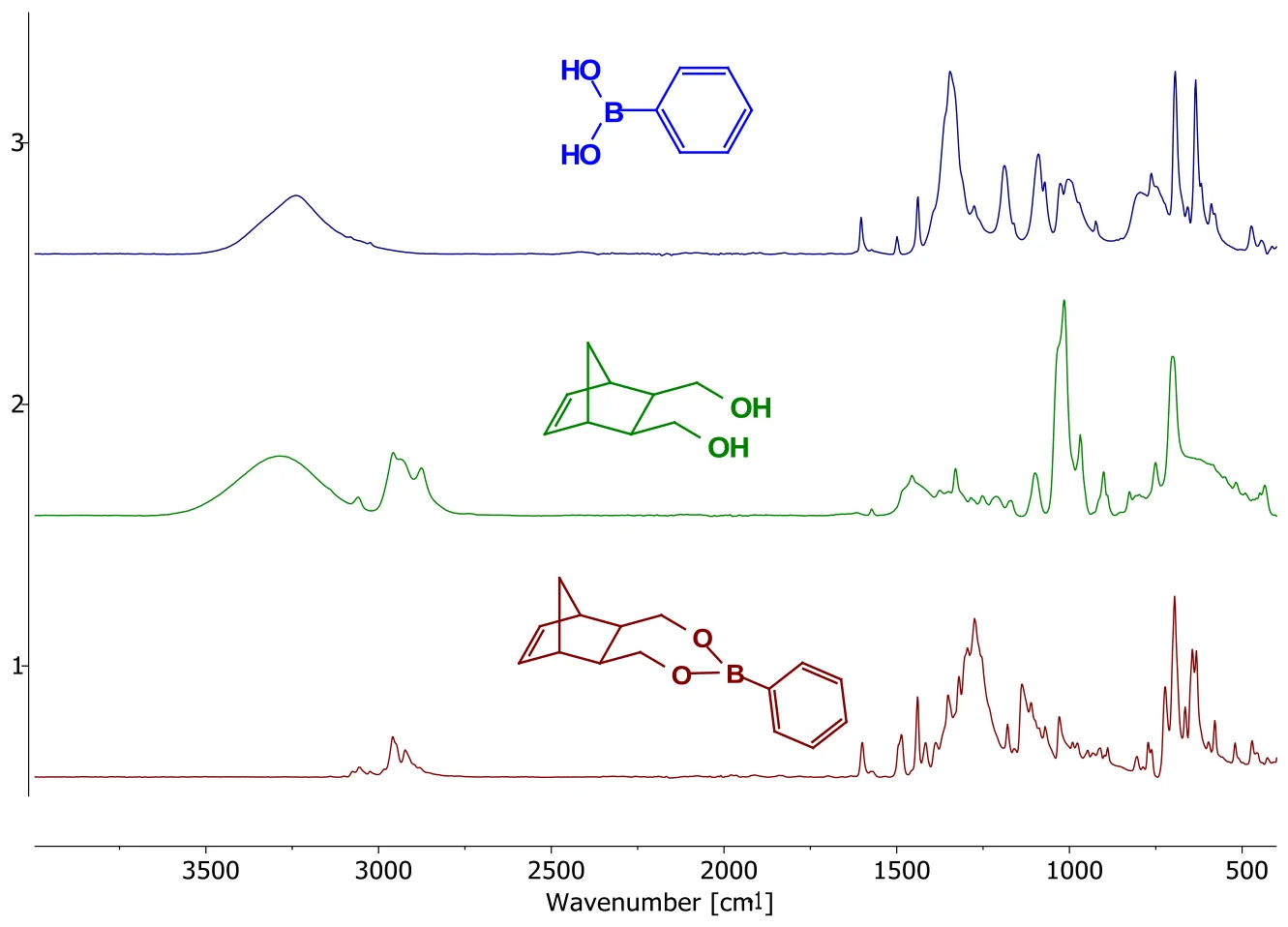
IR spectres comparison (blue line—phenylboronic acid, green line—NBE-diol, red line—compound M1).

The rest of the reactions required heating for full conversion of substrates. In each of the cases presented, the synthesis occurred rather rapidly, within 1–7 h, monitored by IR (Figure 1) and GC-MS analysis. More importantly, no by-products other than H_2_O were observed with the exception of M7, where vinylbiphenyl was formed and a purification process was required by washing the monomer with hexane and decanting the liquid phase, thus obtaining a much lower yield of desired product. All of the products formed crystalline solids easily soluble in organic polar solvents (DCM, CHCl_3_, THF) and a little less soluble in weakly polar ones (toluene). Analytical data along with NMR spectra are available in the Supplementary Materials.

**Scheme 3:**
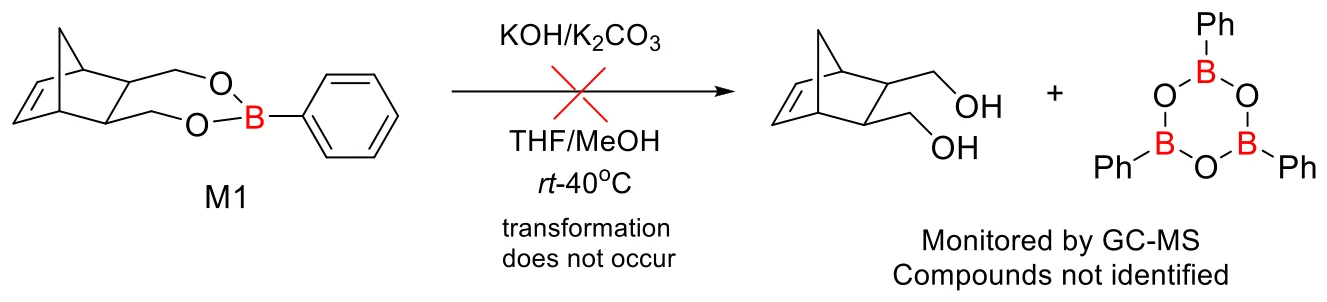
Hydrolysis test of M1.

Additional tests of M1 were carried out to verify the stability of B-O-C and B-C bonds in the resulting boronic esters. In small vials, M1 was dissolved in THF or MeOH with addition of K_2_CO_3_ or KOH and stirred for 24 h. This was followed by GC-MS analysis, which showed no signs of boronic ester decomposition—neither NBE-diol nor boroxine were present in the mixture (see Scheme 3).

The conducted studies of boronic esters indicate an exceptional stability in the presence of weak and strong bases. This phenomenon confirms the possible application of norbornene-based boron compounds in coordination process as sensors.

### 2.3. X-Ray Discussion

**Scheme 4:**
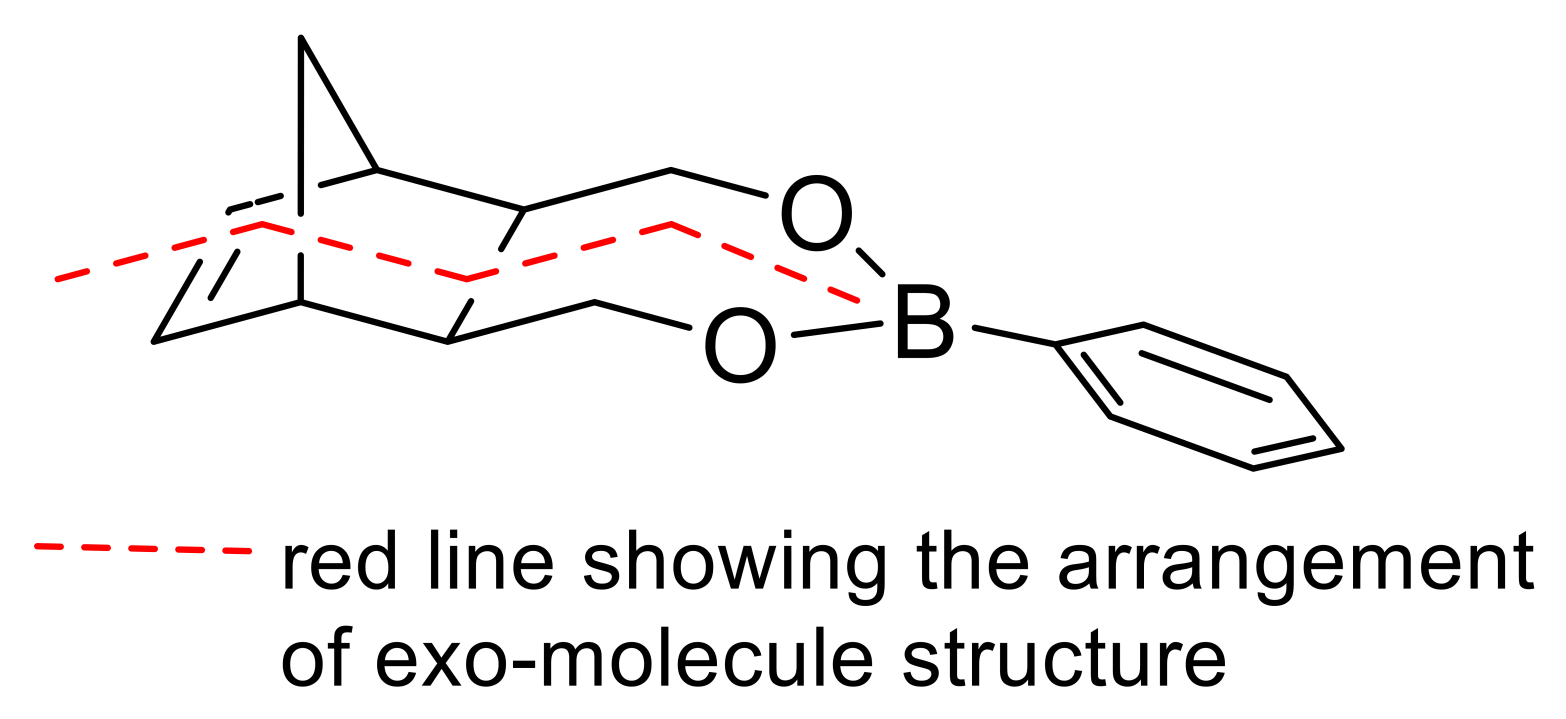
The structure of the M1 molecule, together with a line (red, dotted) showing the ideal conformation of the exo-isomer.

Furthermore, the solid-state structures of all monomers (M1-M7) were determined by X-ray crystallography (see Figures S2–S7 in the Supplementary Materials), according to Scheme 4. Crystals suitable for X-ray analysis were obtained by slow evaporation of CH_2_Cl_2_ at 3–5 °C [72]. As far as we know, there is only one example of crystallographic characterisation of a boron norbornene ester, the ferrocenyl *exo*-derivative [57].

**Figure 2:**
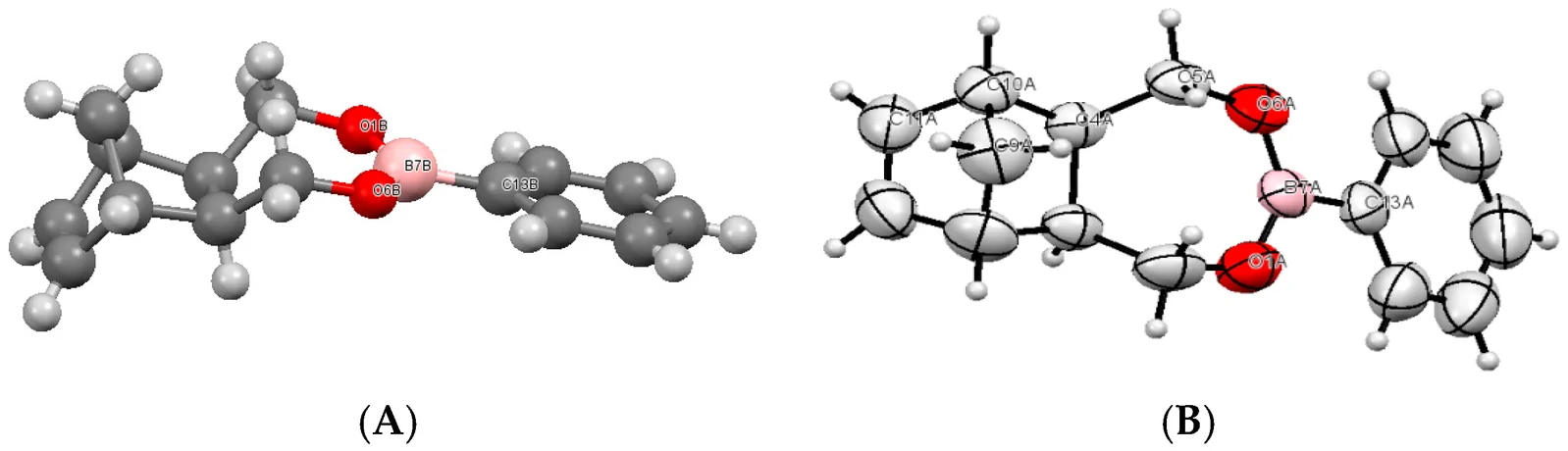
A perspective view of the independent molecule of M1 (**A**) and ORTEP view of M1 (**B**). Thermal ellipsoids are shown at 50% probability level. Selected bond length (Å) and angle (deg.): C11-C12 1.306(4), O-B-O 125.4(4), O-B-C 117.8(3) and 116.8(3), O6-B7 = 1.362(4) and 1.353(4), B7-C13 = 1.548(5).

One of the structures (**M1**) is shown below in Figure 2.

**Figure 3:**
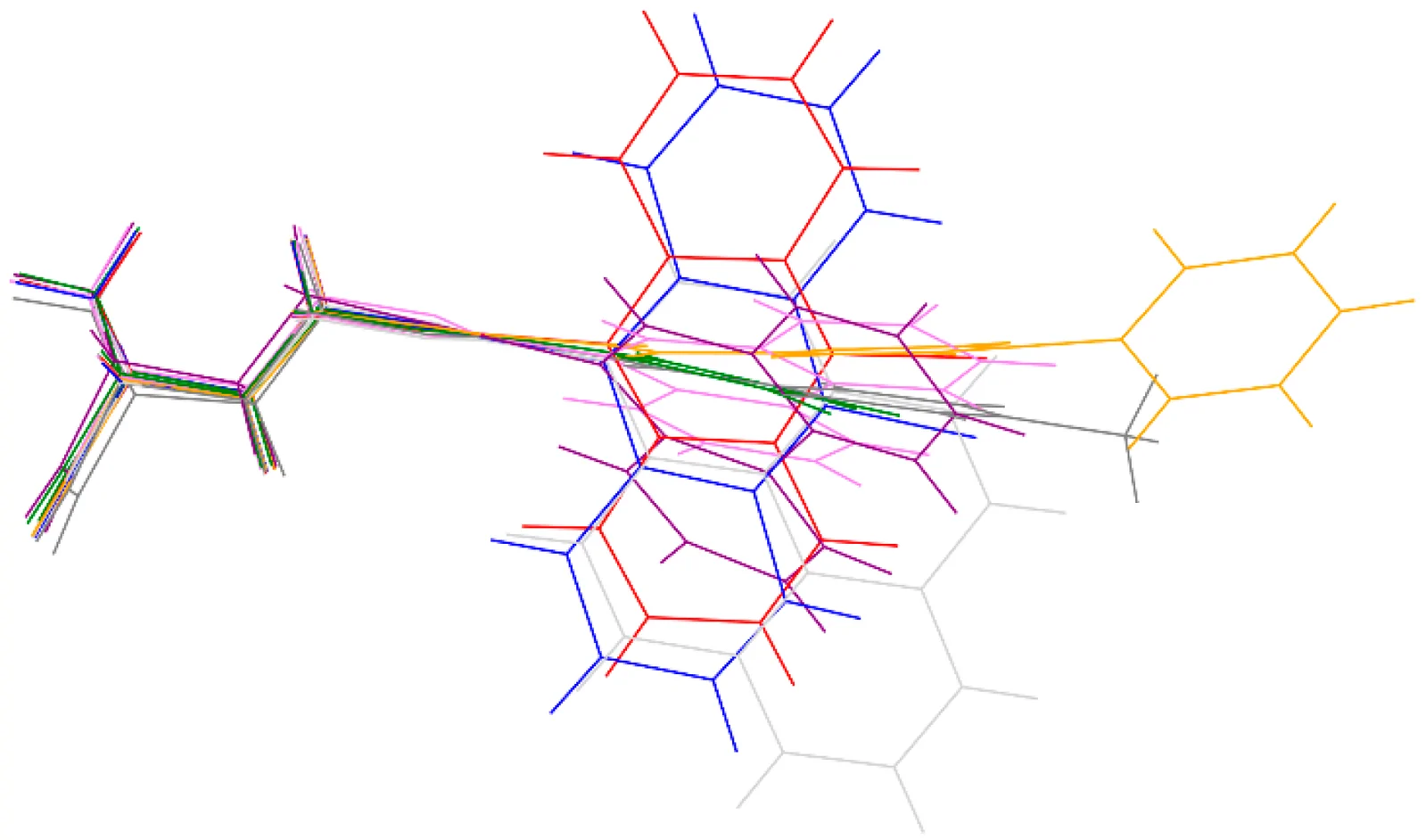
The superimposed X-ray projections for *exo*-monomers M1-M7. Different deviations of substituents at the boron atom while maintaining the *exo*-conformation of the norbornene fragment.

X-ray measurements of all monomers provided confirmation of the findings from previous spectroscopic analyses. Moreover, the X-ray projections for the compounds under discussion have been compiled, thus allowing clear observation of the differences and deviations occurring depending on the substituents at the boron atom, as illustrated in Figure 3. The different substituent positions on the boron atom in compounds M1-M7 did not impose significant steric hindrance on the vinylene fragment of the norbornene ring. Consequently, catalyst access to the double bond remained unhindered, facilitating the formation of the metallacyclobutane intermediate during the polymerisation.

Available identification methods confirmed that by appropriately designing the synthetic route, we obtained a group of *exo*-monomers that are very reactive in ring-opening metathesis polymerisation (ROMP). In order to verify this hypothesis, the following experiments were conducted.

### 2.4. ROMP Polymerisation of New exo-Norbornene-B-(arylene)dioxaborepines

The polymerisation process proceeded very rapidly with full conversion of monomers in most cases resulting in formation of solids upon precipitation from DCM in hexane. All of received polymers P1, P3, P5, P6, P7 were purified by successive precipitations in hexane and vacuum-dried. The resulting materials of P1, P5, P6, P7 showed good solubility in common organic solvents such as THF, CHCl_3_ and CH_2_Cl_2_. It was observed that during the polymerisation process of P3 the polymeric material started to precipitate from the mixture shortly after the introduction of the Grubbs initiator (around 3 min from the start). Furthermore, the resulting material showed difficulty in dissolving; however, upon mixing the material with CHCl_3_ and CH_2_Cl_2_, it was possible to dissolve the formed precipitate and purify it. Polymers P2 and P4 materials also precipitated during the reaction but were found to be insoluble. Optimisation of the reaction allowed us to obtain polymers with high yields, ranging from 88–98% in a short amount of time, mostly up to 3 h with full conversion of the monomers. Reaction conditions are available in Table 2. For analytical data and NMR spectra, see the Supplementary Materials.

**Figure 4:**
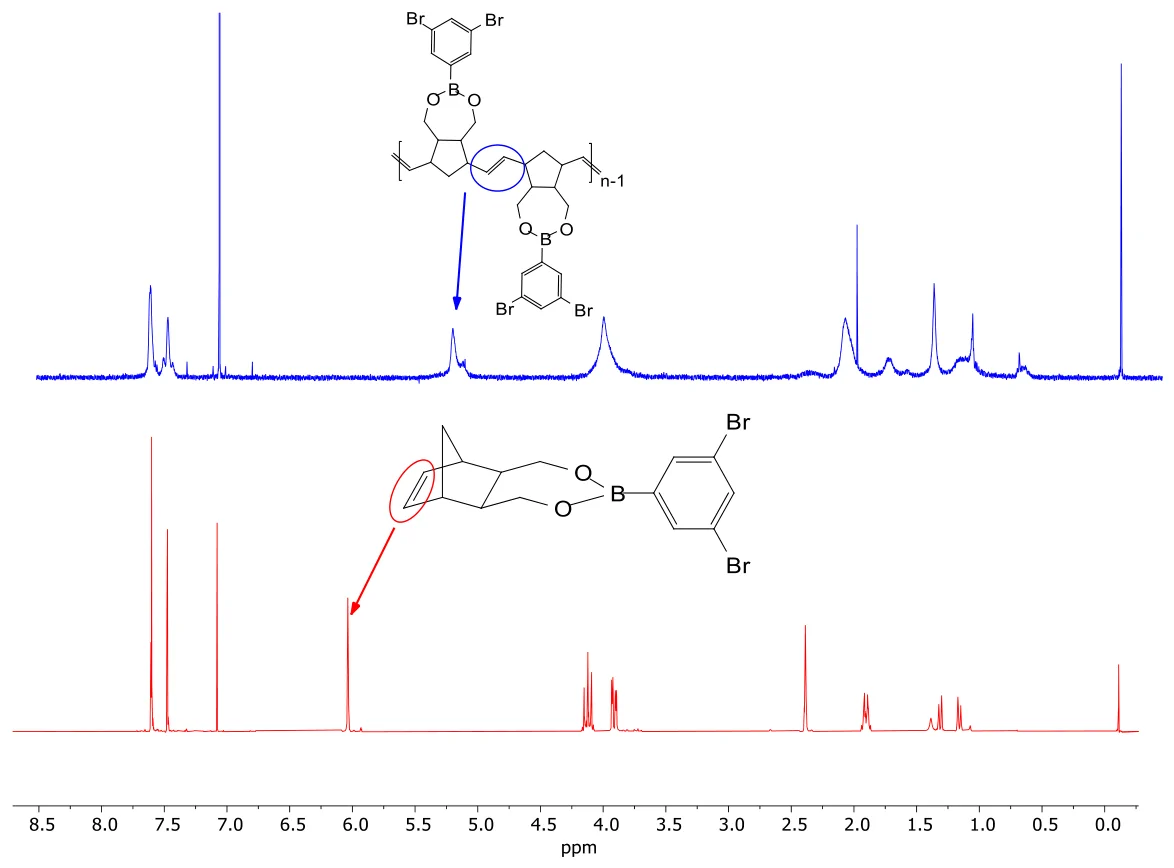
Monomer and polymer ^1^H NMR comparison.

As the reaction progressed, the rate of consumption of the monomer was observed by ^1^H NMR, and more specifically by the analysis of the vinylic region of norbornene unit. “Sharp” resonances at around 6.30–6.00 ppm of the monomer vinylene group (-HC=CH-norbornene) faded, while “broad” resonances coming from the inner vinylene groups of the polymer (-HC=CH-cyclopentane) at 5.50–5.00 ppm increased in intensity (see Figure 4).

In the case of soluble polymers P1, P3, P5, P6, P7, full consumption of the vinylene groups of monomers has been confirmed on ^1^H NMR spectra with no visible alkene resonances in 6.30–6.00 ppm region, as can be observed in the Supplementary Materials. During the course of the study, we noticed that the polymerisation process tended to proceed very rapidly. For monomer M1, additional examinations were performed. An increase in temperature resulted in the formation of an insoluble film-type product. The procedure was therefore modified, using only room temperature, also for the rest of the monomers. It was even observed that reducing the polymerisation time and changing the molar ratio of the catalyst ensured full conversion of monomer within 5 min (Table 3).

**Scheme 5:**
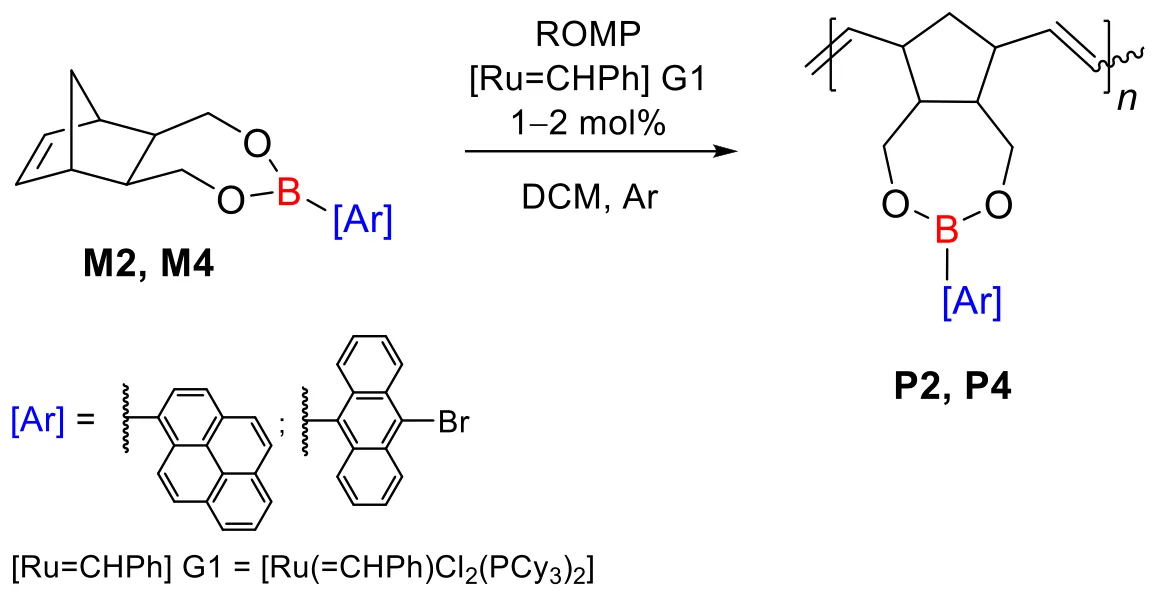
ROMP polymerisation of M2 and M4.

In the cases of P2 and P4, the products obtained *via* ROMP (see Scheme 5) were found to be insoluble in both polar and non-polar solvents (THF, DCM, toluene, EtOH, n-hexane), although the form of the final product resembled that of the other materials. Additional polymerisations under different conditions did not result in acquisition of soluble fractions of the polymers that could be used for NMR analysis. Other conditions included: temperature range (r.t.–40 °C), time (2 min–3 h), monomer/catalyst ratio (50:1, 100:1) and solvent volume (2.5; 3; 4; 5; 10 mL). The difficulties encountered during the polymerisation of P2 and P4 may be attributed to the bulky and rigid aromatic substituents containing a large number of aromatic rings, which could reduce the efficiency of chain propagation during ROMP. As a consequence, the formation of low-molecular-weight species and oligomeric fractions instead of well-developed polymer chains cannot be excluded. This may also be supported by the solubility difficulties of the P3 polymer.

**Figure 5:**
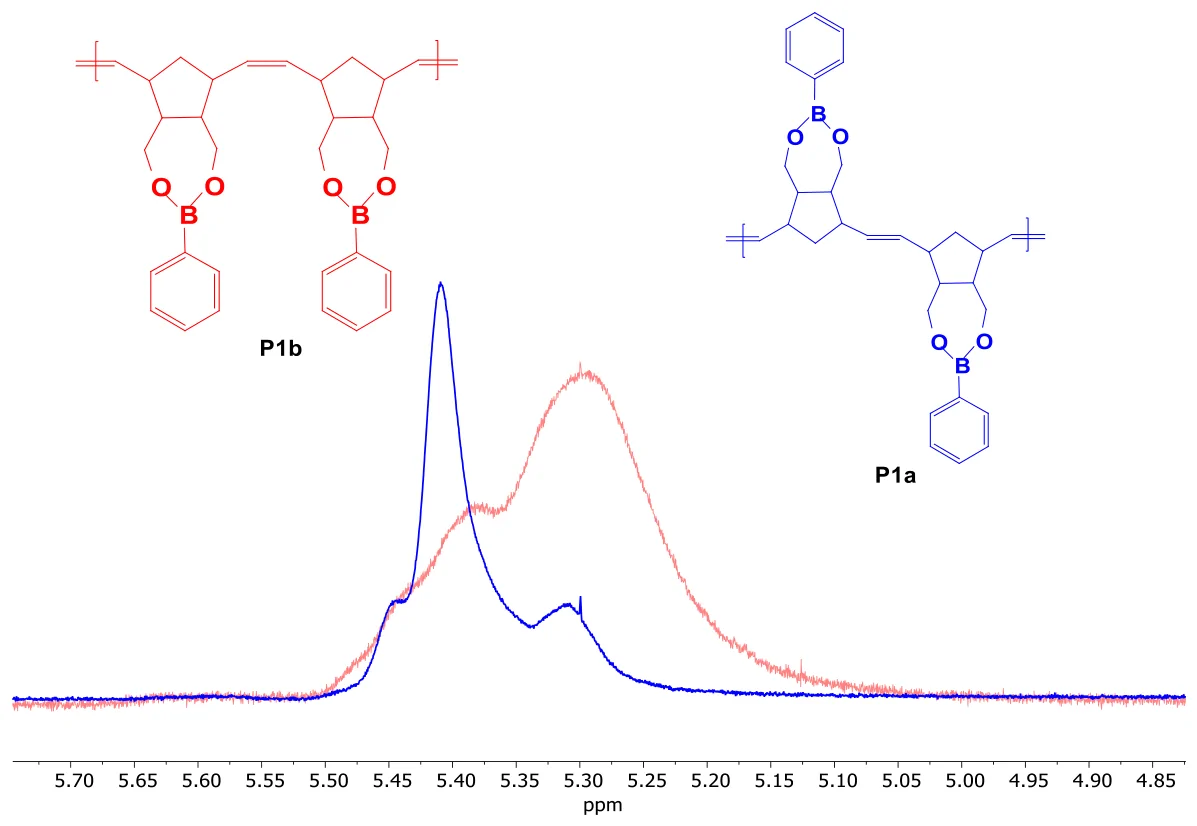
G1 (blue line)/G2 (red line) catalysts influence on *trans-*/*cis-* (*E*/*Z*) ratio of the polymeric chains.

Other studies have been carried out to investigate the effect of different generations of Grubbs catalyst on the structure of polymers (see Figure 5). In this case, two polymerisations P1a and P1b were carried out under similar conditions on M1. Interestingly, the use of the first generation (G1) in P1a resulted in formation of polymeric matrices with a predominantly *trans*-isomer (blue graph), while the use of the second generation (G2) in P1b gave mainly the *cis*-isomer (red graph), which can be observed on the ^1^H NMR spectra of both products. This examination confirms previous reports concerning the *E*/*Z* selectivity of ruthenium-based catalysts in metathesis-type processes [73]. The results of this experiment can be seen in Supplementary Materials.

Gel permeation chromatography with light-scattering detector (GPC/SEC-MALLS) in DCM showed good molecular values of the polymers (Table 4). In addition, the polymers had fairly narrow size distributions (*Đ_M_* = 1.6–2.1), with the exception of P3 (*Đ_M_* = 3.1), which was probably due to the short distance of the phenanthrene substituent from the poly(norbornene) matrix, providing more rigidity and steric hindrance in the structure affecting the polymer-chain conformation. The obtained number average molecular weights ranged from approximately M_n_ ≈ 0.75 × 10^4^–5.60 × 10^4^ g/mol while weight average molecular weights ranged from M_w_ ≈ 1.40 × 10^4^–9.15 × 10^4^ g/mol. Additional GPC measurements conducted on the P1 showed that the polymerisation time has a significant impact on the *Đ_M_* index. Shorter polymerisation (5 min) resulted in formation of polymeric chains with broader mass distribution, while the prolongation of the process (21 h) allowed the synthesis of chains bearing more similar masses. While polymers P2 and P4 were found to be insoluble, the GPC measurements were not feasible.

### 2.5. TGA Study

Further research has been conducted on the nature of thermal stability (TGA) on polymers P1–P7. For polymers P2 and P4, the insoluble fractions were investigated. TGA analysis showed 2–5 step weight loss process on heating up to 1000 °C under nitrogen atmosphere. Depending on the “hanging” functional group attached to the polymeric chain, the thermal stability differs in each case. The P1 polymer decomposed in the four step process starting from around 175 °C with a continuous degradation—the maximum weight loss was observed for temperature ranges: 175–332 °C (9% weight loss with maximum at 322 °C), 332–416 °C (maximum at 393 °C, 35% weight loss) and 416–510 °C (maximum at 434 °C, 27% weight loss), 510–626 °C (maximum at 536 °C, 4% weight loss). The lower thermal stability observed for P2 may be related to a lower degree of polymerisation achieved during ROMP and the possible formation of oligomeric species. Such low-molecular weight fractions are generally more susceptible to thermal degradation than high-molecular weight polymer chains. At 300 °C already a 20% weight loss was being observed and the weight losses were visible at temperature ranges: 115–196 °C (maximum at 158 °C, 5% weight loss), 196–329 °C (maximum at 308 °C, 21% weight loss), 329–400 °C (maximum at 372 °C, 34% weight loss), 400–513 °C (maximum at 430 °C, 16% weight loss), 513–855 °C (maximum at 641.0 °C, 10% weight loss). In the case of P3, the initial decomposition starts at around 154 °C and ends at 407 °C with 56% weight loss (maximum at 391 °C). The second step was seen between 407 and 511 °C (maximum at 435 °C, 24% weight loss). The P4 polymer, that also proved to be insoluble during the synthesis, showed multiple decomposition steps starting at temperature range 114–239 °C (maximum at 209 °C, 11% weight loss), followed by 239–393 °C (maximum at 337 °C, 56% weight loss), 393–506 °C (maximum at 403 °C, 8% weight loss), 506–609 °C (maximum at 539 °C, 3% weight loss). The decomposition of P5 polymer started from 140 to 239 °C (maximum at 203 °C, 1% weight loss), followed by 239–387 °C (maximum at 339 °C, 59% weight loss), 387–517 °C (maximum at 441 °C, 26% weight loss). Despite being one of the least thermally stable polymers, presumably due to the presence of bulky bromine substituents, it was the only sample that exhibited a less continuous degradation process and maintained its stability until the first maximum in the DTG curve was reached (see Supplementary Materials). Polymer P6 showed weight losses in the following temperature ranges: 171–347 °C (maximum at 319 °C, 8% weight loss), 347–515 °C (maximum at 428 °C, 65% weight loss). Polymer P7 started to decompose quickly and continuously from the start with weight losses in the following temperature ranges: 29–90 °C (maximum at 65 °C, 1% weight loss), 90–182 °C (maximum at 153 °C, 4% weight loss), 182–359 °C (maximum at 295 °C, 18% weight loss), 359–512 °C (maximum at 407 °C, 41% weight loss). The final residue yields were in the range 8.9–35.1 at 1000 °C. Results of the TGA measurements can be observed in Table 5 and in the Supplementary Materials.

To the best of our knowledge, there are no other similar linear materials with that organoboronic ester structure documented in the literature. However, when compared to cross-linked products containing siloxane-boronic ester fragments, the temperature value of the stable thermal “plato” range ends at temperatures of approximately 200–250 °C, and for compounds P1–P7 in the range from 60 °C to 135 °C (temperature range with a maximum mass loss of 0.2–0.3%). A significant proportion of the mass loss of P1-P7 polymeric materials commences at temperatures above 430 °C; however, minor mass losses have been observed at temperatures exceeding 900 °C. The products previously referenced undergo complete decomposition at temperatures ranging from 495 to 500 °C [74,75].

The thermal decomposition (TGA) of organic compounds under oxygen-free conditions is a complex process that can proceed in multiple directions, ultimately leading to the formation of various pyrolysis products such as gases, semi-volatile substances and carbon. It should be noted that the TGA process under discussion involves the decomposition of polymeric materials in a dry, inert nitrogen atmosphere, i.e., typical pyrolysis. Therefore, the stage of double-bond oxidation in the polynorbornene matrix should be disregarded. In consideration of the probable decomposition mechanism, the bond energies of boron-organic and organic compounds that demonstrate a fragmentary similarity to the compounds under analysis were taken into account. The bond energies of boron esters (B-O) range from 520 to 540 kJ/mol, boron-carbon (B-C) from 460 to 480 kJ/mol, and organic compounds (O-C) from 350 to 400 kJ/mol. The bond energies of carbon (single C-C) range from 350 to 410 kJ/mol, those of double C=C bonds from 610 to 630 kJ/mol, and those of C-H bonds from 413 kJ/mol [76]. A detailed analysis of the numerical energy values reveals that structural decomposition may proceed in the following order: simultaneous cleavage of single C-C carbon bonds and -O-C- ester bonds, followed by fragmentation of the polymer matrix. It has also been demonstrated that the carbon bonds of the matrix itself undergo cleavage. In the next stage, thermal cleavage of the boron-carbon bond (>B-C) originating from the substituent fragment at the boron atom occurs. For these groups of substituents, the decomposition process is initiated by the initial loss of a hydrogen atom from the aromatic rings and double bonds. This results in the formation of various additional hydrocarbons, such as methane or acetylene. Furthermore, the decomposition mechanism of the boron ester moiety, i.e., the -boron-oxygen-carbon- (-B-O-C-) bond, must be considered separately. This process is likely to cause dehydration and condensation, leading to the formation of boron anhydrides and boroxine, in addition to the formation of a protective char layer. The formation of the char is characterised by the cleavage of -B-O-C- bonds, resulting in the formation of boron oxide, for example, B_2_O_3_. Additionally, DSC measurements were performed. Detailed analysis of the temperature curves revealed no significant energy changes indicative of typical glass transition temperatures (T_g_).

### 2.6. Coordination Studies

**Scheme 6:**
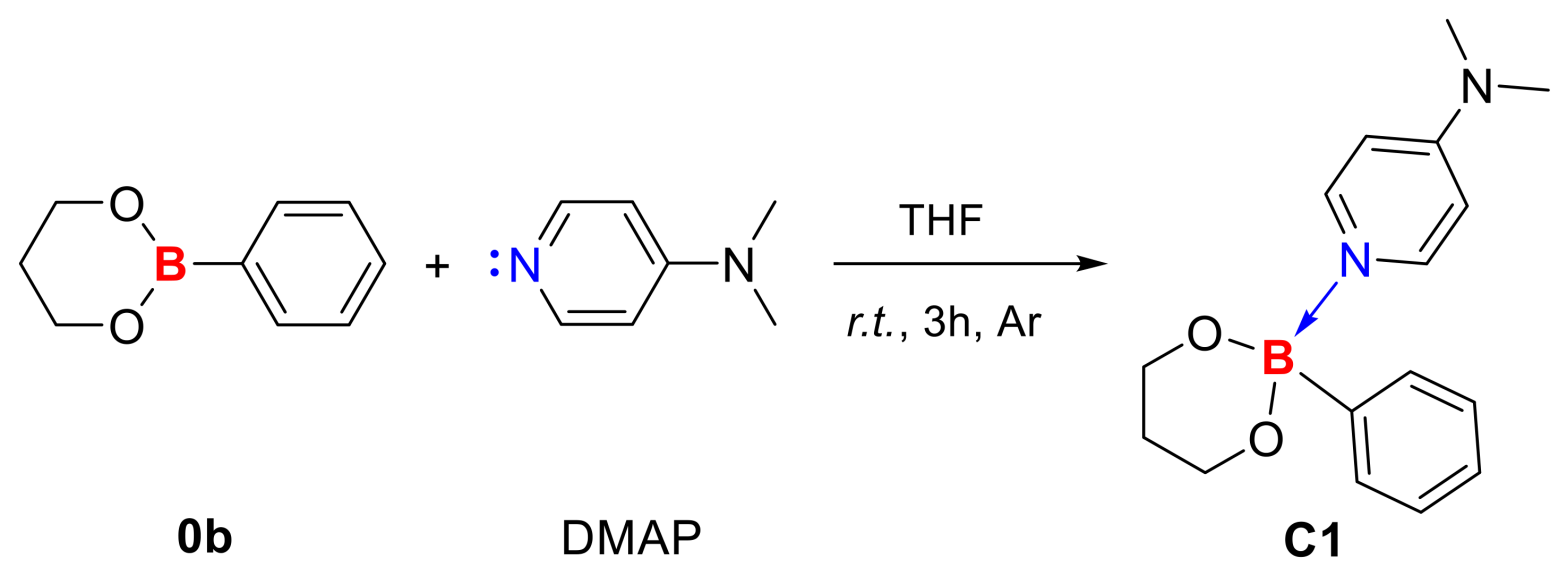
The coordination process of 0b with DMAP.

Coordination tests with amines have been performed to assess the applications of prepared polymeric materials as potential sensor systems. As previously stated, boron-based compounds usually exhibit Lewis acidic character that allows them to form coordination bonds with nucleophilic moieties. The studies started with tests on models 0a, 0b and monomer M1 mixed with different electron-donating compounds, such as pyridine, piperidine, or diisopropylamine in various solvents (toluene, diethyl ether, *n*-hexane, DCM). Eventually, optimisation of reaction conditions resulted in successful tests on model 0b using DMAP as the nucleophilic moiety with THF as a solvent according to Scheme 6.

**Figure 6:**
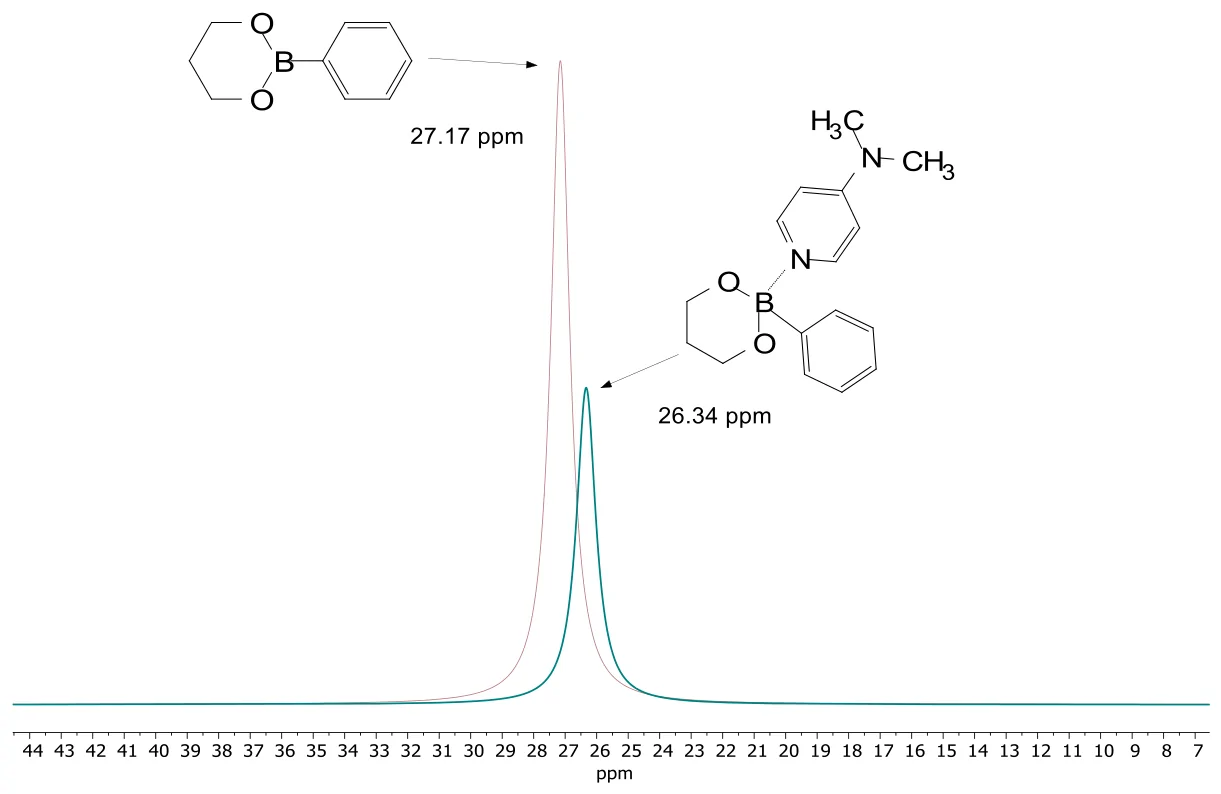
Coordination scheme and ^11^B NMR shifts comparison of model reaction in coordination process; uncoordinated boronic ester 0B (red line), coordinated boronic ester C1 (blue line).

The reason why DMAP coordinated may be attributed to cumulative effects, such as the Hammett’s equation or the resonance effect in the aromatic ring bearing additional electron-donating group (-N(CH_3_)_2_) in para- position which increased the activity of the aromatic ring, thus making it more basic (pKa = 9.70) from pyridine (pKa = 5.29) [77,78]. White crystals have been obtained as the product C1 of the described process, which was analysed using nuclear magnetic resonance (^1^H NMR, ^13^C NMR, ^11^B NMR). It was found that the coordination can be confirmed by the shift of the boron peak on the ^11^B NMR spectrum. In the case of this model (see Figure 6), a shift can be noted from 27.17 ppm (substrate) to 26.34 ppm (product).

**Scheme 7:**
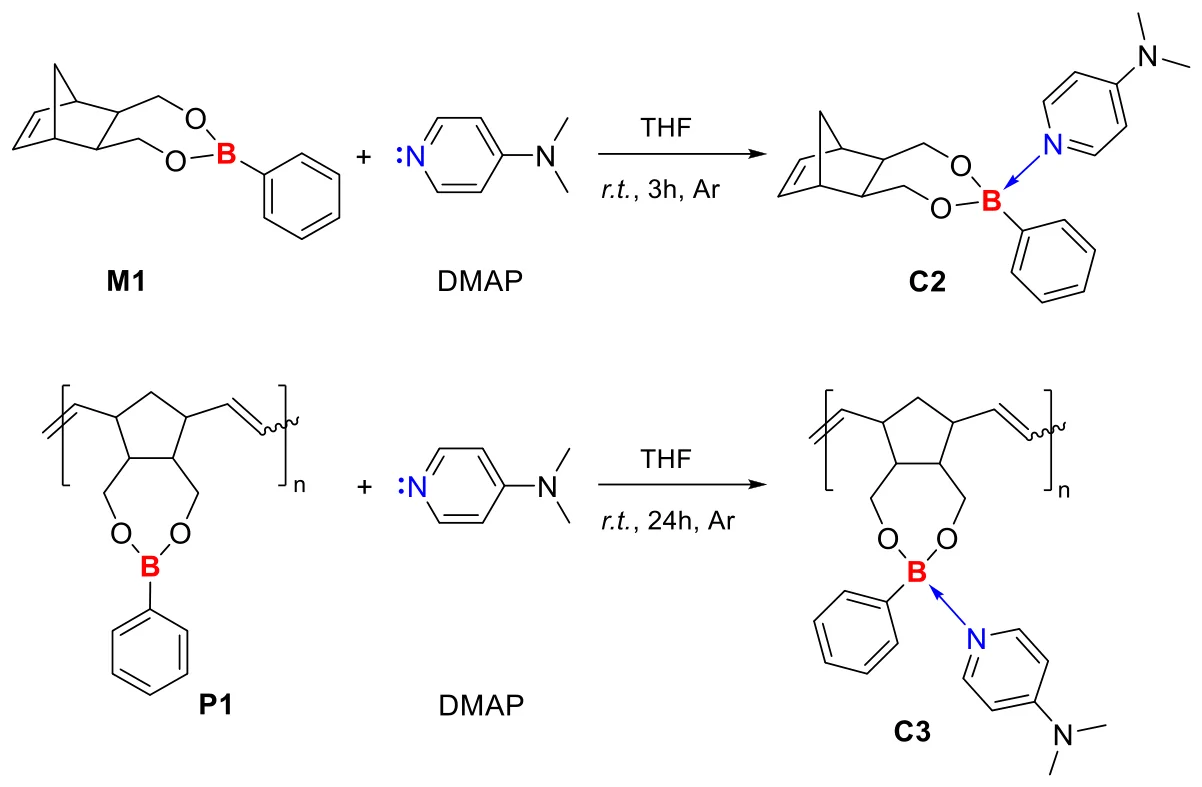
DMAP coordination effect on boron ester derivatives M1 and P1.

The positive results of the research on the coordination process of amino derivatives to boronic ester model 0b convinced us to use the optimized reaction conditions in tests with the exemplary monomer M1 and polymer P1, according to Scheme 7.

**Figure 7:**
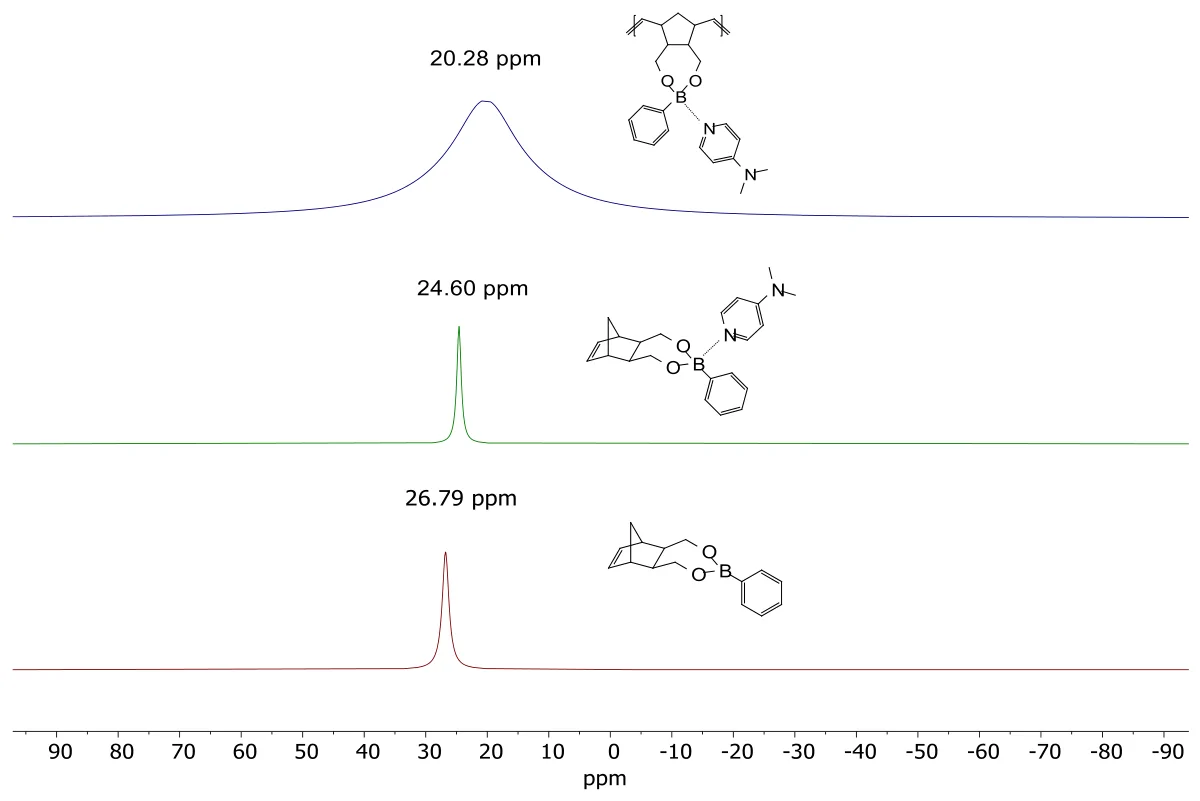
Coordination scheme and ^11^B NMR shifts of monomer (uncoordinated—red line), coordinated monomer C2 (green line), coordinated polymer C3 (blue line).

The research was therefore conducted on monomer M1 and polymer P1 using the same conditions as before. Once again, the shifts changed between the monomer (26.79 ppm), coordinated monomer C2 (24.60 ppm) and coordinated polymer C3 (20.28 ppm), see Figure 7.

**Figure 8:**
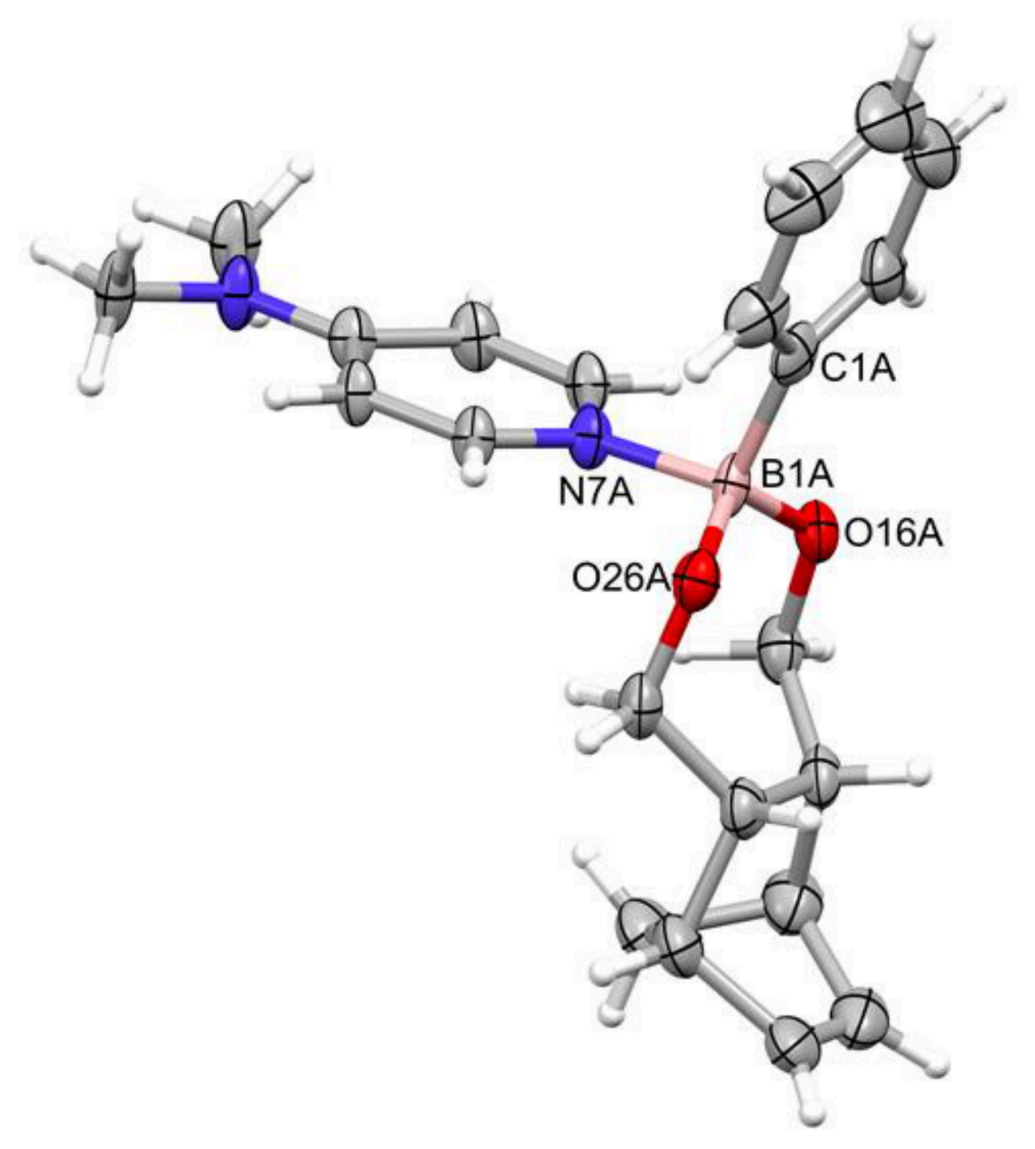
A perspective view of the independent molecule of C2 as an ORTEP view. Thermal ellipsoids are shown at 50% probability level. Selected bond length (Å) and angle (deg.): C11-C12 1.315(5), O-B-O 117.1(2), B-N 166.0(3), O-B-C 109.5(2) and 110.3(2), O6-B7 = 1.445(3) and 1.451(3), B1-C1 = 1.623(4).

Furthermore, the coordination effect was corroborated by X-ray structural studies with monomer M1 and 4-dimethylaminopyridine (DMAP). Crystals suitable for X-ray analysis were obtained by slow evaporation of a toluene solution of C2 at 3–5 °C [79]. This measurement indicates the existence of a coordination bond N→B, formed by the endocyclic nitrogen atom, see Figure 8.

Furthermore, this study provides compelling evidence that, in the resulting molecule, DMAP arranged almost perpendicularly to the norbornene fragment and the phenyl substituent bonded to the boron atom, as illustrated in Figure 8.

## 3. Materials and Methods

### 3.1. Materials

The chemicals were obtained from the following sources: toluene (99.8%), methylene chloride (DCM, 99.8%), n-hexane (99.5%), tetrahydrofuran (99.8%) were purchased from Chempur (Piekary, Poland) and P.O.Ch. Gliwice (Gliwice, Poland), 5-norbornene-2-*exo*-3-*exo*-dimethanol (>97%), ethylene glycol (>99%), 1,3-propanediol (>99%), phenylboronic acid (95%), pyrene-1-boronic acid (95%), 9-phenantracenylboronic acid (97%), 10-bromoanthracene-9-boronic acid (97%), 3,5-dibromophenylboronic acid (98%), trans-2-(4-methylphenyl)vinylboronic acid (97%), trans-2-(4-biphenyl)vinylboronic acid (95%), pyridine (99%) and 4-dimethylaminopyridine (99%), deuterated chloroform (CDCl_3_) (>99.8%), Grubbs catalyst 1st generation C_43_H_72_Cl_2_P_2_Ru [RuCl_2_(=CHPh)(PCy_3_)_2_], Grubbs catalyst 2nd generation C_46_H_65_Cl_2_N_2_PRu [RuCl_2_(=CHPh)(PCy_3_)(IMesH_2_)], from Aldrich (St. Louis, MO, USA), ethyl vinyl ether (99%) from Merck (Darmstadt, Germany), magnesium sulphate (MgSO_4_) from EuroChem (Zug, Switzerland). Acquired solvents—toluene, methylene chloride, tetrahydrofuran and n-hexane were stored over molecular sieves, type A4. Methylene chloride used in polymerisation was kept in Schlenk’s system in inert atmosphere over molecular sieves type A4.

### 3.2. Synthetic Procedures

#### 3.2.1. General Procedure for the Synthesis of Norbornene-B-(arylene)-dioxaborepines

A two-neck round bottom flask equipped with magnetic stirring bar was charged with 5-norbornene-2-exo-3-exo-dimethanol, toluene and adequate boronic acid. Depending on the type of boronic acid used, the reaction was then heated in an oil bath for a specific amount of time. The progress of the reaction was monitored by FT-IR and GC-MS analyses. For isolation, the reaction mixture was cooled at room temperature and magnesium sulphate was inserted in order to remove any trace of water in the setup. After 3 h, the mixture was filtered through G4 funnel, the organic solvent was removed by rotary evaporator to obtain the desired product. NMR spectroscopic analysis confirmed the formation of the expected products. For details concerning preparation, see the Supplementary Materials.

#### 3.2.2. General Procedure of Ring-Opening Metathesis Polymerisation Reaction (ROMP) of exo-Norbornene-B-(arylene)-dioxaborepines

A two-neck glass reactor under argon equipped with magnetic stirring bar was loaded with adequate monomer (M1–M7), methylene chloride, 1st or 2nd generation Grubbs catalyst. The molar ratio between the reactants was as follows: [olefin]:[Ru=CHPh] = 50:1, 100:1 or 200:1. The solution was stirred for a certain amount of time at room temperature, after which an excess (≈150 μL) of ethyl vinyl ether was added in order to terminate the reaction. The obtained mixture was then concentrated and polymeric materials were precipitated by adding the rest of the solution dropwise to the n-hexane. In the next step, the hexane was decanted and the polymer was dissolved once again in methylene chloride and then precipitated. This step was repeated to remove any residual catalyst that may have remained in the mixture. Finally, polymer was dried under vacuum. NMR spectroscopic analysis confirmed the conversion of the monomer along with formation of expected polymer. For details concerning preparation, see the Supplementary Materials.

### 3.3. General Procedure of Amine Coordination Process

Monomer/polymer/model was dissolved in THF in a glass vial equipped with magnetic stirring bar. Upon full dissolution, DMAP was added and the mixture was stirred for 3–24 h, after which the solvent was evaporated under vacuum. The products were identified by NMR and X-ray studies. For details concerning preparation, see the Supplementary Materials.

## 4. Conclusions

An effective, simple method of obtaining boronic esters has been developed with formation of previously not reported materials. An additional optimisation of the condensation reaction was performed, acquiring high isolation yields of products. Most of the monomers presented herein proved to be prone to the ring-opening metathesis polymerisation process. More importantly, through the modification of conditions, it was possible to control the resulting polymeric chains length and mass. Supplementary investigation has also revealed the impact of the chosen catalysts on the structure of polymeric chains and their *E*/*Z* ratio. Additional research confirmed the possibility of nucleophilic coordination with boronic esters both in monomer and polymer forms. All the structural features mentioned above form the basis for future physico-chemical studies of the materials obtained, with a view to developing optical sensors or photo-induced systems based on polymers of this type. Furthermore, the presented research findings on the coordination observed at the Lewis centre formed by the boron atom point to a new application: materials capable of capturing amino-derived pollutants from aquatic environments or for the design of layered systems. In the future, it might also be of interest to expand the library of compounds to include other *p*-block elements, such as phosphorus or germanium. Furthermore, it would be advisable to explore other modifications towards biologically active boron ester derivatives.

## Figures and Tables

**Table 1:** Condensation reaction of diol and boronic acids.

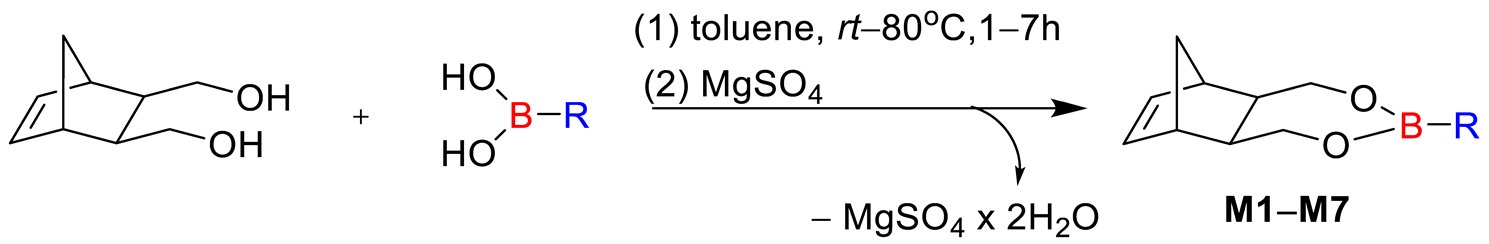				
Entry	R	T [°C]	Time [h]	Isolated Yield [%]
M1	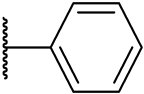	rt	3	99
M2	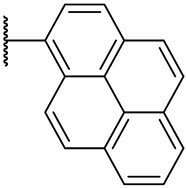	75	7	97
M3	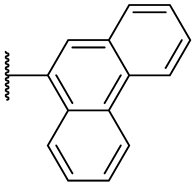	80	6	97
M4	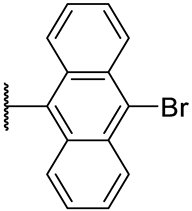	80	6	97
M5	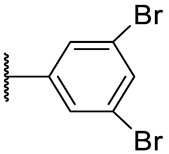	80	4	98
M6	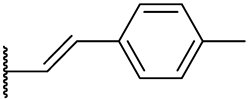	40	1	95
M7	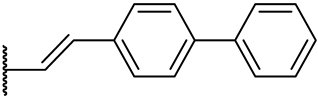	40	2	54

**Table 2:** ROMP of norbornene-B-(arylene)-dioxaborepines ^[a]^.

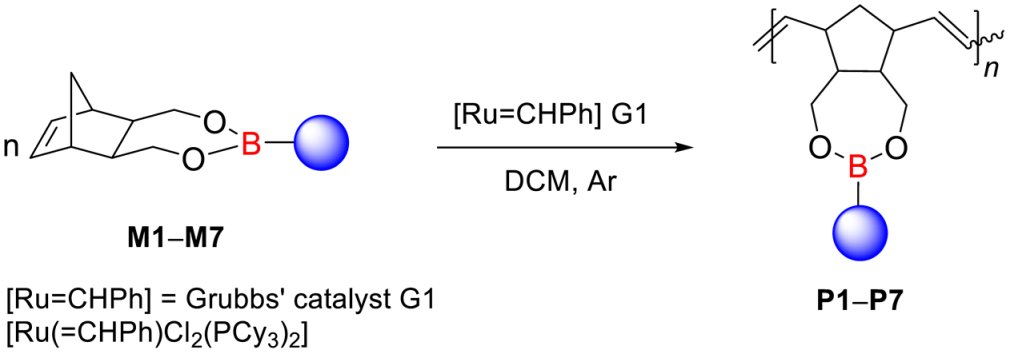				
Entry	Monomer	Time [h]	Molar Ratio [Mon]:[Cat.]	Isolated Yield [%]
P1	M1	2.5	100:1	97
P2 ^[b,c]^	M2	1	50:1	97
P3	M3	3	100:1	97
P4 ^[b,c]^	M4	0.5	100:1	99
P5	M5	2.5	100:1	98
P6	M6	1	100:1	90
P7	M7	1	100:1	88

^[a]^ Reaction conditions: DCM, room temperature, G1 [Ru(=CHPh)Cl_2_(PCy_3_)_2_]. ^[b]^ Material insoluble (undetermined structure by NMR). ^[c]^ TGA measurement performed.

**Table 3:** ROMP of M1 in the presence of G1 Grubbs initiator ^[a]^.

Test Number	Molar Ratio [Mon]:[Cat.]	T [°C]	Vol. [mL]	Time [h]	Product
1	200:1	40	3.5	3	film
2	200:1	rt	1	21	Polymer
3	200:1	rt	2	3	Polymer
4	100:1	rt	11	2.5	Polymer
5	100:1	rt	2	0.12	Polymer

^[a]^ Reaction conditions: DCM, G1, conversion 100%.

**Table 4:** GPC results—mass parameters ^[a]^.

Polymer	Time [h]	*M_n_* (×10^4^)	*M_w_* (×10^4^)	*Đ_M_* (*M_w_*/*M_n_*)
P1 ^[b]^	21.0	5.60	9.15	1.6
P1	0.12	3.95	8.15	2.1
P3	3.0	2.15	6.60	3.1
P5	2.5	5.05	8.50	1.7
P6	1.0	1.15	2.45	2.1
P7	1.0	0.75	1.40	1.8

^[a]^ Conditions: DCM, polystyrene standards, 0.8 mL/min, T = 30 °C, *Đ_M_* (as a PDI = *M_W_*/*M_n_*). Molar ratio–[cat.]:[monomer] = 1:100, except P1; ^[b]^ [cat.]:[mon.] = 1:200; GPC analysis results, multiplied by the factor x 0.5, recalculated according to the literature recommendations, are the actual mass parameters (see Supplementary Materials p. 51).

**Table 5:** TGA results ^[a]^.

Polymer	Td^5^ (°C)	Td^10^ (°C)	First Step of Thermal Decomposition (Weight Loss (%)) Top Temp. (°C)	Second Step of Thermal Decomposition (Weight Loss (%)) Top Temp. (°C)	Third Step of Thermal Decomposition (Weight Loss (%)) Top Temp. (°C)	Fourth Step of Thermal Decomposition (Weight Loss (%)) Top Temp. (°C)	Fifth Step of Thermal Decomposition (Weight Loss (%)) Top Temp. (°C)
P1	292.8	336.6	174.9–331.6 (8.7) Max. 322.4	331.6–416.3 (34.7) Max. 393.6	416.3–510.4 (26.5) Max. 433.9	510.4–626.1 (4.0) Max. 536.1	----
P2	183.4	242.3	115.1–196.4 (5.1) Max. 158.0	196.4–328.9 (20.6) Max. 308.3	328.9–399.5 (33.9) Max. 372.4	399.5–513.1 (15.5) Max. 430.1	513.1–854.7 (9.9) Max. 641.0
P3	290.3	351.2	154.1–406.9 (55.9) Max. 391.1	406.9–511.1 (24.1) Max. 434.8	----	----	----
P4	182.0	220.9	113.7–239.4 (10.7) Max. 208.9	239.4–393.4 (56.1) Max. 337.0	393.4–505.7 (8.0) Max. 402.5	505.7–608.6 (2.8) Max. 539.4	----
P5	312.9	321.3	139.9–238.8 (0.6) Max. 202.6	238.8–387.4 (59.0) Max. 338.5	387.4–516.5 (25.7) Max. 441.1	----	----
P6	300.8	360.4	170.9–347.0 (8.2) Max. 319.9	347.0–514.5 (65.0) Max. 428.0	----	----	----
P7	197.9	263.0	29.0–89.5 (0.7) Max. 65.2	89.5–181.6 (3.6) Max. 152.9	181.6–359.1 (18.2) Max. 295.4	359.1–511.8 (40.5) Max. 407.0	----

^[a]^ Conditions: determined by TGA analysis: N_2_, 10 °C/min; 25–1000 °C; total weight loss (%): P1 (~84), P2 (~86), P3 (~86), P4 (~85), P5 (~91), P6 (~78), P7 (~64) up to 981 °C.

## Data Availability

The original contributions presented in this study are included in the article/Supplementary Material. Further inquiries can be directed to the corresponding author.

## Supplementary Materials

The following supporting information can be downloaded at: https://www.mdpi.com/article/10.3390/molecules31183151/s1, X-ray structures of monomers: M1 as Figure S1, M2 as Figure S2, M3 as Figure S3, M4 as Figure S4, M5 as Figure S5, M6 as Figure S6, M7 as Figure S7; NMR Spectra (1H NMR, 13CNMR, 11B NMR) of preliminary model compounds: 0a as Figures S8–S9, 0b as Figures S10–S12, Monomers: M1 as Figures S13–S15, M2 as Figures S16–S18, M3 as Figures S19–S21, M4 as Figures S22–S24, M5 as Figures S25–S27, M6 as Figures S28–S30, M7 as Figures S31–S33, Polymers: P1/P1a as Figures S34–S36, P1b as Figure S37, P3 as Figures S38–S40, P5 as Figures S41–S43, P6 as Figures S44–S46, P7 as Figures S47–S49, Coordinated compounds: C1 as Figures S50–S52, C2 as Figures S53–S55, C3 as Figures S56–S58; GPC analysis for polymers: P1 as Figures S59 and S60, P3 as Figure S61, P5 as Figure S62, P6 as Figure S63, P7 as Figure S64; TGA curves for polymers: P1 as Figure S65, P2 as Figure S66, P3 as Figure S67, P4 as Figure S68, P5 as Figure S69, P6 as Figure S70, P7 as Figure S71; Combined X-ray data as: Tables S1–S3 [80,81,82,83,84,85,86,87].

## Abbreviations

ROMP	Ring-opening metathesis polymerisation
*Đ* *_M_*	Polydispersity index (as a PDI = *M_W_*/*M_n_*)
TLC	Thin-layer chromatography
NMR	Nuclear magnetic resonance
GC-MS	Gas chromatography/mass spectrometry
FT-IR	Fourier transform infrared
TGA	Thermogravimetric analysis
PCy_3_	Tricyclohexylphosphine
DMAP	4-dimethylaminopyridine
THF	Tetrahydrofuran
G4 funnel	G4 glass filter, with a pore size of 10 to 16 micrometres (μm)

## References

[1] Kaplan S.W., Zhukhovitskiy A.V. (2025). Recent advances in ring-opening metathesis polymerizations. Prog. Polym. Sci..

[2] Klavetter F.L., Grubbs R.H. (1988). Polycyclooctatetraene (polyacetylene): Synthesis and properties. J. Am. Chem. Soc..

[3] Edwards J.H., Feast W.J., Bott D.C. (1984). New routes to conjugated polymers: 1. A two step route to polyacetylene. Polymer.

[4] Basyouni M.Z., Abdu M.E., Radwan M.F., Spring A.M. (2024). From monomer to polymer: Controlled synthesis and comprehensive analysis of poly(p-phenylene vinylene) via ROMP. J. Mol. Struct..

[5] Jozefiak T.H., Ginsburg E.J., Gorman C.B., Grubbs R.H., Lewis N.S. (1993). Voltammetric characterization of soluble polyacetylene derivatives obtained from the ring-opening metathesis polymerization (ROMP) of substituted cyclooctatetraenes. J. Am. Chem. Soc..

[6] Kang H.A., Bronstein H.E., Swager T.M. (2008). Conductive Block Copolymers Integrated into Polynorbornene-Derived Scaffolds. Macromolecules.

[7] Nantalaksakul A., Krishnamoorthy K., Thayumanavan S. (2010). Broadening Absorption in Conductive Polymers through Cross-linkable Side Chains in a Nonconjugated Polymer Backbone. Macromolecules.

[8] Bazan G.C., Khosravi E., Schrock R.R., Feast W.J., Gibson V.C., O’Regan M.B., Thomas J.K., Davis W.M. (1990). Living ring-opening metathesis polymerization of 2,3-difunctionalized norbornadienes by Mo(:CHBu-tert)(:NC_6_H_3_Pr-iso2-2,6)(OBu-tert)_2_. J. Am. Chem. Soc..

[9] Flook M.M., Gerber L.C.H., Debelouchina G.T., Schrock R.R. (2010). Z-Selective and Syndioselective Ring-Opening Metathesis Polymerization (ROMP) Initiated by Monoaryloxidepyrrolide (MAP) Catalysts. Macromolecules.

[10] Makovetsky K.L., Finkel’Shtein E.S., Ostrovskaya I.Y., Portnykh E.B., Gorbacheva L.I., Golberg A.I., Ushakov N.V., Yampolsky Y.P. (1992). Ring-opening metathesis polymerization of substituted norbornenes. J. Mol. Catal..

[11] Miao Y., Bazan G.C. (1994). Paracyclophene Route to Poly(p-phenylenevinylene). J. Am. Chem. Soc..

[12] Spring A.M., Yu C.Y., Horie M., Turner M.L. (2009). MEH-PPV by microwave assisted ring-opening metathesis polymerisation. Chem. Commun..

[13] Lidster B.J., Behrendt J.M., Turner M.L. (2014). Monotelechelic poly(p-phenylenevinylene)s by ring opening metathesis polymerisation. Chem. Commun..

[14] Feast W.J., Gibson V.C., Johnson A.F., Khosravi E., Mohsin M.A. (1997). Well-defined graft copolymers via coupled living anionic and living ring opening metathesis polymerisation. J. Mol. Catal. A Chem..

[15] Lin T.-P., Chang A.B., Chen H.-Y., Liberman-Martin A.L., Bates C.M., Voegtle M.J., Bauer C.A., Grubbs R.H. (2017). Control of Grafting Density and Distribution in Graft Polymers by Living Ring-Opening Metathesis Copolymerization. J. Am. Chem. Soc..

[16] Chang A.B., Lin T.-P., Thompson N.B., Luo S.-X.L., Liberman-Martin A.L., Chen H.-Y., Lee B., Grubbs R.H. (2017). Design, Synthesis, and Self-Assembly of Polymers with Tailored Graft Distributions. J. Am. Chem. Soc..

[17] Liu J., Liao Y., He X., Yu J., Ding L., Xie M. (2010). Facile One-Pot Approach for Preparing Functionalized Polymeric Nanoparticles via ROMP. Macromol. Chem. Phys..

[18] Le D., Montembault V., Pascual S., Collette F., Héroguez V., Fontaine L. (2013). Synthesis of 1,4-polybutadiene-g-poly(ethylene oxide) via the macromonomer approach by ROMP. Polym. Chem..

[19] Hanik N., Kilbinger A.F.M. (2013). Narrowly distributed homotelechelic polymers in 30 minutes: Using fast in situ pre-functionalized ROMP initiators. J. Polym. Sci. Part A Polym. Chem..

[20] Torres-Rocha O.L., Wu X., Zhu C., Crudden C.M., Cunningham M.F. (2019). Synthesis of Diblock and Triblock Polymers from Cyclooctadiene and Norbornene Via ROMP in Miniemulsion. Macromol. Rapid Commun..

[21] Brown H.C., Basavaiah D., Bhat N.G. (1983). General synthesis of alkylalkenylalkynylboranes via haloboranes. Organometallics.

[22] Gómez-Jaimes G., Barba V. (2014). Boronate esters: Synthesis, characterization and molecular base receptor analysis. J. Mol. Struct..

[23] Currie G.S., Drew M.G.B., Harwood L.M., Hughes D.J., Luke R.W.A., Vickers R.J. (2000). Chirally templated boronic acid Mannich reaction in the synthesis of optically active α-amino acids. J. Chem. Soc. Perkin Trans. 1.

[24] Wiecko J., Sherman W.R. (1976). Boroacetylation of carbohydrates. Correlations between structure and mass spectral behavior in monoacetylhexose cyclic boronic esters. J. Am. Chem. Soc..

[25] Ahmed A., Mushtaq I., Chinnam S. (2023). Suzuki–Miyaura cross-couplings for alkyl boron reagent: Recent developments—A review. Future J. Pharm. Sci..

[26] Gao Y., Hu J., Teng J., Hang G., Li L., Zheng S. (2024). Crosslinking of polyurethane with boronic ester bonds: An impact of B–N coordination. Polymer.

[27] Icli B., Sheepwash E., Riis-Johannessen T., Schenk K., Filinchuk Y., Scopelliti R., Severin K. (2011). Dative boron–nitrogen bonds in structural supramolecular chemistry: Multicomponent assembly of prismatic organic cages. Chem. Sci..

[28] Yuan C., Chang Y., Mao J., Yu S., Luo W., Xu Y., Thayumanavan S., Dai L. (2015). Supramolecular assembly of crosslinkable monomers for degradable and fluorescent polymer nanoparticles. J. Mater. Chem. B.

[29] Luisier N., Scopelliti R., Severin K. (2016). Supramolecular gels based on boronate esters and imidazolyl donors. Soft Matter.

[30] Kim C., Ejima H., Yoshie N. (2017). Non-swellable self-healing polymer with long-term stability under seawater. RSC Adv..

[31] Contreras R. (1994). Diethanolamines, Diphenolamines, Diethylenetriamines, the Game with Phosphorus and Boron. Phosphorus Sulfur Silicon Relat. Elem..

[32] Seçkin Z.E., Volkan M. (2005). Flow injection fluorescence determination of dopamine using a photo induced electron transfer (PET) boronic acid derivative. Anal. Chim. Acta.

[33] Jun E.J., Liub H., Choi J.Y., Lee J.Y., Yoon J. (2013). New fluorescent receptor composed of two imidazoliums, two pyrenes and a boronic acid for the recognition of DOPAC. Sens. Actuators B.

[34] Li Y., Xie Y., Qin Y. (2014). Polymeric membrane sensors with boronic acid functionalized boron dipyrromethene for selective measurement of dopamine. Sens. Actuators B.

[35] Sandanayake K.R.A.S., Shinkai S. (1994). Novel molecular sensors for saccharides based on the interaction of boronic acid and amines: Saccharide sensing in neutral water. J. Chem. Soc. Chem. Commun..

[36] James T.D., Sandanayake K.R.A.S., Iguchi R., Shinkai S. (1995). Novel Saccharide-Photoinduced Electron Transfer Sensors Based on the Interaction of Boronic Acid and Amine. J. Am. Chem. Soc..

[37] Tong A.J., Yamauchi A., Hayashita T., Zhang Z.Y., Smith B.D., Teramae N. (2001). Boronic Acid Fluorophore/β-Cyclodextrin Complex Sensors for Selective Sugar Recognition in Water. Anal. Chem..

[38] Yu C., Yam V.W.W. (2009). Glucose sensing via polyanion formation and induced pyrene excimer emission. Chem. Commun..

[39] Lopez C.S., Huvelle M.A.L., Uhrig M.L., Leskow F.C., Spagnuolo C.C. (2015). Recognition of saccharides in the NIR region with a novel fluorogenic boronolectin: In vitro and live cell labeling. Chem. Commun..

[40] Liu Y., Zhu J., Xu Y., Qin Y., Jiang D. (2015). Boronic Acid Functionalized Aza-Bodipy (azaBDPBA) based Fluorescence Optodes for the Analysis of Glucose in Whole Blood. ACS Appl. Mater. Interfaces.

[41] Itsuno S., Ito K., Hirao A., Nakahama S. (1983). Asymmetric reduction of aromatic ketones with the reagent prepared from (S)-(–)-2-amino-3-methyl-1,1-diphenylbutan-1-ol and borane. J. Chem. Soc. Chem. Commun..

[42] Brown H.C., Murray L.T. (1984). Molecular addition compounds. 9. Effect of structure on the reactivities of representative borane-amine complexes in typical reactions such as hydrolysis, hydroboration, and reduction. Inorg. Chem..

[43] Myers A.G., Yang B.H., Kopecky D.J. (1996). Lithium amidotrihydroborate, a powerful new reductant. Transformation of tertiary amides to primary alcohols. Tetrahedron Lett..

[44] Chaudhuri D., Kumar A., Rudra I., Sarma D.D. (2001). Synthesis and Spectroscopic Characterization of Highly Conducting BF_3_-Doped Polyaniline. Adv. Mater..

[45] Roberts M.F., Jenekhe S.A. (1993). Lewis acid coordination complexes of polymers. 1. Boron chloride, aluminum chloride and gallium chloride complexes of poly(p-phenylenebenzobisthiazole). Chem. Mater..

[46] Yamaguchi S., Akiyama S., Tamao K. (2002). A new approach to photophysical properties control of main group element π-electron compounds based on the coordination number change. J. Organomet. Chem..

[47] Qin Y., Pagba C., Piotrowiak P., Jäkle F. (2004). Luminescent Organoboron Quinolate Polymers. J. Am. Chem. Soc..

[48] Zhang L., Lin Y., Wang J., Yao W., Wu W., Jiang X. (2011). A Facile Strategy for Constructing Boron-Rich Polymer Nanoparticles via a Boronic Acid-Related Reaction. Macromol. Rapid Commun..

[49] Nomoto T., Yao Y., Inoue Y., Suzuki M., Kanamori K., Takemoto H., Matsui M., Tomoda K., Nishiyama N. (2021). Fructose-functionalized polymers to enhance therapeutic potential of p-boronophenylalanine for neutron capture therapy. J. Control. Release.

[50] Novoa S., Paquette J.A., Barbon S.M., Maar R.R., Gilroy J.B. (2016). Side-chain boron difluoride formazanate polymers via ring-opening metathesis polymerization. J. Mater. Chem. C.

[51] Wolfe P.S., Wagener K.B. (1999). Investigation of Organoboronates in Metathesis Polymerization. Macromolecules.

[52] Feng S., Zhang X., Li Y., Yu F., Dong Z., Sun Z., Chen Z.-R. (2025). Frustrated Lewis Pair-Mediated Catalyst Efficiency in ROMP: Mechanistic Insights from Boronic Ester-Functionalized Monomers. Macromolecules.

[53] Pawar G.M., Lalancette R.A., Bonder E.M., Sheridan J.B., Jäkle F. (2015). ROMP-Derived Pyridylborate Block Copolymers: Self-Assembly, pH-Responsive Properties, and Metal-Containing Nanostructures. Macromolecules.

[54] Eghbarieh N., Hanania N., Zamir A., Nassir M., Stein T., Masarwa A. (2021). Stereoselective Diels–Alder Reactions of gem-Diborylalkenes: Toward the Synthesis of gem-Diboron-Based Polymers. J. Am. Chem. Soc..

[55] Majchrzak M., Garbarek J., Eissa A.M. (2026). Design, Synthesis and ROMP of Novel Exo-Norbornene Silyl Ethers for Functional Polymer Applications. Materials.

[56] Garbarek J., Majchrzak M. (2026). Selective Ruthenium-Catalysed Functionalisation Reactions and ROMP of exo-Norbornene-Based Organosilicon Boronic Esters. Catalysts.

[57] Garbarek J., Majchrzak M., Wałęsa-Chorab M., Kubicki M. (2025). Synthesis and Electrochemical Properties of Well-Defined Norbornene-B-ferrocene-dioxaborolane Hybrid Polymeric Material. Inorg. Chem..

[58] Rule J.D., Moore J.S. (2002). ROMP Reactivity of endo- and exo-Dicyclopentadiene. Macromolecules.

[59] Suresh V.M., Bandyopadhyay A., Roy S., Pati S.K., Maji T.K. (2015). Highly Luminescent Microporous Organic Polymer with Lewis Acidic Boron Sites on the Pore Surface: Ratiometric Sensing and Capture of F^−^ Ions. Chem. Eur. J..

[60] Li Z., Li H., Xia H., Ding X., Luo X., Liu X., Mu Y. (2015). Triarylboron-Linked Conjugated Microporous Polymers: Sensing and Removal of Fluoride Ions. Chem.—A Eur. J..

[61] Zhao W., Zhuang X., Wu D., Zhang F., Gehrig D., Laquai F., Feng X. (2013). Boron-π-Nitrogen-Based Conjugated Porous Polymers with Multi-Functions. J. Mater. Chem. A.

[62] Parab K., Venkatasubbaiah K., Jäkle F. (2006). Luminescent Triarylborane-Functionalized Polystyrene: Synthesis, Photophysical Characterization, and Anion-Binding Studies. J. Am. Chem. Soc..

[63] Aota N., Nakagawa R., de Sousa L.E., Tohnai N., Minakata S., de Silva P., Takeda Y. (2024). Anion-Responsive Colorimetric and Fluorometric Red-Shift in Triarylborane Derivatives: Dual Role of Phenazaborine as Lewis Acid and Electron Donor. Angew. Chem. Int. Ed. Engl..

[64] Cromwell O.R., Chung J., Guan Z. (2015). Malleable and Self-Healing Covalent Polymer Networks through Tunable Dynamic Boronic Ester Bonds. J. Am. Chem. Soc..

[65] Kim C., Ejima H., Yoshie N. (2018). Dynamic Boronic Ester-Based Materials Accelerated by B-N Coordination. J. Mater. Chem. A.

[66] Song K., Ye W., Gao X., Fang H., Zhang Y., Zhang Q., Li X., Yang S., Wei H., Ding Y. (2020). Synergy between dynamic covalent boronic ester and boron–nitrogen coordination: Strategy for self-healing polyurethane elastomers at room temperature with unprecedented mechanical properties. Mater. Horiz..

[67] Wong K.T., Chien Y.Y., Liao Y.L., Lin C.C., Chou M.Y., Leung M.K. (2002). Efficient and Convenient Nonaqueous Workup Procedure for the Preparation of Arylboronic Esters. J. Org. Chem..

[68] Alver Ö. (2011). DFT, FT-Raman, FT-IR, solution and solid state NMR studies of 2,4-dimethoxyphenylboronic acid. C. R. Chim..

[69] Oh S.W., Weiss J.W.E., Kerneghan P.A., Korobkov I., Maly K.E., Bryce D.L. (2012). Solid-state ^11^B and ^13^C NMR, IR, and X-ray crystallographic characterization of selected arylboronic acids and their catechol cyclic esters. Magn. Reson. Chem..

[70] Wrackmeyer B. (2015). Organoboranes and tetraorganoborates studied by ^11^B and ^13^C NMR spectroscopy and DFT calculations. Z. Naturforsch. B.

[71] Costa L.C., Shieh P., Zafar H., Thiabaud G., Bobylev E.O., Jasanoff A., Johnson J.A. (2023). Hydrogen Peroxide-Triggered Disassembly of Boronic Ester-Cross-Linked Brush-Arm Star Polymers. ACS Macro Lett..

[72] Deposition Numbers 2283412 (for M1), 2283411 (for M2), 2283408 (for M3), 2283406 (for M4), 2283407 (for M5), 2283410 (for M6), 2283409 (for M7) Contain the Supplementary Crystallographic Data for This Paper. These Data Are Provided Free of Charge by the Joint Cambridge Crystallographic Data Centre Service. https://www.ccdc.cam.ac.uk/structures/.

[73] Ritter T., Hejl A., Wenzel A.G., Funk T.W., Grubbs R.H. (2006). A Standard System of Characterization for Olefin Metathesis Catalysts. Organometallics.

[74] Wang L., Wang X., Feng S., Li L. (2024). Dynamic Boronic Ester Cross-Linked Polymers with Tunable Properties via Side-Group Engineering. Polymers.

[75] Zeng Y., Li J., Liu S., Yang B. (2021). Rosin-Based Epoxy Vitrimers with Dynamic Boronic Ester Bonds. Polymers.

[76] Luo Y.-R. (2007). Chemical Bond Energies. Comprehensive Handbook of Chemical Bond Energies.

[77] Höfle G., Steglich W., Vorbrüggen H. (1978). 4-Dialkylaminopyridines as Highly Active Acylation Catalysts. [New synthetic method (25)]. Angew. Chem. Int. Ed..

[78] Hansch C., Leo A., Taft R.W. (1991). A survey of Hammett substituent constants and resonance and field parameters. Chem. Rev..

[79] Deposition Number 2388368 (for C2) Contains the Supplementary Crystallographic Data for This Paper. These Data Are Provided Free of Charge by the Joint Cambridge Crystallographic Data Centre Service. https://www.ccdc.cam.ac.uk/structures/.

[80] (2019). Rigaku Oxford Diffraction CrysAlisPro Software System.

[81] Sheldrick G.M. (2015). SHELXT—Integrated space-group and crystal structure determination. Acta Crystallogr. Sect. A.

[82] Sheldrick G.M. (2015). Crystal structure refinement with SHELXL. Acta Crystallogr. Sect. C Struct. Chem..

[83] National Research Council (US) Committee on Prudent Practices in the Laboratory (2011). Prudent Practices in the Laboratory: Handling and Management of Chemical Hazards.

[84] Choi T.L., Grubbs R.H. (2003). Controlled living ring-opening-metathesis polymerization by a fast-initiating ruthenium catalyst. Angew. Chem. Int. Ed..

[85] Bielawski C.W., Benitez D., Morita T., Grubbs R.H. (2001). Synthesis of End-Functionalized Poly(norbornene)s via Ring-Opening Metathesis Polymerization. Macromolecules.

[86] Katz T.J., Lee S.I., Acton N. (1976). Stereospecific Polymerizations of Cycloalkenes Induced by a Metal-carbene. Tetrahedron Lett..

[87] Schrock R.R., Feldman J., Cannizzo L.F., Grubbs R.H. (1987). Ring-opening Polymerization of Norbornene by a Living Tungsten Alkylidene Complex. Macromolecules.

