# Supplementary material for: Boron-Functionalised exo-Norbornene Monomers for ROMP: Toward Coordination-Active Poly(norbornene-B-arylene-dioxaborepine)s
Source: Molecules. 2026 Sep 8;31(18):3151. doi: 10.3390/molecules31183151 (PMC13609230; doi:10.3390/molecules31183151)
Supplement: Supplementary file 1 [file molecules-31-03151-s001.zip › molecules-4499887-supplementary V3.pdf]

Electronic Supplementary Information

Boron-functionalised *exo*-Norbornene Monomers for ROMP: Toward Coordination-active Poly(norbornene-B-arylene-dioxaborepine)s

Jerzy Garbarek [a], Mariusz Majchrzak [a*] and Maciej Kubicki [a]

[a] Faculty of Chemistry, Adam Mickiewicz University in Poznań, 8 Uniwersytetu Poznańskiego Str., 61-614 Poznań, Poland

*Corresponding authors: mariusz.majchrzak@amu.edu.pl

Table of contents

Experimental 2
- Materials 2
- General methods 2
- Preliminary studies – synthesis of model compounds 4
- Monomer procedures synthesis and analytic data 4
- Polymer synthesis procedures 8
- Coordination studies 9

NMR Spectra 10
- Preliminary studies 10
- Monomers 14
- Polymers 28
- Coordination studies 39

Gel permeation chromatography (GPC) analysis (blue line) 45

TGA analysis 51

X-Ray data 55

Experimental

Materials

The chemicals were obtained from following sources: toluene (99.8%), methylene chloride (DCM, 99.8%), n-hexane (99.5%), tetrahydrofuran (99.8%) were purchased from Chempur and P.O.Ch. Gliwice, 5-norbornene-2-*exo*-3-*exo*-dimethanol (>97%), ethylene glycol (>99%), 1,3-propanediol (>99%), phenylboronic acid (95%), pyrene-1-boronic acid (95%), 9-phenantracenylboronic acid (97%), 10-bromoanthracene-9-boronic acid (97%), 3,5-dibromophenylboronic acid (98%), *trans*-2-(4-methylphenyl)vinylboronic acid (97%), *trans*-2-(4-biphenyl)vinylboronic acid (95%), pyridine (99%) and 4-dimethylaminopyridine (99%), deuterated chloroform ($CDCl_3$) (>99.8%), Grubbs catalyst 1st generation $C_{43}H_{72}Cl_2P_2Ru$ [$RuCl_2$(=CHPh)(PCy_3)$_2$], Grubbs catalyst 2nd generation $C_{46}H_{65}Cl_2N_2PRu$ [$RuCl_2$(=CHPh)(PCy_3)($IMesH_2$)], from Aldrich, ethyl vinyl ether (99%) from Merck, magnesium sulfate ($MgSO_4$) from EuroChem. Acquired solvents - toluene, methylene chloride, tetrahydrofuran and n-hexane were stored over molecular sieves type A4. Methylene chloride used in polymerisation was kept in Schlenk's system in inert atmosphere over molecular sieves type A4.

General methods

^{1}H NMR (400 MHz) and ^{13}C NMR (105 MHz) spectra were recorded on Mercury plus 400MHz spectrometer. ^{11}B NMR spectra were acquired with Bruker Avance 600 MHz and Bruker Avance III 400 MHz spectrometers. In each method, $CDCl_3$ solution was used as residue solvent. Chemical shifts are reported in δ (ppm) with reference to the residue solvent used ($CDCl_3$) (1H δ H = 7.26 ppm, 13C δ C = 77.36 ppm).

Mass spectra (GC-MS) of monomers were obtained on Varian 3300 instrument equipped with capillary column type DB-1 ResteckTM, which was connected to Finnigan MAT 800 spectrometer with ion trap detector. Spectra were registered by using ITD Finnigan MAT program.

HR-MS measurement: High-resolution mass spectroscopic (HRMS) analyses were performed on an AMD-402 mass spectrometer which is a two-sector (B/E geometry) mass spectrometer manufactured by AMD Intectra GmbH (Germany). Two measurements were made of each monomer.

Elemental Analysis measurement: An elemental analysis was performed on a Vario Elementar Analyser Vario EL III Instrument (German) three times and then averaged to obtain the final results. The temperature range of analysis was 25–1100°C. The method of analysis involves catalytic combustion of the sample at 1200°C with gas separation on adsorption columns. Detection of material components was identified by thermal conductivity difference. The duration of a single analysis varied from 10 to 20 minutes with simultaneous determination of the elements: C, H, N, S. Three E. A. measurements were made from three weighed portions of each polymer.

Infrared spectroscopy (FT-IR) was performed on NICOLETTM iSTM50FT-IR spectrometer made by Thermo ScientificTM with addition of iSTM50ATR, in range between 4000 cm^{-1} to 400 cm^{-1}. For each spectrum 16 scans were registered.

Gel permeation chromatography (GPC) analyses were performed using an Agilent HPLC system equipped with RI detector (analysis conditions: mobile phase – dichloromethane (DCM); flow rate 0.80 mL/min; temperature 25-40°C; the volume of the injection 100 µL). Two 2X Plgel 5um mixed C were used for separation. The calibration of the OPTILAB T-reX (Wyatt) was carried out by p.a. grade toluene and normalization with a polystyrene standard of 30,000 g/mol. molar mass. The numerical average molecular weight (*Mn*), average molecular weight (*Mw*) and polydispersity index (PDI) were determined by polystyrene standards calibration curve (correlation coefficient: r^2=0.9993563). In determination of the molecular weight the Astra software should know the amount of the sample introduced for measurements to calculate *dn/dc* of polymer. We measured the dn/dc on line, when a sample is

passing through the columns assuming that the polymer does not interact with the columns giving eventually a good amount, equal to the value introduced to the software. ASTRA 6.2 software (Wyatt Technology Corporation) was used for data collecting and processing.

Crystallography_X-ray Measurement: X-ray diffraction data were collected at 100(1) K by the ω-scan technique on a Rigaku four circle Xcalibur diffractometer (Eos detector) with graphite-monochromatized Mo Kα radiation (λ = 0.71073 A). The data were corrected for Lorentz polarization and absorption effects.[80] Accurate unit-cell parameters were determined by least-squares fit of 1619 reflections of the highest intensity chosen from the whole experiment. The structures were solved with SHELXT76 [81] and refined with the full matrix least-squares procedure on F2 by SHELXL.[82]

X-Ray data: Crystallographic data for the structural analysis has been deposited with the Cambridge Crystallographic Data Centre, Nos. CCDC: 01_M1 - 2283412; 02_M2 - 2283411; 03_M3 - 2283408; 04_M4 - 2283406; 05_M5 - 2283412; 06_M6 - 2283411; 07_M7 - 2283408; 08_ - 2283406. Copies of this information may be obtained free of charge from: The Director, CCDC, 12 Union Road, Cambridge, CB2 1EZ, UK. Fax: +44(1223)336-033, e-mail:deposit@ccdc.cam.ac.uk, or www: www.ccdc.cam.ac.uk.

Thermal stability (TGA) was investigated by thermogravimetric Analyzer-TGA Q50 V20.10 Build 36. Samples of about 0.95 to 8.05 mg were heated in platinum pans at the rate of 10°C/min from 25°C to 1000°C under nitrogen atmosphere (90mL/min).

Notes

Note 1: These compounds must be handled using proper needle and syringe techniques with a vacuum Schlenk line. Contact with skin and the respiratory system should be strictly avoided. All manipulations with these compounds were performed following the reported safety procedures [83].

Note 2: Polymerisation reactions were carried out under dry argon atmosphere.

Note 3: Boron spectra were reprocessed using fitting tool in MestReNova software.

Preliminary studies – synthesis of model compounds

2-phenyl-1,3,2-dioxaborolane **(0a)** was obtained according to the preparation procedure of monomers described in the main article, using 0.15 g (2.42 mmol) of diol (p = 99%) and 0.307 g (2.52 mmol) of phenylboronic acid (p = 95%) in 10 mL of toluene, mixed for 2 h at *rt*, with 62% yield (0.221 g; 1.49 mmol) as colourless liquid.

Analytic data: ^{1}H NMR (400 MHz, $CDCl_3$): δ = 7.82 (dd, $J_{H,H}$ = 1.2, 8.0 Hz, 2H; o-C_6H_5 (A)), 7.51-7.37 (m, 3H; m-p-C_6H_5 (B)), 4.39 (s, 4H; -CH_2- (C)). ^{13}C NMR (105 MHz, $CDCl_3$): δ = 135.0, 131.6, 128.0, 66.2. MS (EI) [m/z (relat. Int. %)]: 147.9 ($M^{+•}$) (100), 90.9 (37), 104.9 (7).

2-phenyl-1,3,2-dioxaborinane **(0b)** was obtained according to the preparation procedure of monomers mentioned in the main article, using 0.3 g (3.94 mmol) of diol (p = 99%) and 0.5 g (4.10 mmol) of phenylboronic acid (p = 95%) in 10 mL of toluene, mixed for 2 h at *rt*, with 93% yield (0.594 g; 3.67 mmol) in form of colourless liquid and crystals.

Analytic data: ^{1}H NMR (600 MHz, $CDCl_3$): δ = 7.80 (dd, $J_{H,H}$ = 1.2 7.8 Hz, 2H; o-C_6H_5 (A)), 7.45-7.35 (m, 3H; m-p-C_6H_5 (B)), 4.18 (t, $J_{H,H}$ = 5.4 Hz, 4H; -CH_2- (C)), 2.06 (p, $J_{H,H}$ = 6.0 Hz, 2H; -CH_2- (D)). ^{13}C NMR (105 MHz, $CDCl_3$): δ = 133.7, 130.7, 127.7, 62.1, 27.6. ^{11}B NMR (128 MHz, $CDCl_3$): δ = 27.2. MS (EI) [m/z (relat. Int. %)]: 104.1 (100), 162.1 ($M^{+•}$) (98.1), 77.1 (27.4), 91.1 (25.9), 51.1 (12.3).

Monomer procedures synthesis and analytic data

5-norbornene-2-exo,3-exo-dimethyl-[(phenyl)-dioxaborepine] **(M1)** was obtained according to the preparation procedure described in the main article, using 0.3 g (1.95 mmol) of 5-norbornene-2-*exo*-3-*exo*-dimethanol (p = 97%) and 0.243 g (1.99 mmol) of phenylboronic acid (p = 95%) in 20 mL of toluene, mixed for 3 h at *rt*, with 99% yield (0.462 g; 1.92 mmol) as a white solid.

Analytic data: ^{1}H NMR (400 MHz, $CDCl_3$): δ = 7.76 (dd, $J_{H,H}$ = 1.6, 8.0Hz, 2H; o-C_6H_5 (A)), 7.63-7.29 (m, 3H; m-p-C_6H_5 (B)), 6.22 (t, $J_{H,H}$ = 1.6 Hz, 2H; -CH=CH- (C)), 4.34 (t, $J_{H,H}$ = 11.6 Hz, 2H; -CH_2- (D)), 4.11 (dd, $J_{H,H}$ = 3.6, 11.6 Hz, 2H; -CH_2- (E)), 2.57 (p, $J_{H,H}$ = 1.6 Hz, 2H; -CH- (F)), 2.15-2.08 (m, 2H; -CH- (G)), 1.52 (dt, $J_{H,H}$ = 1.6, 8.8 Hz, 1H; -CH_2- (H)), 1.33 (dt, $J_{H,H}$ = 1.6, 8.8 Hz, 1H; -CH_2- (H')). ^{13}C NMR (105 MHz, $CDCl_3$): δ = 137.4, 135.8, 134.2, 130.4, 128.1, 127.6, 67.3, 45.1, 43.4, 42.9. ^{11}B NMR (128 MHz, $CDCl_3$): δ = 26.7. MS (EI) [m/z (relat. Int. %)]: 240.0 ($M^{+•}$) (100), 239.2 (34), 173.0 (26), 159.1 (63), 119.1 (70), 105.1 (17), 90.9 (24), 66.1 (73). HRMS (M/Z) calcd. for $C_{15}H_{17}BO_2$: 240.1322; found 240.1321.

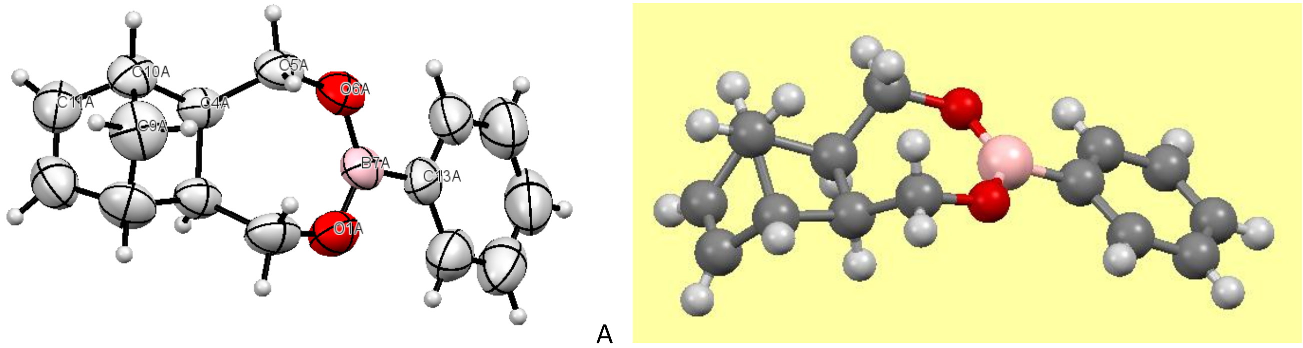

A B

Figure S1. X-Ray structure of M1: a) The typical X-ray image; b) The lateral X-Ray projection shows perfect *exo-*,*exo-* arrangement of the norborene part to the boronate ester part.

5-norbornene-2-exo,3-exo-dimethyl-[(pyrene-1)-dioxaborepine] **(M2)** was obtained according to the preparation procedure described in the main article, using 0.3 g (1.95 mmol) of 5-norbornene-2-*exo*-3-*exo*-dimethanol (p = 97%) and 0.490 g (1.99 mmol) of pyrene-1-boronic acid (p = 95%) in 20 mL of toluene, mixed for 7 h at 75°C, with 97% yield (0.688 g; 1.89 mmol) as a beige solid.

Analytic data: ^{1}H NMR (400 MHz, $CDCl_3$): δ = 9.03 (d, $J_{H,H}$ = 9.6 Hz, 1H; (A)), 8.46 (d, $J_{H,H}$ = 8.0 Hz, 1H; (B)), 8.20-7.97 (m, 7H; (C)), 6.27 (t, $J_{H,H}$ = 1.6 Hz, 2H; -CH=CH- (D)), 4.55 (t, $J_{H,H}$ = 11.6 Hz, 2H; -CH_2- (E)), 4.32 (dd, $J_{H,H}$ = 4.0, 11.6 Hz, 2H; -CH_2- (F)), 2.66 (p, $J_{H,H}$ = 1.6 Hz, 2H; -CH- (G)), 2.34-2.27 (m, 2H; -CH- (H)), 1.61 (dt, $J_{H,H}$ = 1.6, 8.8 Hz, 1H; -CH_2- (I)),

1.40 (dt, $J_{H,H}$ = 1.6, 8.8 Hz, 1H; -C*H*$_2$- (I’)). ^{13}C NMR (105 MHz, $CDCl_3$): δ = 137.4, 135.7, 133.2, 132.8, 131.4, 130.9, 128.3, 128.1, 127.7, 127.2, 125.6, 125.1, 125.0, 124.97, 124.2, 67.6, 45.1, 43.4, 43.1. ^{11}B NMR (128 MHz, $CDCl_3$): δ = 27.8. MS (EI) [m/z (relat. Int. %)]: 364.9 ($M^{+\bullet}$) (100), 298.9 (62), 228.8 (13), 206.7 (11), 66.4 (9). HRMS (M/Z) calcd. for $C_{25}H_{21}BO_2$: 364.1635; found 364.1633.

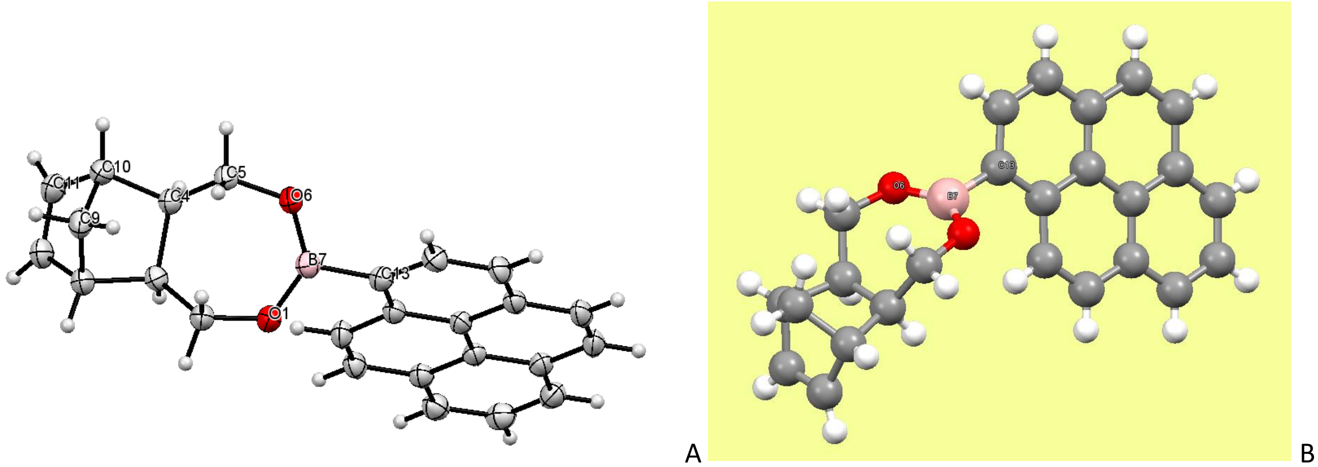

Figure S2. X-Ray structure of M2: a) The typical X-ray image; b) The lateral X-Ray projection shows perfect *exo-*,*exo-*arrangement of the norborene part to the boronate ester part.

5-norbornene-2-exo,3-exo-dimethyl-[(9-phenantracenyl)-dioxaborepine] **(M3)** was obtained according to the preparation procedure described in the main article, using 0.3 g (1.95 mmol) of 5-norbornene-2-*exo*-3-*exo*-dimethanol (p = 97%) and 0.433 g (1.95 mmol) of 9-phenantracenylboronic acid (p = 97%) in 20 mL of toluene, mixed for 6 h at 80°C, with 97% yield (0.642 g; 1.89 mmol) as a beige solid.

Analytic data: ^{1}H NMR (400 MHz, $CDCl_3$): δ = 8.74-8.65 (m, 3H; (A)), 8.25 (s, 1H; (B)), 7.89 (dd, $J_{H,H}$ = 1.6, 8.0 Hz, 1H; (C)), 7.66-7.54 (m, 4H; (D)), 6.26 (t, $J_{H,H}$ = 1.6 Hz, 2H; -C*H*=C*H*- (E)), 4.50 (t, $J_{H,H}$ = 12.0 Hz, 2H; -C*H*$_2$- (F)), 4.26 (dd, $J_{H,H}$ = 4.0, 11.6 Hz, 2H; -C*H*$_2$- (G)), 2.64 (p, $J_{H,H}$ = 1.6 Hz, 2H; -C*H*- (H)), 2.29-2.25 (m, 2H; -C*H*- (I)), 1.59 (dt, $J_{H,H}$ = 1.6, 8.8 Hz, 1H; -C*H*$_2$- (J)), 1.39 (dt, $J_{H,H}$ = 1.6, 8.8 Hz, 1H; -C*H*$_2$- (J’)). ^{13}C NMR (105 MHz, $CDCl_3$): δ = 137.4, 136.3, 134.6, 131.6, 131.3, 130.2, 129.3, 129.2, 127.3, 126.40, 126.37, 125.9, 122.8, 122.5, 67.6, 45.1, 43.4, 43.0. ^{11}B NMR (128 MHz, $CDCl_3$): δ = 27.8. MS (EI) [m/z (relat. Int. %)]: 340.5 ($M^{+\bullet}$) (100), 274.8 (15), 66.3 (4). HRMS (M/Z) calcd. for $C_{23}H_{21}BO_2$: 340.1635; found 340.1634.

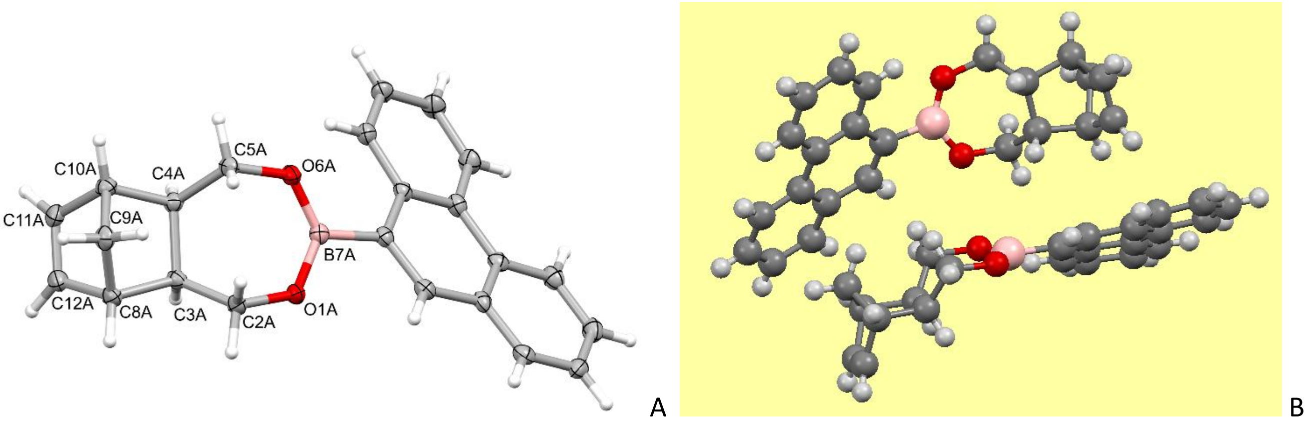

Figure S3. X-Ray structure of M3: a) The typical X-ray image; b) The lateral X-Ray projection shows perfect *exo-*,*exo-*arrangement of the norborene part to the boronate ester part.

5-norbornene-2-exo,3-exo-dimethyl-[(10-bromoanthracene-9)-dioxaborepine] **(M4)** was obtained according to the preparation procedure described in the main article, using 0.3 g (1.95 mmol) of 5-norbornene-2-*exo*-3-*exo*-dimethanol (p = 97%) and 0.581 g (1.93 mmol) of 10-bromoanthracene-9-boronic acid (p = 98%) in 20 mL of toluene, mixed for 6 h at 80°C, with 97% yield (0.8 g; 1.91 mmol) as a brick-red solid.

Analytic data: ^{1}H NMR (400 MHz, $CDCl_3$): δ = 8.52 (qd, $J_{H,H}$ = 0.8, 8.8 Hz, 2H; (A)), 8.07 (qd, $J_{H,H}$ = 0.8, 8.4 Hz, 2H; (B)), 7.57-7.46 (m, 4H; (C)), 6.34 (t, $J_{H,H}$ = 2.0 Hz, 2H; -C*H*=C*H*- (D)), 4.65 (t, $J_{H,H}$ = 12.0 Hz, 2H; -C*H*$_2$- (E)), 4.24 (dd, $J_{H,H}$ = 4.0, 12.0 Hz, 2H; -C*H*$_2$- (F)), 2.69 (p, $J_{H,H}$ = 1.6 Hz, 2H; -C*H*- (G)), 2.48-2.44 (m, 2H; -C*H*- (H)), 1.64 (dt, $J_{H,H}$ = 1.6, 9.2 Hz, 1H; -

CH_2- (I)), 1.45 (dt, $J_{H,H}$ = 1.6, 9.2 Hz, 1H; -CH_2- (I')). ^{13}C NMR (105 MHz, $CDCl_3$): δ = 137.5, 134.5, 134.2, 130.3, 129.1, 128.3, 127.4, 126.8, 125.5, 123.8, 68.1, 45.2, 43.41, 43.37. ^{11}B NMR (128 MHz, $CDCl_3$): δ = 29.7. MS (EI) [m/z (relat. Int. %)]: 420.6 (M+•) (100), 355.2 (14), 353.3 (23), 282.2 (10), 202.3 (5), 66.5 (10). HRMS (M/Z) calcd. for $C_{23}H_{20}BBrO_2$: 418.0740; found 418.0739.

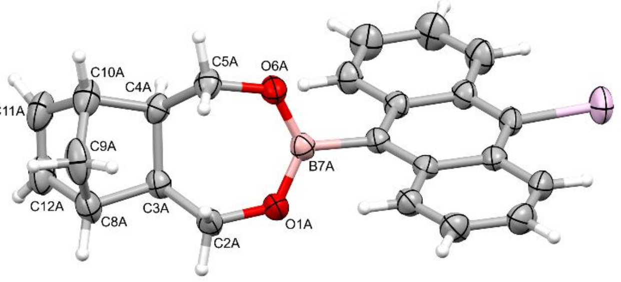

A
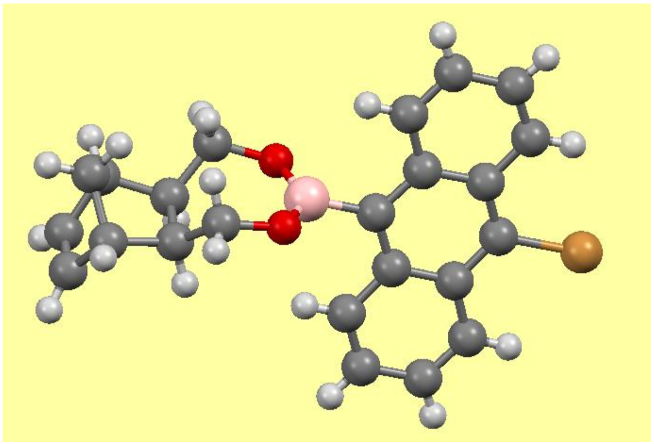
B

Figure S4. X-Ray structure of M4: a) The typical X-ray image; b) The lateral X-Ray projection shows perfect exo-,exo-arrangement of the norborene part to the boronate ester part.

5-norbornene-2-exo,3-exo-dimethyl-[(3,5-dibromophenyl)-dioxaborepine] **(M5)** was obtained according to the preparation procedure described in the main article, using 0.3 g (1.95 mmol) of 5-norbornene-2-*exo*-3-*exo*-dimethanol (p = 97%) and 0.54 g (1.93 mmol) of 3,5-dibromophenylboronic acid (p = 98%) in 20 mL of toluene, mixed for 4 h at 80°C, with 98% yield (0.76 g; 1.91 mmol) as a beige-white solid.

Analytic data: ^{1}H NMR (400 MHz, $CDCl_3$): δ = 7.78 (d, $J_{H,H}$ = 1.6 Hz, 2H; o-C_6H_5 (A)), 7.66 (t, $J_{H,H}$ = 2.0 Hz, 1H; p-C_6H_5 (B)), 6.22 (t, $J_{H,H}$ = 2.0 Hz, 2H; -C*H*=C*H*- (C)), 4.31 (t, $J_{H,H}$ = 12.0 Hz, 2H; -CH_2- (D)), 4.10 (dd, $J_{H,H}$ = 4.0, 11.6 Hz, 2H; -CH_2- (E)), 2.57 (p, $J_{H,H}$ = 1.6 Hz, 2H; -C*H*- (F)), 2.10-2.06 (m, 2H; -C*H*- (G)), 1.50 (dt, $J_{H,H}$ = 1.6, 9.2 Hz, 1H; -CH_2- (H)), 1.34 (dt, $J_{H,H}$ = 1.6, 9.2 Hz, 1H; -CH_2- (H')). ^{13}C NMR (105 MHz, $CDCl_3$): δ = 137.4, 135.65, 135.59, 122.7, 67.5, 45.0, 43.4, 42.9. ^{11}B NMR (128 MHz, $CDCl_3$): δ = 25.8. MS (EI) [m/z (relat. Int. %)]: 66.2 ($M^{+•}$) (100), 400.5 (42), 399.5 (14), 398.6 (18), 92.3 (19), 79.2 (15). HRMS (M/Z) calcd. for $C_{15}H_{15}BBr_2O_2$: 395.9532; found 395.9531.

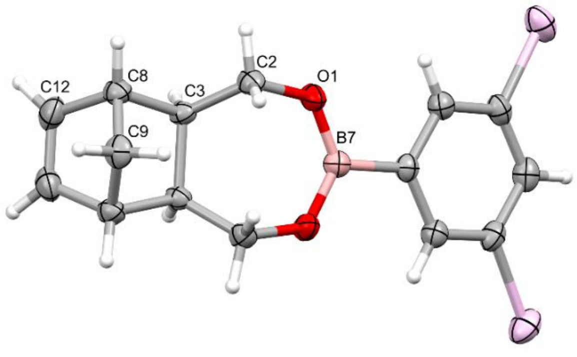

A
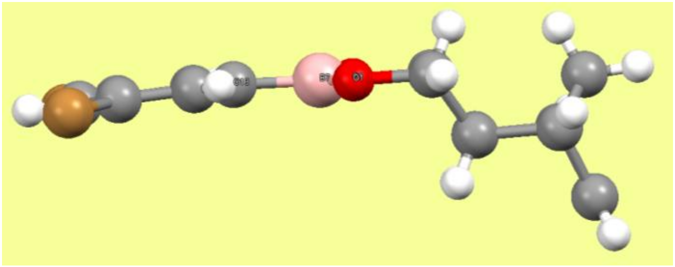
B

Figure S5. X-Ray structure of M5: a) The typical X-ray image; b) The lateral X-Ray projection shows perfect *exo-,exo-*arrangement of the norborene part to the boronate ester part.

5-norbornene-2-exo,3-exo-dimethyl-[(trans-2-(4-methylphenyl)vinyl)-dioxaborepine] **(M6)** was obtained according to the preparation procedure described in the main article, using 0.3 g (1.95 mmol) of 5-norbornene-2-*exo*-3-*exo*-dimethanol (p = 97%) and 0.316 g (1.95 mmol) of *trans*-2-(4-methylphenyl)vinylboronic acid (p = 97%) in 20 mL of toluene, mixed for 1 h at 40°C, with 95% yield (0.518 g; 1.85 mmol) as a white solid.

Analytic data: ^{1}H NMR (400 MHz, $CDCl_3$): δ = 7.36 (d, $J_{H,H}$ = 8.4 Hz, 2H; (A),), 7.22 (d, $J_{H,H}$ = 18.0 Hz, 1H; -C*H*=C*<u>H</u>*- (B)), 7.12 (d, $J_{H,H}$ = 8.0 Hz, 2H; (C)), 6.21 (t, $J_{H,H}$ = 2.0 Hz, 2H; -C*H*=C*H*- (D)), 5.98 (d, $J_{H,H}$ = 18.0 Hz, 1H; -C*<u>H</u>*=C*H*- (E)), 4.25 (t, $J_{H,H}$ = 12.0 Hz, 2H; -CH_2- (F)), 4.02 (dd, $J_{H,H}$ = 4.0, 12.0 Hz, 2H; -CH_2- (G)), 2.54 (p, $J_{H,H}$ = 1.6 Hz, 2H; -C*H*- (H)), 2.33 (s, 3H; -CH_3 (I)), 2.09-2.06 (m, 2H; -C*H*- (J)), 1.48 (dt, $J_{H,H}$ = 1.6, 9.2 Hz, 1H; -CH_2- (K)), 1.31 (dt, $J_{H,H}$ = 1.6, 9.2 Hz, 1H; -CH_2- (K')). ^{13}C NMR (105 MHz, $CDCl_3$): δ = 146.8, 138.4, 137.4, 135.4, 134.7, 129.3, 127.0, 67.2, 45.0, 45.3, 42.9, 21.4. ^{11}B NMR (128 MHz, $CDCl_3$): δ = 26.7. MS (EI) [m/z (relat. Int. %)]: 280.5 (100), 279.8 (36), 234.8 (50), 170.4 (16), 155.5 (7), 119.3 (19), 105.2 (9). HRMS (M/Z) calcd. for $C_{18}H_{21}BO_2$: 280.1635; found 280.1635.

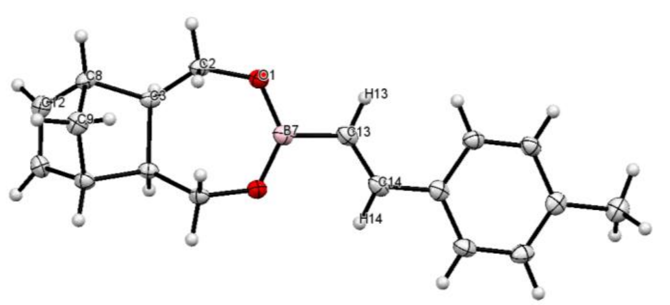

A

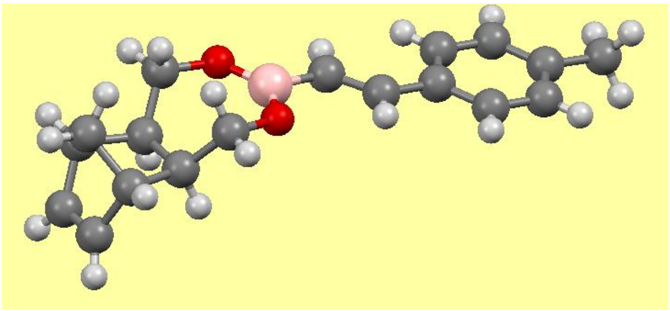

B

Figure S6. X-Ray structure of M6: a) The typical X-ray image; b) The lateral X-Ray projection shows perfect *exo*-,*exo*-arrangement of the norborene part to the boronate ester part.

5-norbornene-2-exo,3-exo-dimethyl-[(trans-2-(4-biphenyl)vinyl)-dioxaborepine] **(M7)** was obtained according to the preparation procedure described in the main article, using 0.3 g (1.95 mmol) of 5-norbornene-2-*exo*-3-*exo*-dimethanol (p = 97%) and 0.446 g (1.99 mmol) of *trans*-2-(4-biphenyl)vinylboronic acid (p = 95%) in 20 mL of toluene, mixed for 2 h at 40°C, with 54% yield (0.36 g; 1.05 mmol) as a white solid. The product demanded purification by washing the resulting solid with n-hexane.

Analytic data: ^{1}H NMR (400 MHz, $CDCl_3$): δ = 7.60 (dd, $J_{H,H}$ = 1.2, 8.4 Hz, 2H; (A)), 7.55 (s, 2H; (B)), 7.54 (s, 2H; (C)), 7.45-7.41 (m, 2H; (D)), 7.36-7.31 (m, 1H; (E)), 7.29 (d, $J_{H,H}$ = 18.0 Hz, 1H; -C*H*=C$\underline{H}$- (F)), 6.22 (t, $J_{H,H}$ = 2.0 Hz, 2H; -C*H*=C*H*- (G)), 6.09 (d, $J_{H,H}$ = 18.0 Hz, 1H; -C$\underline{H}$=C*H*- (H)), 4.27 (t, $J_{H,H}$ = 12.0 Hz, 2H; -CH_2- (I)), 4.04 (dd, $J_{H,H}$ = 3.6, 11.6 Hz, 2H; -CH_2- (J)), 2.56 (p, $J_{H,H}$ = 1.6 Hz, 2H; -C*H*- (K)), 2.12-2.08 (m, 2H; -C*H*- (L)), 1.50 (dt, $J_{H,H}$ = 1.6, 9.2 Hz, 1H; -CH_2- (M)), 1.32 (dt, $J_{H,H}$ = 1.6, 9.2 Hz, 1H; -CH_2- (M’)). ^{13}C NMR (105 MHz, $CDCl_3$): δ = 146.3, 141.2, 140.8, 137.4, 137.1, 128.9, 127.53, 127.46, 127.3, 127.1, 67.2, 45.1, 43.3, 42.9. ^{11}B NMR (128 MHz, $CDCl_3$): δ = 26.3. MS (EI) [m/z (relat. Int. %)]: 342.2 ($M^{+\bullet}$) (100), 276.4 (22), 66.2 (8). HRMS (M/Z) calcd. for $C_{23}H_{23}BO_2$: 342.1791; found 342.1790.

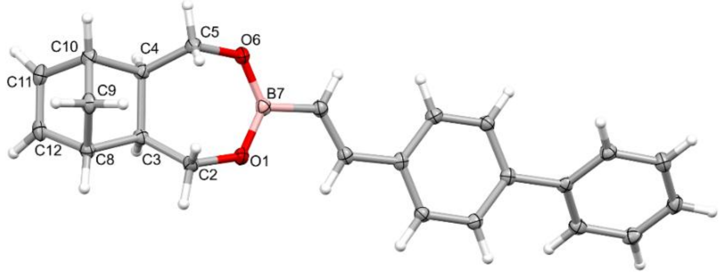

A

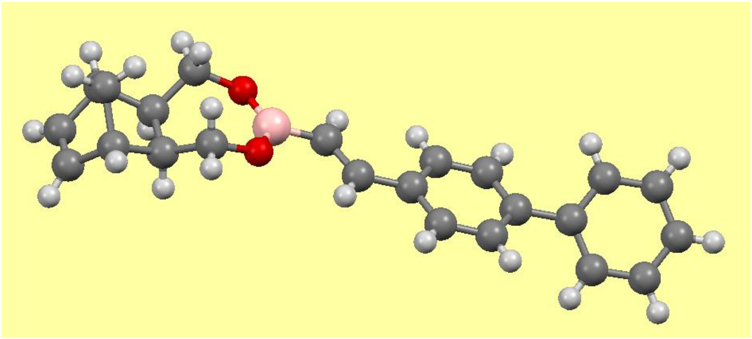

B

Figure S7. X-Ray structure of M7: a) The typical X-ray image; b) The lateral X-Ray projection shows perfect *exo*-,*exo*-arrangement of the norborene part to the boronate ester part.

Polymer synthesis procedures

***Poly(5-norbornene-2-exo,3-exo-dimethyl-[(phenyl)-dioxaborepine])s – G1* (P1/P1a)** was obtained according to the preparation procedure described in the main article, using 0.15 g of monomer **M1**, 0.02 M solution of 1st generation Grubbs catalyst (312 µL, 6.25x10^{-6} mol), in 11.1 mL of dichloromethane (C = 0.056 mol/L), mixed for 2.5 h at *rt*, with 97% yield (0.145 g) as a white solid. Additional tests were carried out with polymerisation lasting 21 h and 5 min.

Analytic data: ^{1}H NMR (400 MHz, $CDCl_3$): δ = 7.79 (br d, 2H; o-C_6H_5 (A)), 7.34 (br d, 3H; m-p-C_6H_5 (B)), 5.41 (br d, 2H; -C*H*=C*H*- (C)), 4.22 (br s, 4H; -C*H*$_2$- (D)), 2.29 (br s, 2H; -C*H*- (E)), 1.91 (br s, 2H; -C*H*- (F)), 1.35 (br s, 2H; -C*H*$_2$- (G)).^{13}C NMR (105 MHz, $CDCl_3$): δ = 134.3, 133.8, 133.1, 130.6, 127.6, 64.5, 48.9, 45.1, 40.7 ^{11}B NMR (128 MHz, $CDCl_3$): δ = 28.9. Elemental Analyses calcd. for $C_{16}H_{18}BO_2$: C 75.92, H 7.17; found C 75.90, H 7.16.

***Poly(5-norbornene-2-exo,3-exo-dimethyl-[(phenyl)-dioxaborepine])s – G2* (P1b)** was obtained according to the preparation procedure described in the main article, using 0.04 g of monomer **M1**, 0.02 M solution of 2nd generation Grubbs catalyst (83 µL, 1.67x10^{-6} mol), in 2.5 mL of dichloromethane (C = 0.067 mol/L), mixed for 2.5 h at *rt*. The resulting polymer was received as white solid with 65% yield (0.026 g).

Analytic data: ^{1}H NMR (400 MHz, $CDCl_3$): δ = 7.80 (br s, 2H; o-C_6H_5 (A)), 7.33 (br d, 3H; m-p-C_6H_5 (B)), 5.30 (br d, 2H; -C*H*=C*H*- (C)), 4.19 (br d, 4H; -C*H*$_2$- (D)), 2.54-2.27 (br d, 2H; -C*H*- (E)), 1.89 (br s, 2H; -C*H*- (F)), 1.33 (br s, 2H; -C*H*$_2$- (G)).

***Poly(5-norbornene-2-exo,3-exo-dimethyl-[(9-phenantracenyl)-dioxaborepine])s* (P3)** was obtained according to the preparation procedure described in the main article, using 0.125 g of monomer **M3**, 0.02 M solution of 1st generation Grubbs catalyst (184 µL, 3.67x10^{-6} mol), in 8.5 mL of dichloromethane (C = 0.043 mol/L), mixed for 3 h at *rt*, with 97% yield (0.121 g) as a white solid.

Analytic data: ^{1}H NMR (400 MHz, $CDCl_3$): δ = 8.78-8.64 (br d, 3H; (A)), 8.32 (br s, 1H; (B)), 7.89 (br s, 1H; (C)), 7.59 (br d, 4H; (D)), 5.40-5.28 (br d, 2H; -C*H*=C*H*- (E)), 4.32 (br s, 4H; -C*H*$_2$- (F)), 2.33 (br s, 2H; -C*H*- (G)), 1.89 (br s, 2H; -C*H*- (H)), 1.33 (br s, 2H; -C*H*$_2$- (I)). ^{13}C NMR (105 MHz, $CDCl_3$): δ = 136.7, 134.7, 133.2, 131.7, 131.3, 130.3, 129.3, 127.4, 126.56, 126.48, 126.0, 122.9, 122.6, 64.8, 48.9, 45.1, 40.8. ^{11}B NMR (128 MHz, $CDCl_3$): δ = 30.0. Elemental Analyses calcd. for $C_{24}H_{22}BO_2$: C 81.60, H 6.28; found C 81.59, H 6.27.

***Poly(5-norbornene-2-exo,3-exo-dimethyl-[(3,5-dibromophenyl)-dioxaborepine])s* (P5)** was obtained according to the preparation procedure described in the main article, using 0.125 g of monomer **M5**, 0.02 M solution of 1st generation Grubbs catalyst (157 µL, 3.141x10^{-6} mol), in 10 mL of dichloromethane (C = 0.031 mol/L), mixed for 2.5 h at *rt*, with 98% yield (0.123 g) as a white solid.

Analytic data: ^{1}H NMR (400 MHz, $CDCl_3$): δ = 7.81 (br s, 2H; o-C_6H_5 (A)), 7.67 (br s, 1H; p-C_6H_5 (B)), 5.40-5.30 (br d, 2H; -C*H*=C*H*- (C)), 4.20 (br s, 4H; -C*H*$_2$- (D)), 2.27 (br s, 2H; -C*H*- (E)), 1.93 (br s, 2H; -C*H*- (F)), 1.35 (br s, 2H; -C*H*$_2$- (G)). ^{13}C NMR (105 MHz, $CDCl_3$): δ = 135.8, 133.0, 135.5, 122.8, 64.7, 48.7, 45.1, 29.9. ^{11}B NMR (128 MHz, $CDCl_3$): δ = 27.2. Elemental Analyses calcd. for $C_{16}H_{16}BBr_2O_2$: C 46.77, H 3.92; found C 46.75, H 3.91.

***Poly(5-norbornene-2-exo,3-exo-dimethyl-[(trans-2-(4-methylphenyl)vinyl)-dioxaborepine])s* (P6)** was obtained according to the preparation described in the main article, using 0.1715 g of monomer **M6**, 0.02 M solution of 1st generation Grubbs catalyst (306 µL, 6.121x10^{-6} mol), in 10 mL of dichloromethane (C = 0.061 mol/L), mixed for 1 h at *rt*, with 90% yield (0.155 g) as a white solid.

Analytic data: ^{1}H NMR (400 MHz, $CDCl_3$): δ = 7.38 (br s, 2H; (A)), 7.27-7.23 (br s, 1H; -C*H*=C*H̲*- (B)), 7.12 (br d, 2H; (C)), 6.02-5.98 (br d, 1H; -C*H̲*=C*H*- (D)), 5.39-5.29 (br d, 2H; -C*H*=C*H*- (E)), 4.14-4.08 (br d, 4H; -C*H*$_2$- (F)), 2.33 (s, 3H; -C*H*$_3$ (G)), 2.25 (br s, 2H; -C*H*- (H)), 1.90 (br s, 2H; -C*H*- (I)), 1.32 (br s, 2H; -C*H*$_2$- (J)). ^{13}C NMR (105 MHz, $CDCl_3$): δ = 147.1, 138.5, 135.3, 133.1, 129.4, 127.1, 121.2, 64.3, 48.8, 45.1, 40.8, 21.4. ^{11}B NMR (128 MHz, $CDCl_3$): δ = 29.1. Elemental Analyses calcd. for $C_{19}H_{22}BO_2$: C 77.84, H 7.56; found C 77.81, H 7.55.

***Poly(5-norbornene-2-exo,3-exo-dimethyl-[(trans-2-(4-biphenyl)vinyl)-dioxaborepine])s* (P7)** was obtained according to the preparation procedure described in the main article, using 0.04 g of monomer M7, 0.02 M solution of 1st generation Grubbs catalyst (58 µL, 1.169x10^{-6} mol), in 2.5 mL of dichloromethane (C = 0.047 mol/L), mixed for 1 h at *rt*, with 88% yield (0.0352 g) as a white solid.

Analytic data: ^{1}H NMR (400 MHz, $CDCl_3$): δ = 7.56 (br s, 6H; (A)), 7.41 (br s, 2H; (B)), 7.32 (br s, 2H; Ar and -CH=CH̲- (C,D)), 6.13-6.08 (br d, 1H; -C*H̲*=C*H*- (E)), 5.42-5.30 (br d, 2H; -C*H*=C*H*- (F)), 4.17-4.10 (br d, 4H; -C*H*$_2$- (G)), 2.27 (br s,

2H; -C*H*- (H)), 1.93 (br s, 2H; -C*H*- (I)), 1.34 (br s, 2H; -CH_2- (J)). ^{13}C NMR (105 MHz, $CDCl_3$): δ = 146.7, 141.3, 140.8, 137.1, 133.1, 128.94, 128.91, 127.6, 127.5, 127.3, 127.1, 64.4, 48.9, 45.1, 40.6. ^{11}B NMR (128 MHz, $CDCl_3$): δ = 28.9. Elemental Analyses calcd. for $C_{24}H_{24}BO_2$: C 81.14, H 6.81; found C 81.11, H 6.80.

Coordination studies

Compound **C1** was obtained according to the preparation procedure described in the main article, by using 30 mg (0.185 mmol) of boronic ester **0b** and 22.6 mg (0.185 mmol) of DMAP in 1 mL of THF. The mixture was stirred for 3 h at *rt*. The resulting product formed white crystals with 99% yield (0.052 g; 0.183 mmol).

Analytic data: ^1H NMR (600 MHz, $CDCl_3$): δ = 8.21 (dd, $J_{H,H}$ = 1.8, 5.4 Hz, 2H; (A)), 7.75 (dd, $J_{H,H}$ = 1.2, 7.8 Hz, 2H; (B)), 7.39 (tt, $J_{H,H}$ = 7.2 Hz, 1H; (C)), 7.32 (tt, 2H; (D)), 6.48 (dd, $J_{H,H}$ = 1.8, 4.8 Hz, 2H; (E)), 4.13 (t, $J_{H,H}$ = 5.4 Hz, 4H; -CH_2- (F)), 2.99 (s, 6H; -CH_3 (G)), 2.03 (p, $J_{H,H}$ = 5.4 Hz, 2H; -CH_2- (H)). ^{13}C NMR (105 MHz, $CDCl_3$): δ = 154.5, 149.0, 133.6, 130.5, 127.6, 127.4, 106.7, 62.0, 39.2, 27.6. ^{11}B NMR (128 MHz, $CDCl_3$): δ 26.3.

Compound **C2** was obtained according to the preparation procedure described in the main article, by using 30 mg (0.0125 mmol) of monomer **M1** and 15.3 mg (0.0125 mmol) of DMAP in 1 mL of THF. The mixture was stirred for 3 h at *rt*. The resulting product formed white crystals with 99% yield (0.045 g; 0.124 mmol).

Analytic data: ^1H NMR (600 MHz, $CDCl_3$): δ = 8.23 (dd, $J_{H,H}$ = 1.2, 4.8 Hz, 2H; (A)), 7.73 (dd, $J_{H,H}$ = 1.2, 7.8 Hz, 2H; (B)), 7.37-7.33 (m, 1H; (C)), 7.30-7.28 (m, 2H; (D)), 6.49 (dd, $J_{H,H}$ = 1.8, 5.4 Hz, 2H; (E)), 6.20 (t, $J_{H,H}$ = 1.8 Hz, 2H; -C*H*=C*H*- (F)), 4.24 (t, $J_{H,H}$ = 12.0 Hz, 2H; -CH_2- (G)), 4.08 (dd, $J_{H,H}$ = 4.2, 12.0 Hz, 2H; -CH_2- (H)), 3.74 (m, THF), 3.00 (s, 6H; -CH_3 (I)), 2.54 (p, $J_{H,H}$ = 1.8 Hz, 2H; -C*H*- (J)), 2.11-2.06 (m, 2H; -C*H*- (K)), 1.84 (m, THF), 1.50-1.49 (d, $J_{H,H}$ = 9.0 Hz, 1H; -CH_2- (L)), 1.30 (dt, $J_{H,H}$ = 0.6, 9.0 Hz, 1H; -CH_2- (L’)). ^{13}C NMR (105 MHz, $CDCl_3$): δ = 154.7, 148.3, 137.3, 133.9, 133.7, 129.9, 127.5, 127.4, 106.6, 68.1 (THF), 67.0, 45.0, 43.3, 43.0, 39.2, 25.7 (THF). ^{11}B NMR (128 MHz, $CDCl_3$): δ = 24.7

Compound **C3** was obtained according the preparation procedure described in the main article, by using 20 mg of polymer **P1** and 10.2 mg (0.083 mmol) of DMAP in 0.6 mL of THF. The mixture was stirred for 24 h at *rt*. The resulting product formed membrane upon evaporation with 99% yield (0.0198 g).

Analytic data: ^1H NMR (600 MHz, $CDCl_3$): δ = 8.24 (br d, 2H; (A)), 7.71 (br s, 2H; (B)), 7.36-7.35 (m, 1H; (C)), 7.30 (br s, 2H; (D)), 6.51-6.49 (d, $J_{H,H}$ = 7.2Hz, 2H; (E)), 5.39 (br d, 2H; -C*H*=C*H*- (F)), 4.05 (br s, 4H; -CH_2- (G)), 3.74 (m, THF), 3.01 (s, 6H; -CH_3 (H)), 2.22 (br s, 2H; -C*H*- (I)), 1.89 (br s, 2H; -C*H*- (J)), 1.84 (m, THF), 1.31 (br s, 2H; -CH_2- (K)). ^{13}C NMR (105 MHz, $CDCl_3$): δ = 155.2, 146.4, 133.8, 133.7, 129.2, 127.50, 127.47, 106.6, 68.1 (THF), 63.9, 49.1, 44.8, 42.2, 39.4, 25.7 (THF). ^{11}B NMR (128 MHz, $CDCl_3$): δ = 20.3

NMR Spectra

Preliminary studies

Compound 0a - 2-phenyl-1,3,2-dioxaborolane

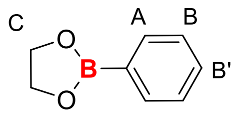

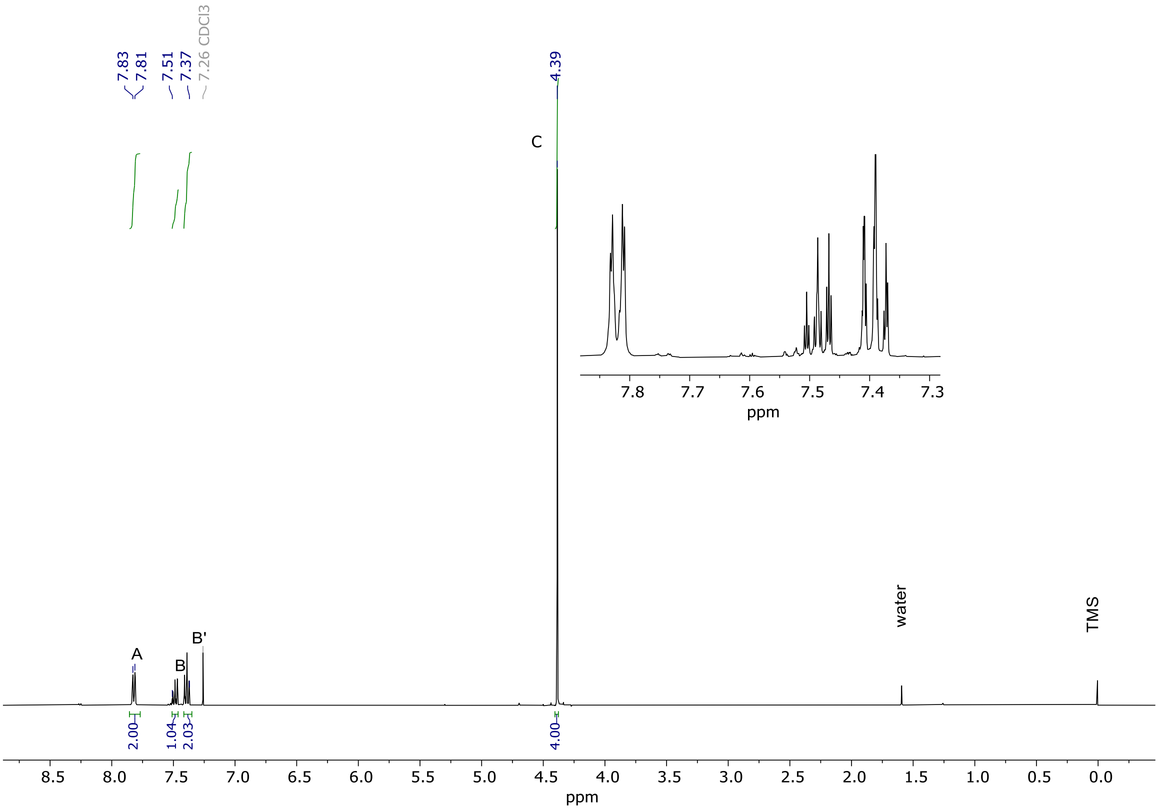

Figure S8. Compound 0a – ^{1}H NMR in $CDCl_3$

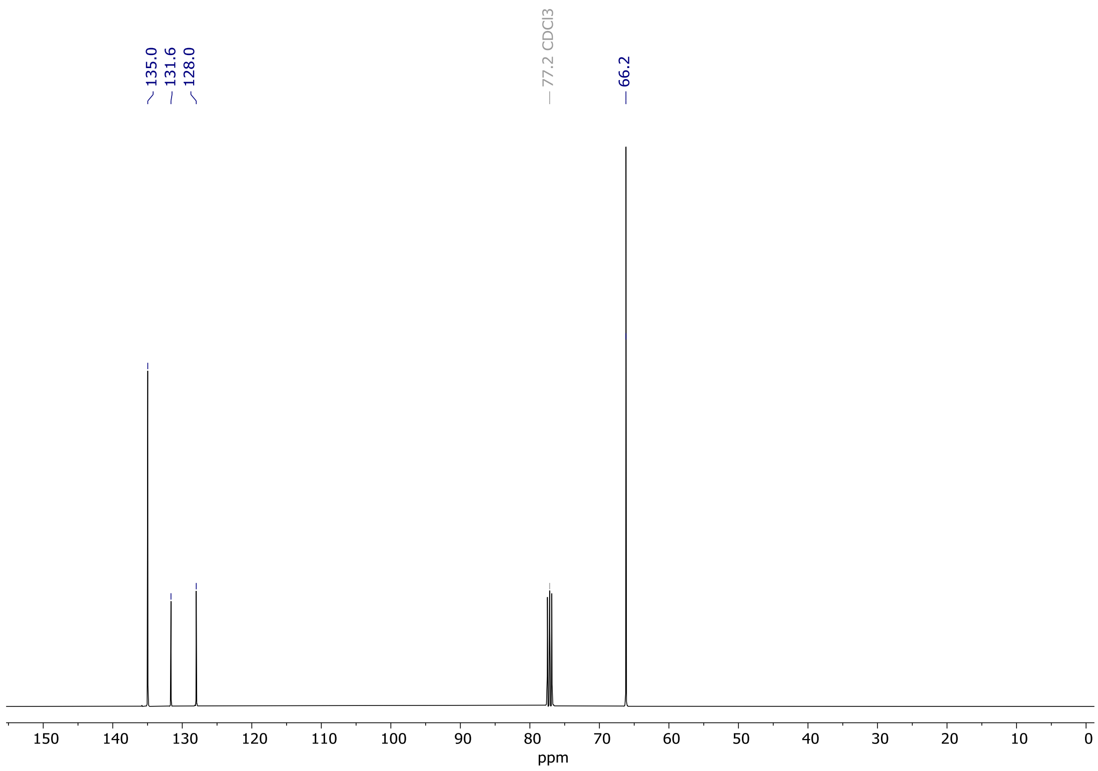

Figure S9. Compound 0a – ^{13}C NMR in $CDCl_3$

Compound 0b - 2-phenyl-1,3,2-dioxaborinane

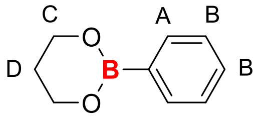

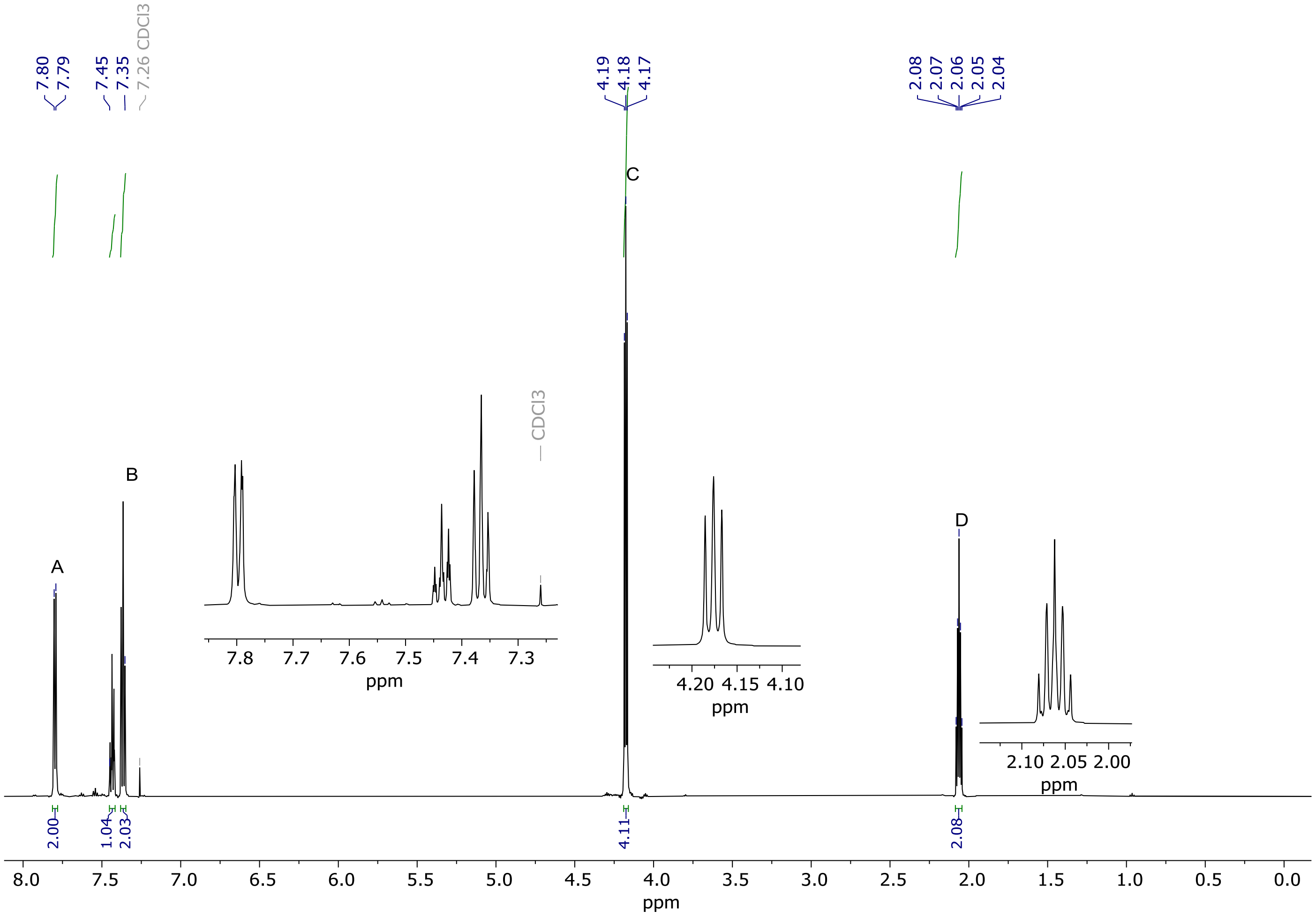

Figure S10. Compound 0b – ^{1}H NMR in $CDCl_3$

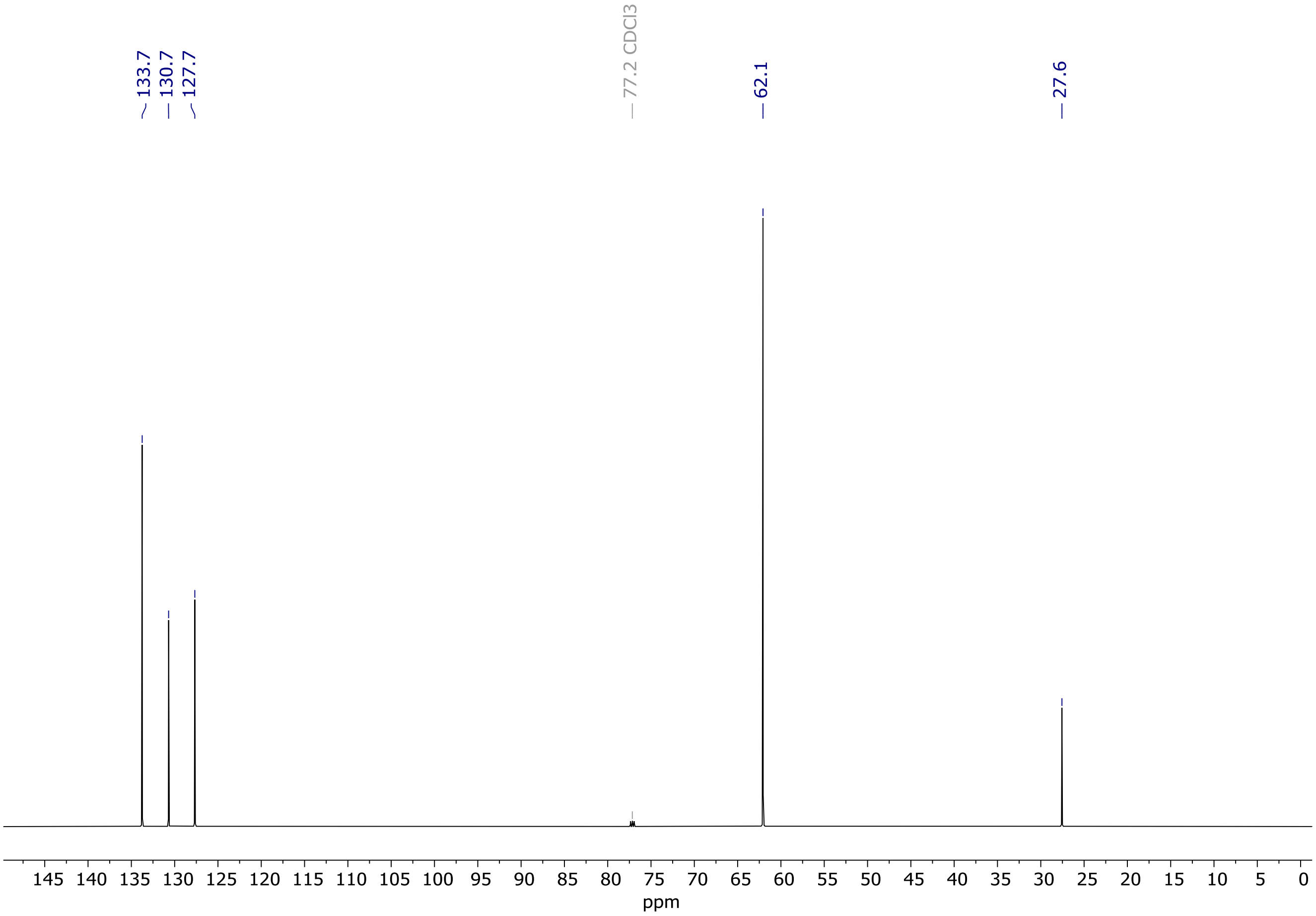

Figure S11. Compound 0b – ^{13}C NMR in $CDCl_3$

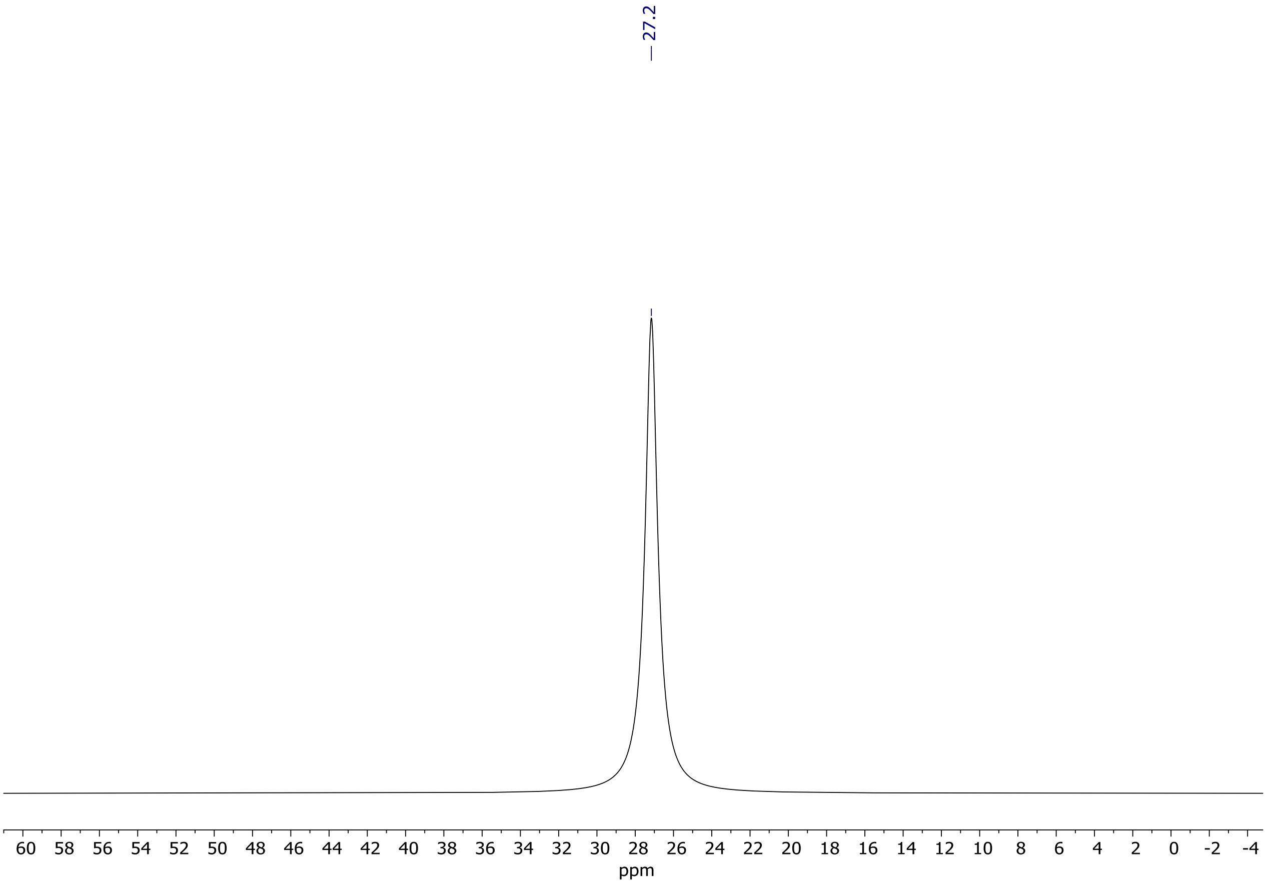

Figure S12. Compound 0b – ^{11}B NMR in $CDCl_3$

Monomers

Compound M1

5-norbornene-2-*exo*,3-*exo*-dimethyl-[(phenyl)-dioxaborepine]

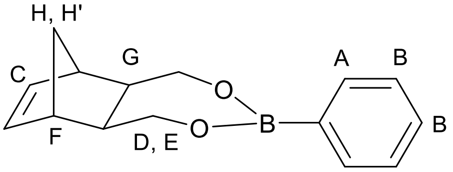

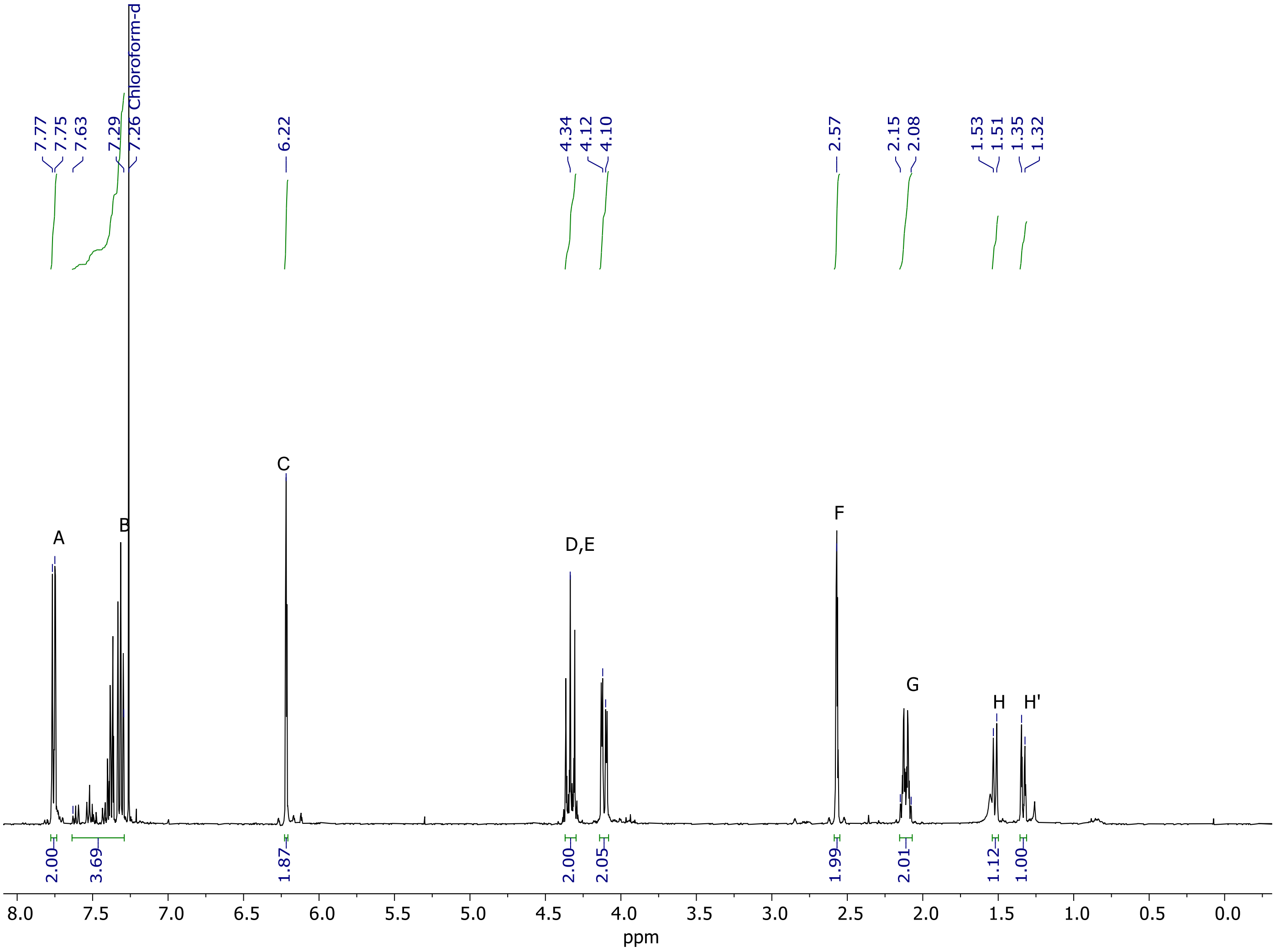

Figure S13. Compound M1 – ^{1}H NMR in $CDCl_3$

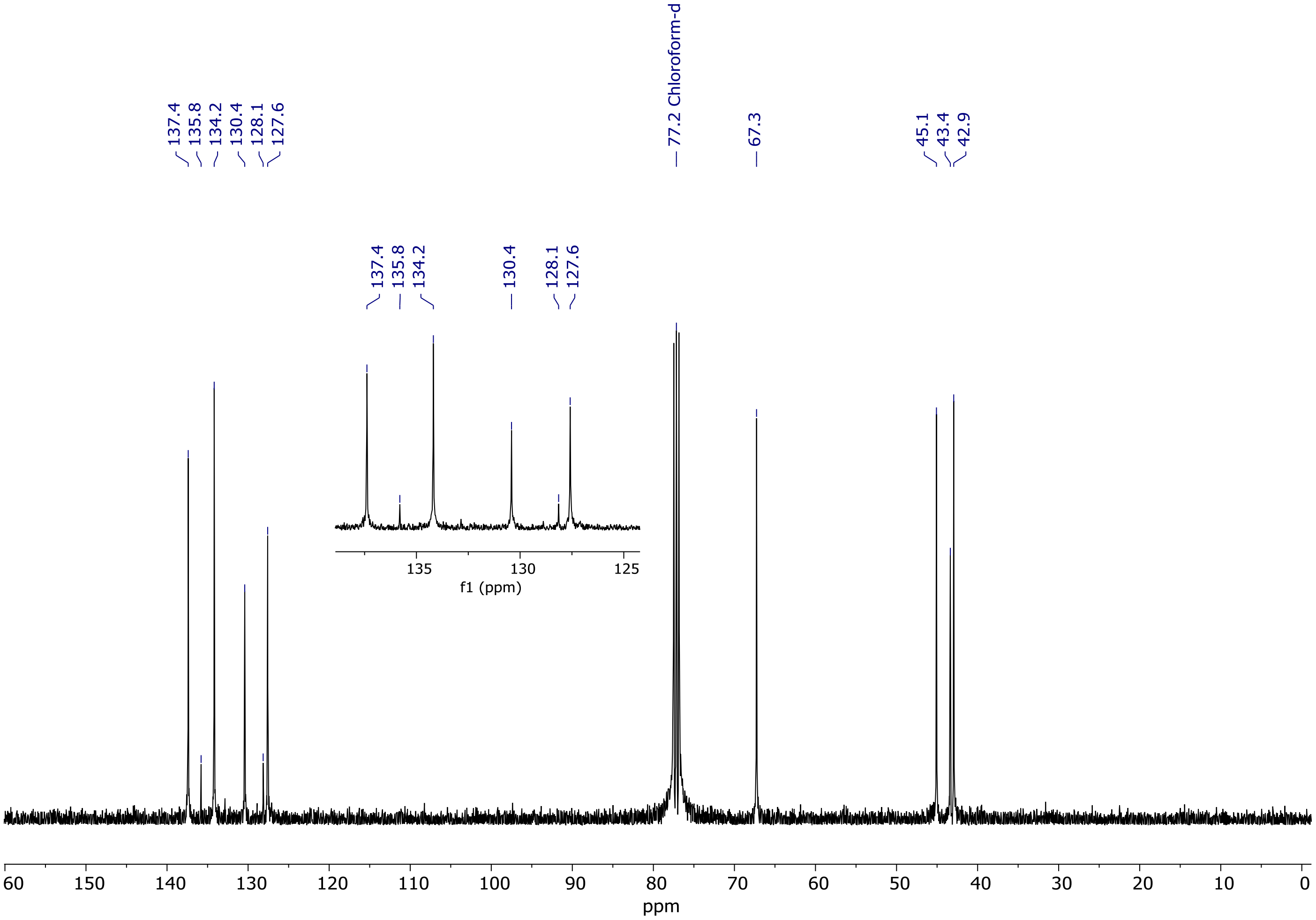

Figure S14. Compound M1 – ^{13}C NMR in $CDCl_3$

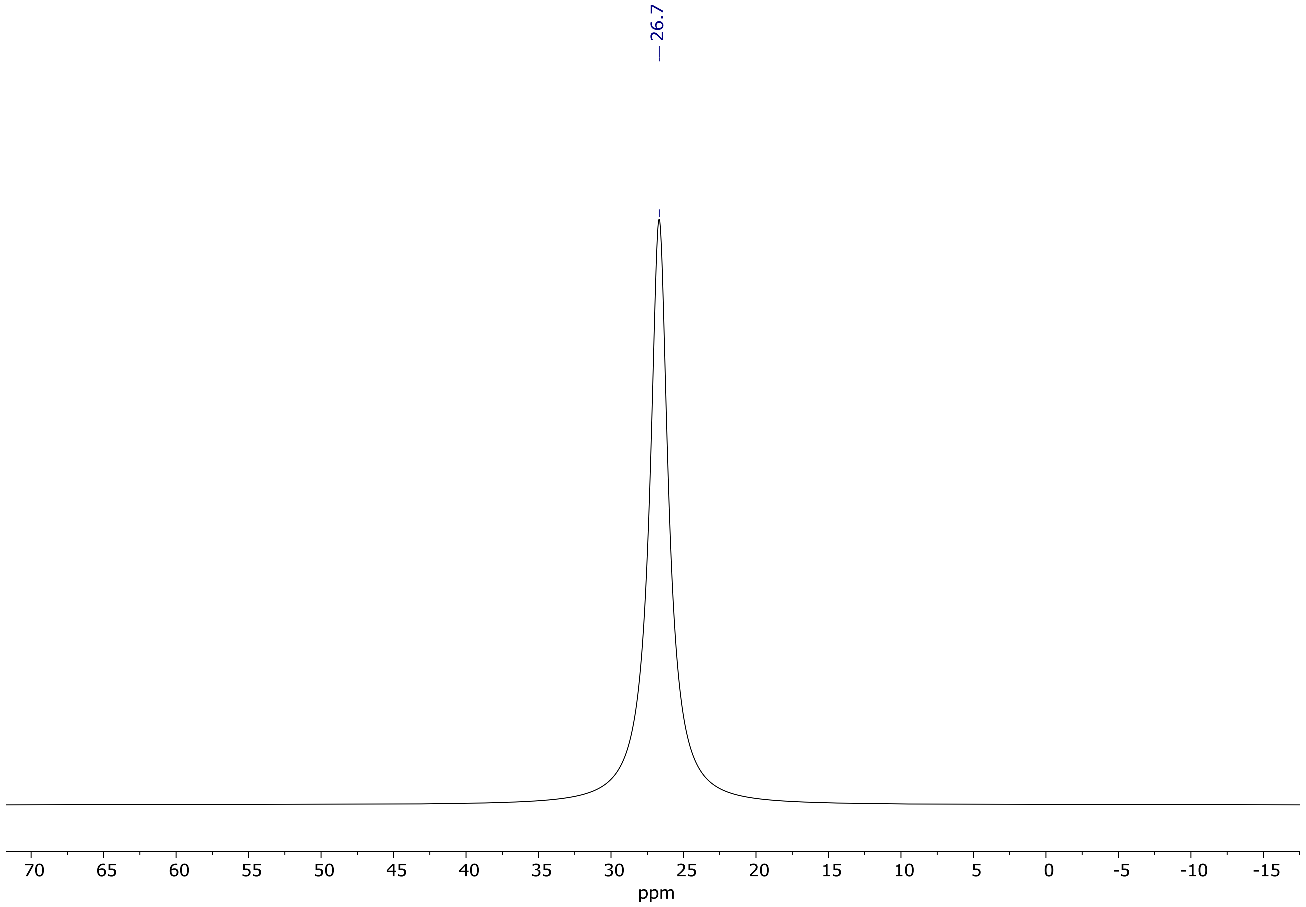

Figure S15. Compound M1 – ^{11}BNMR in CDCl3

Compound M2

5-norbornene-2-*exo*,3-*exo*-dimethyl-[(pyrene-1)-dioxaborepine]

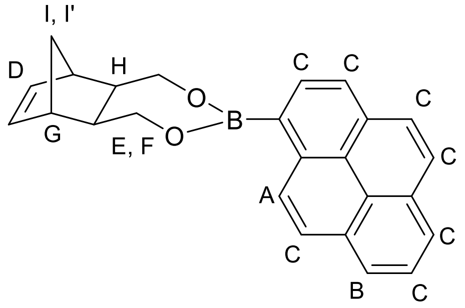

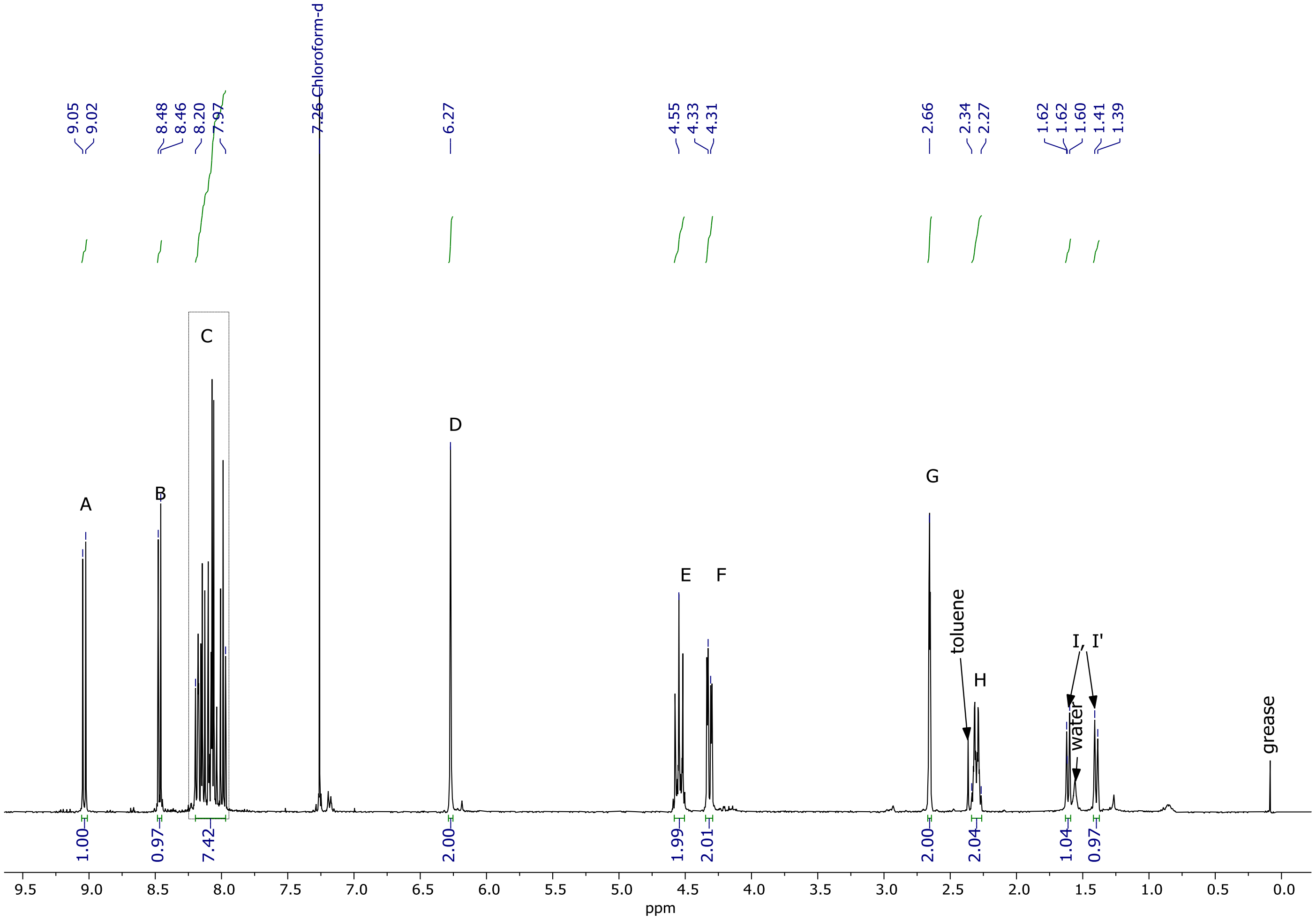

Figure S16. Compound M2 – ^{1}H NMR in $CDCl_3$

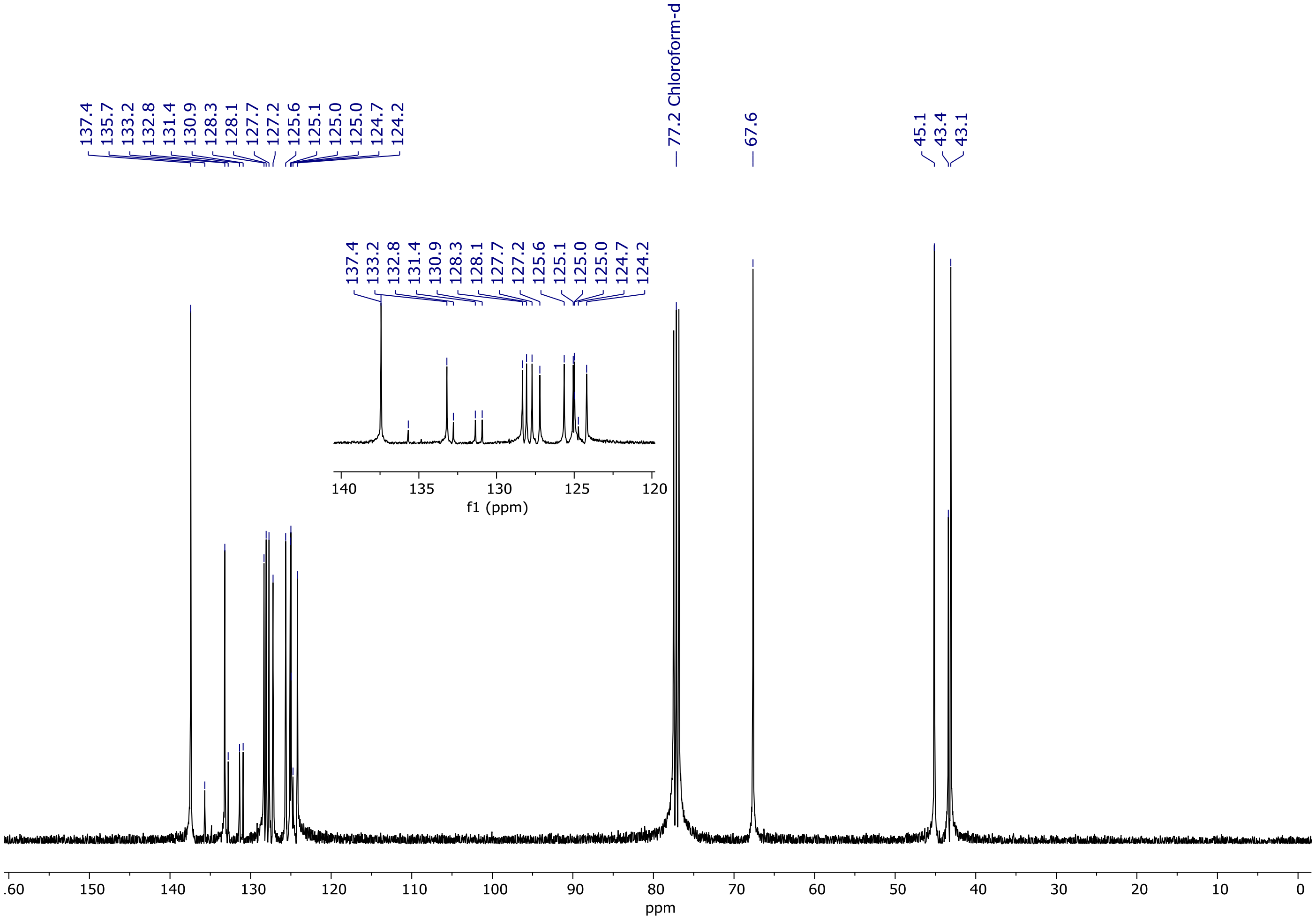

Figure S17. Compound M2 – ^{13}C NMR in $CDCl_3$

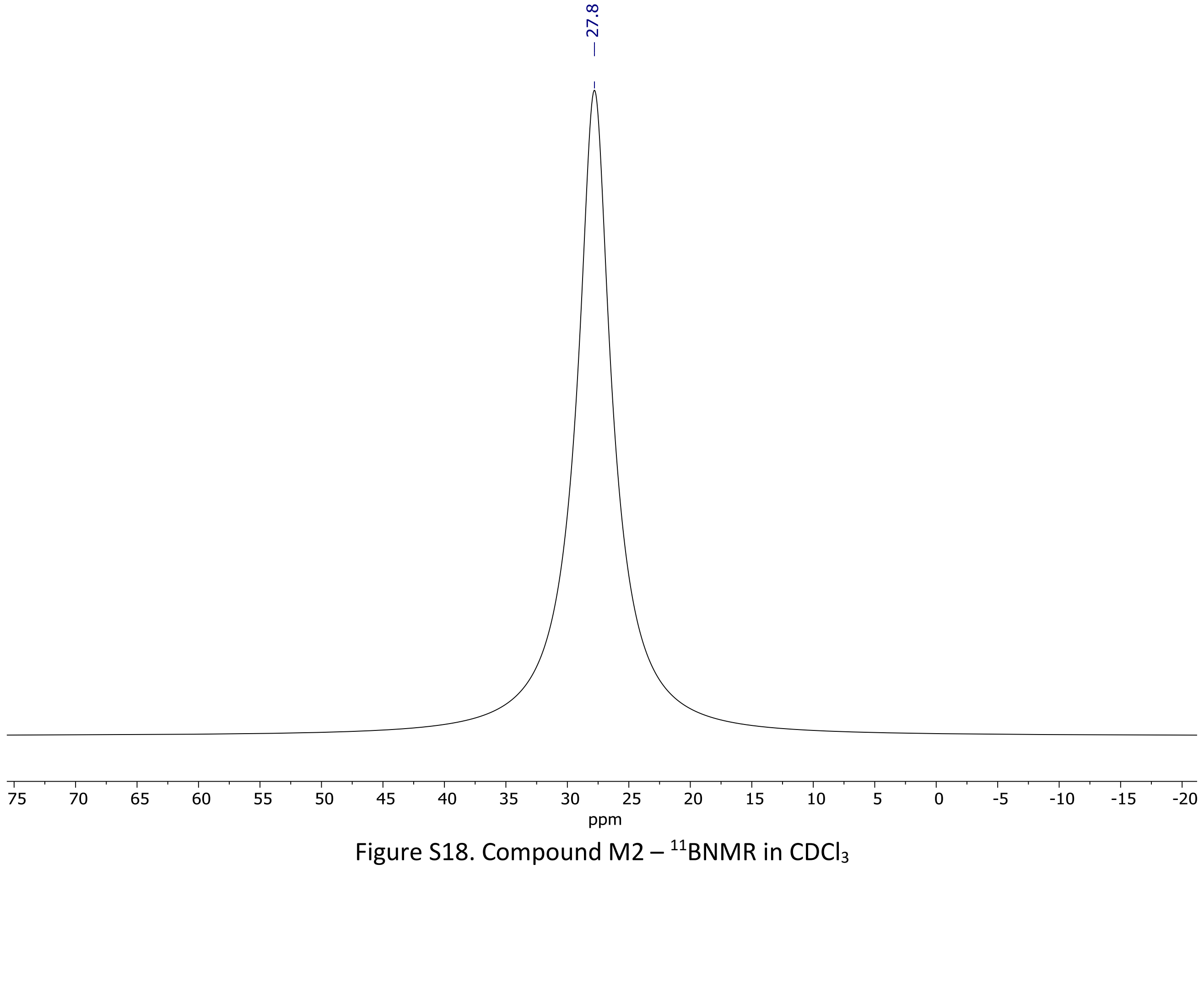

Figure S18. Compound M2 – ^{11}BNMR in $CDCl_3$

Compound M3

5-norbornene-2-*exo*,3-*exo*-dimethyl-[(9-phenantracenyl)-dioxaborepine]

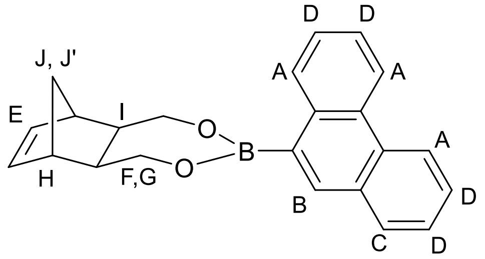

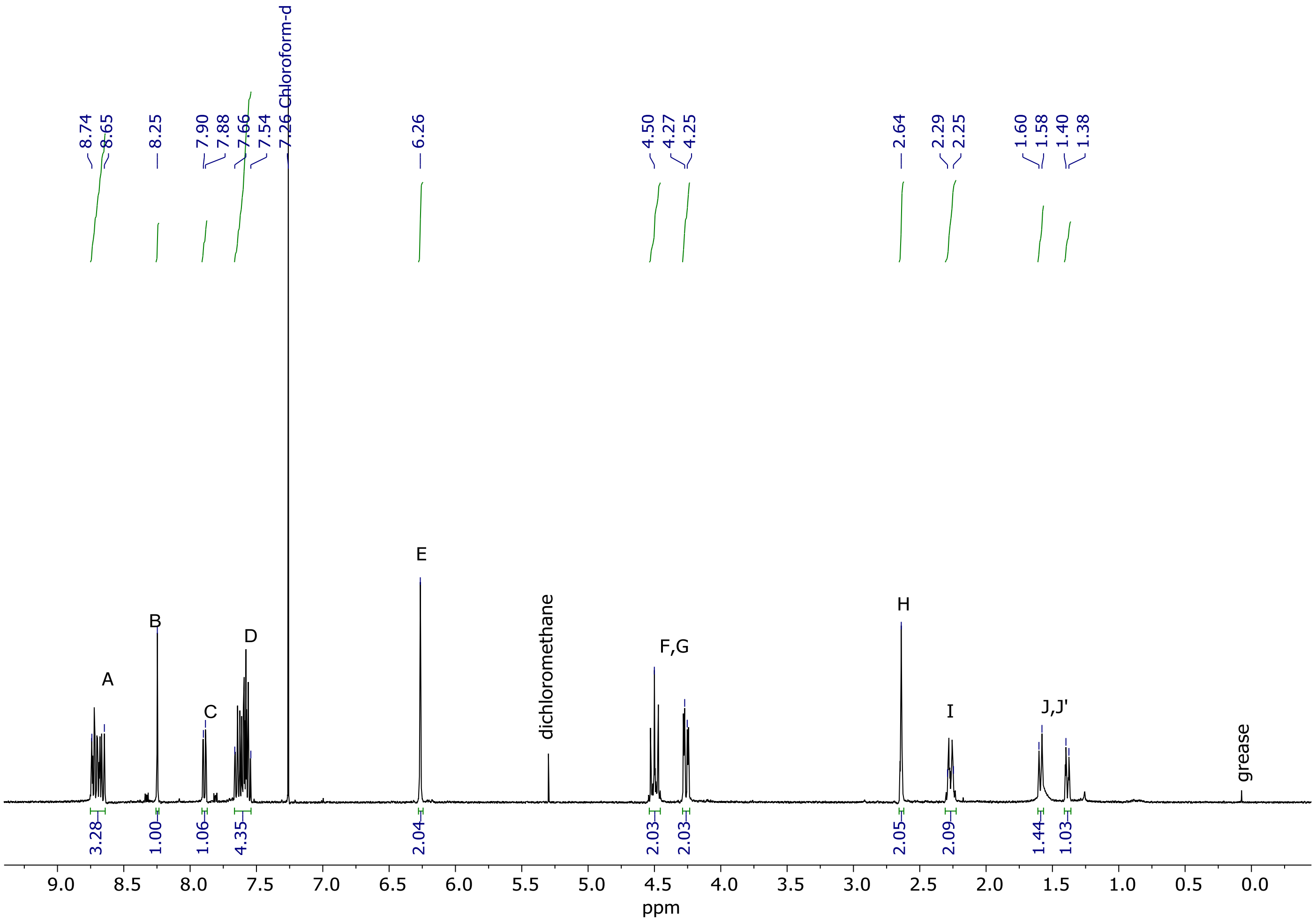

Figure S19. Compound M3 – ^{1}H NMR in $CDCl_3$

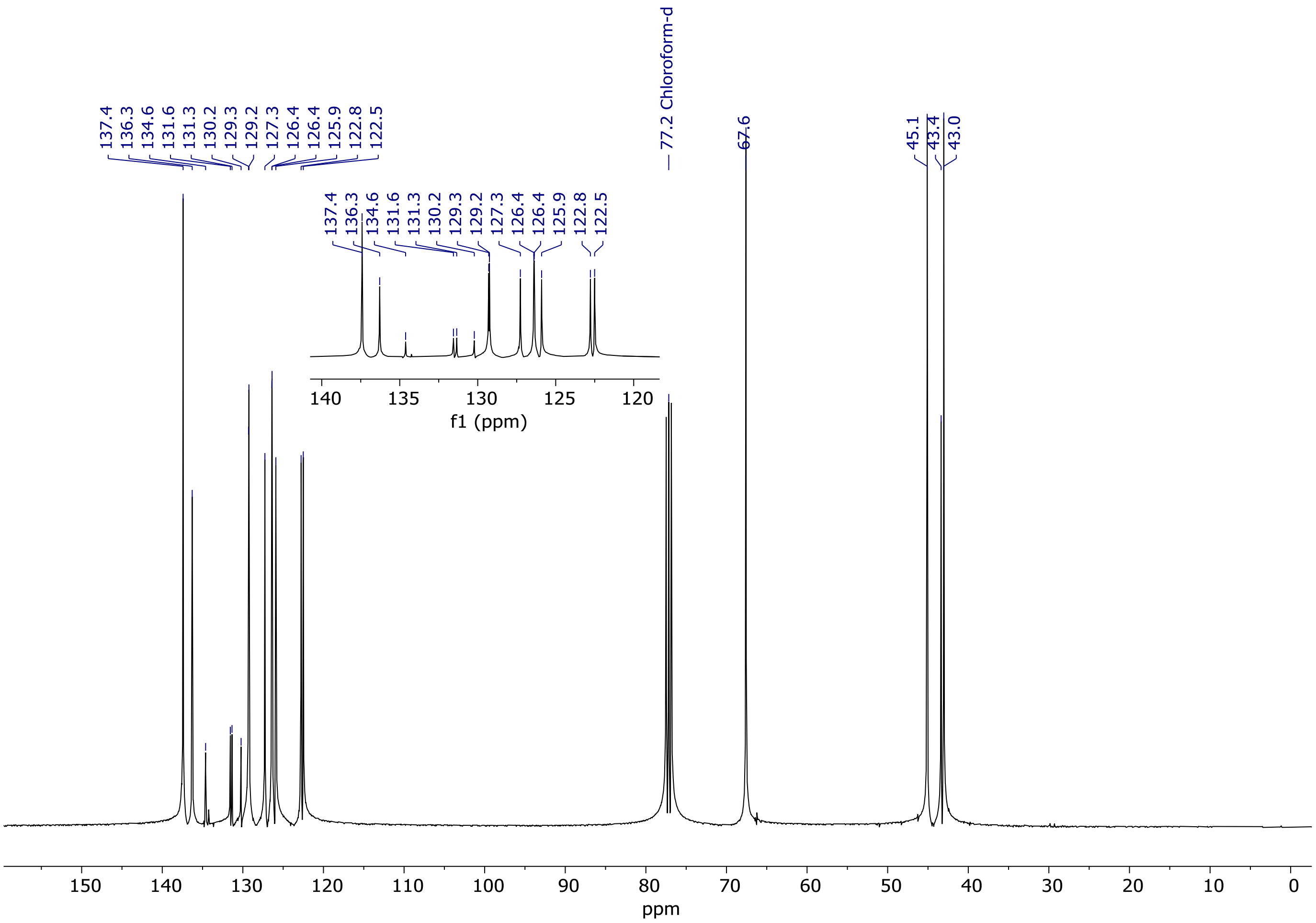

Figure S20. Compound M3 – ^{13}C NMR in $CDCl_3$

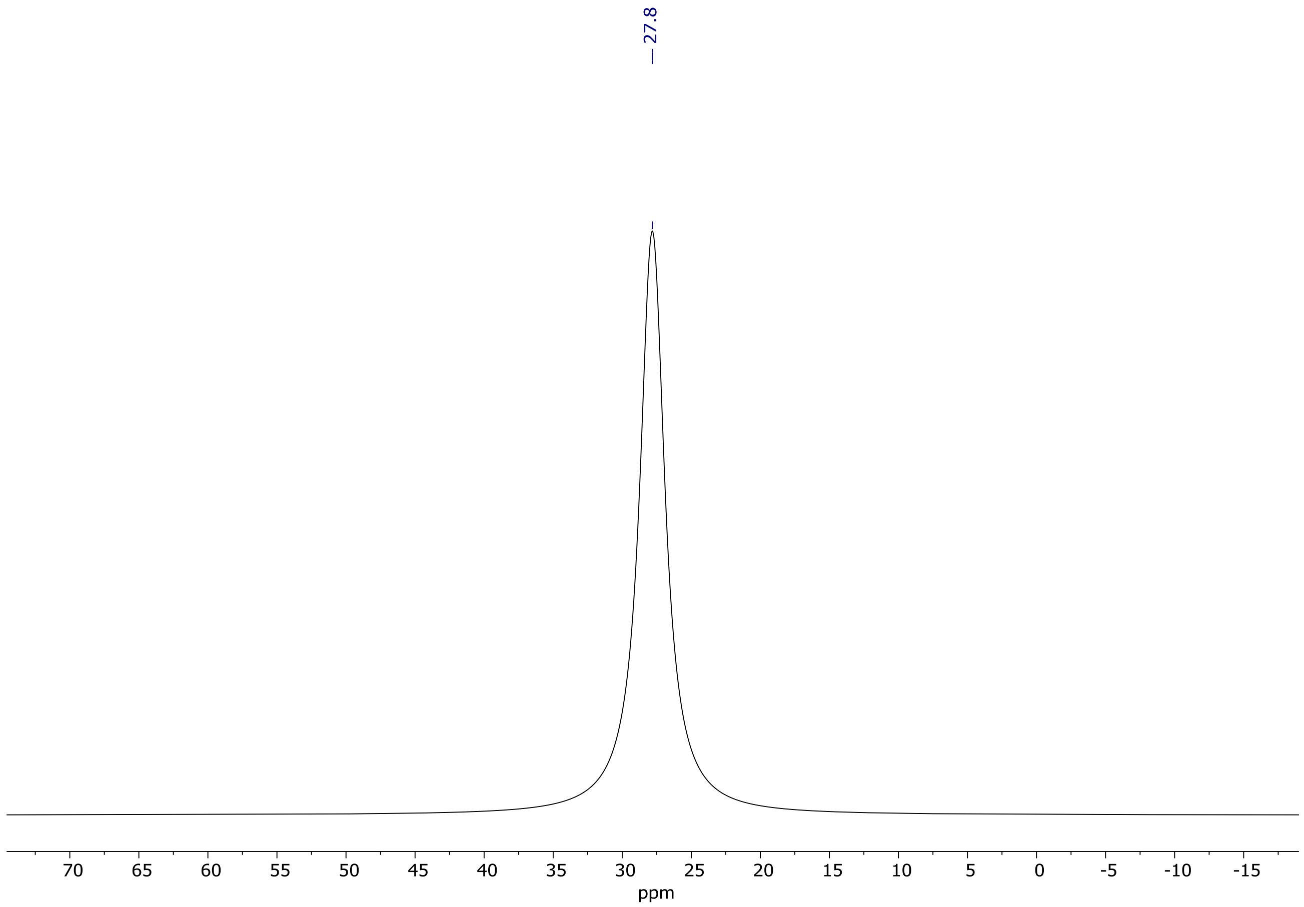

Figure S21. Compound M3 – ^{11}BNMR in $CDCl_3$

Compound M4

5-norbornene-2-*exo*,3-*exo*-dimethyl-[(10-bromoanthracene-9)-dioxaborepine]

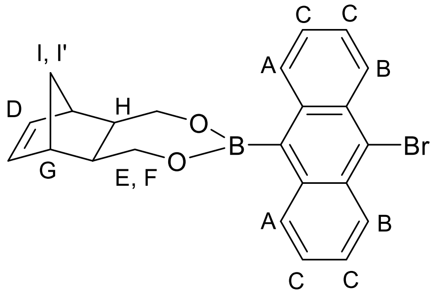

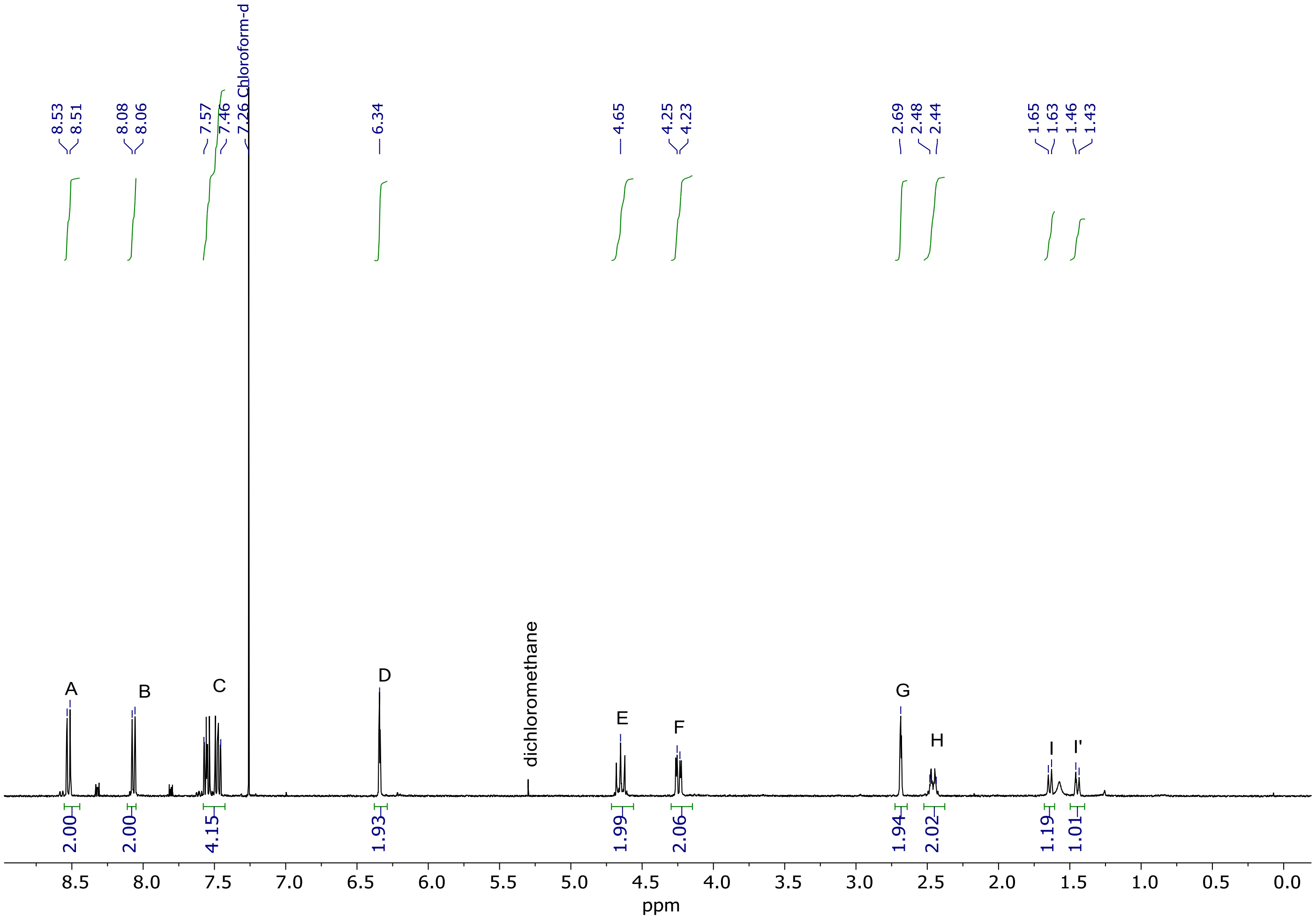

Figure S22. Compound M4 – ^{1}H NMR in $CDCl_3$

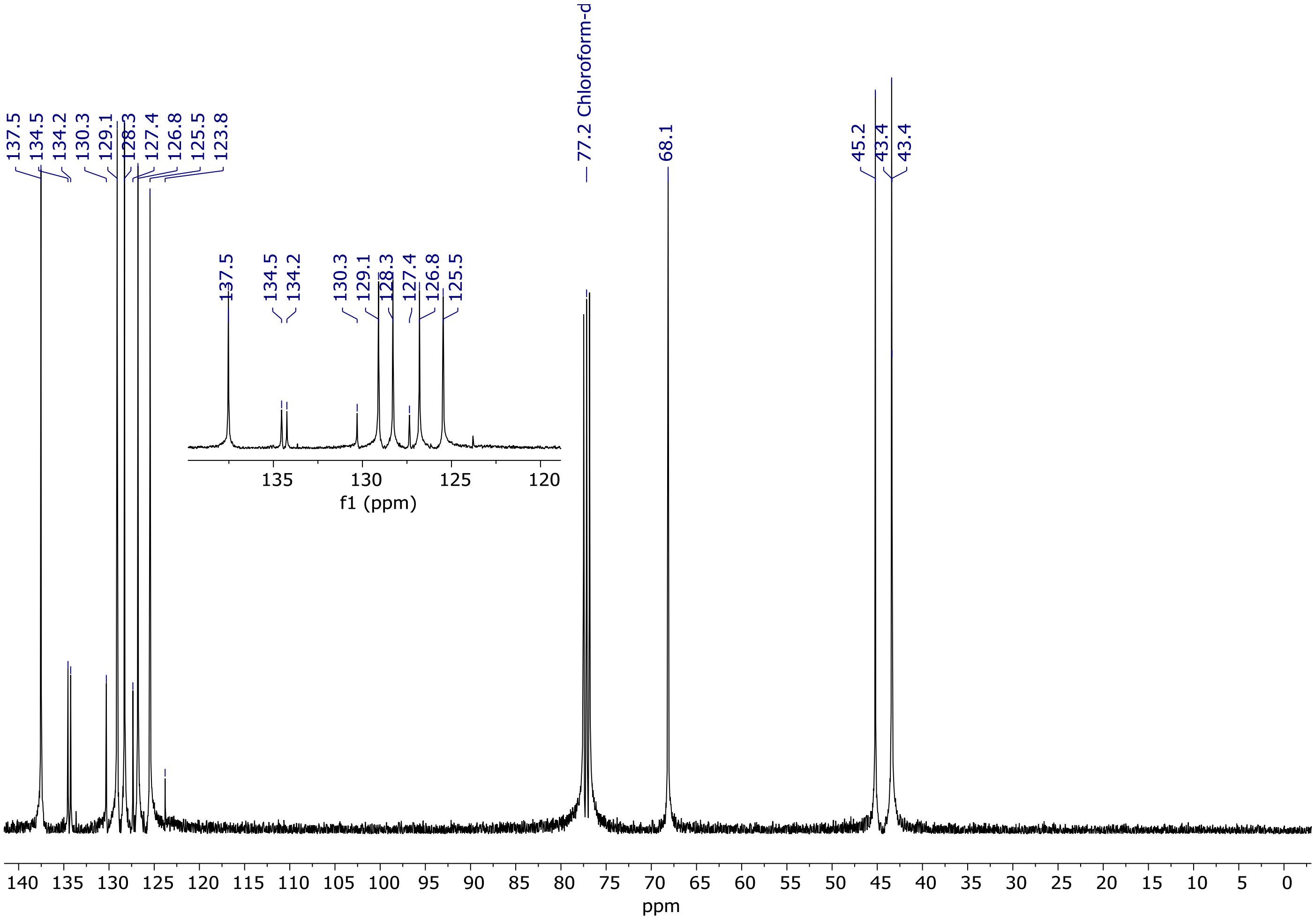

Figure S23. Compound M4 – ^{13}C NMR in $CDCl_3$

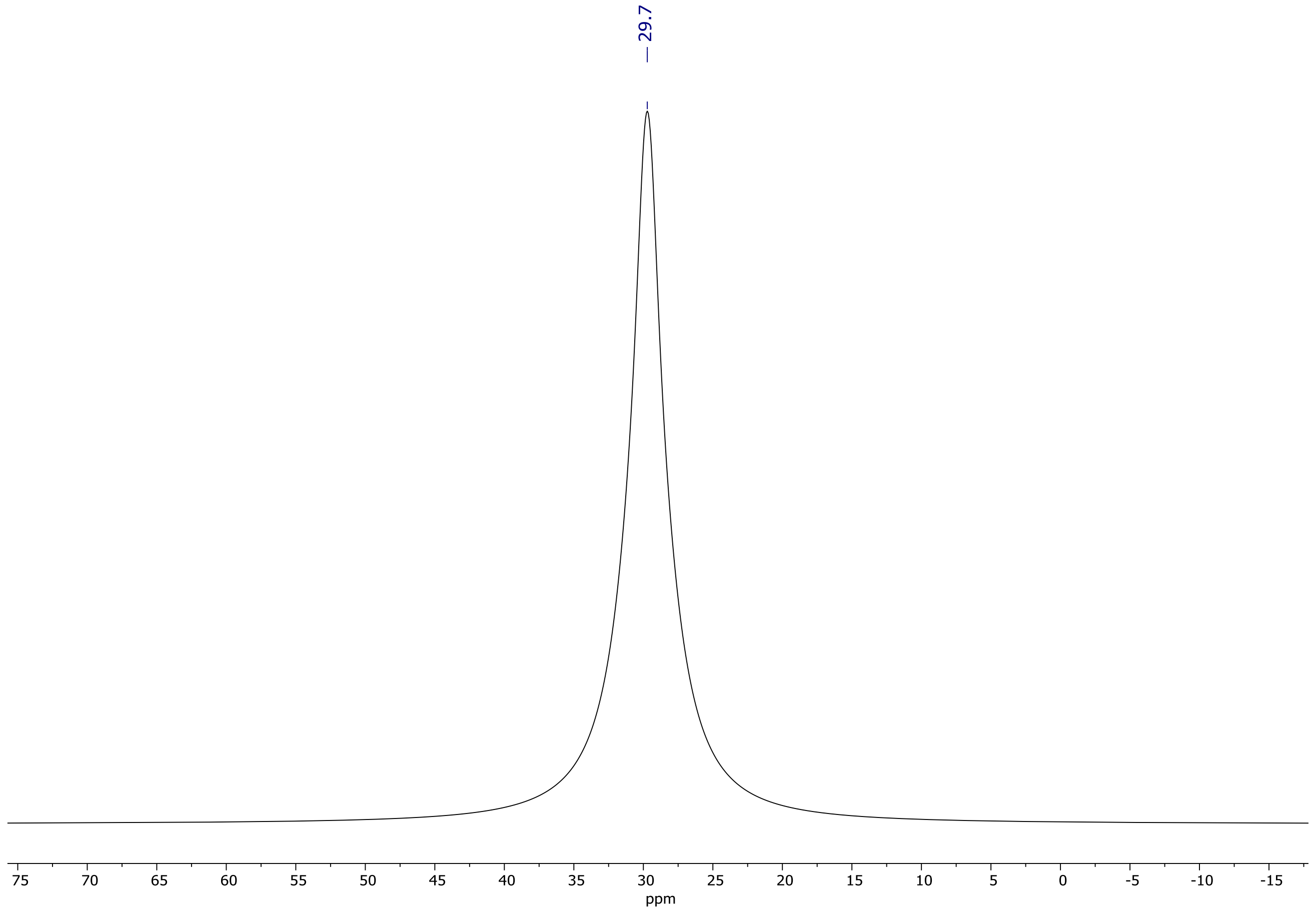

Figure S24. Compound M4 – ^{11}B NMR in $CDCl_3$

Compound M5

5-norbornene-2-*exo*,3-*exo*-dimethyl-[(3,5-dibromophenyl)-dioxaborepine]

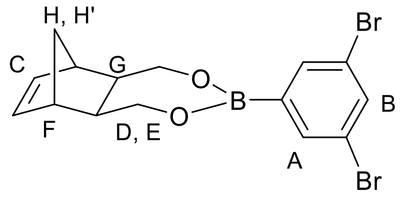

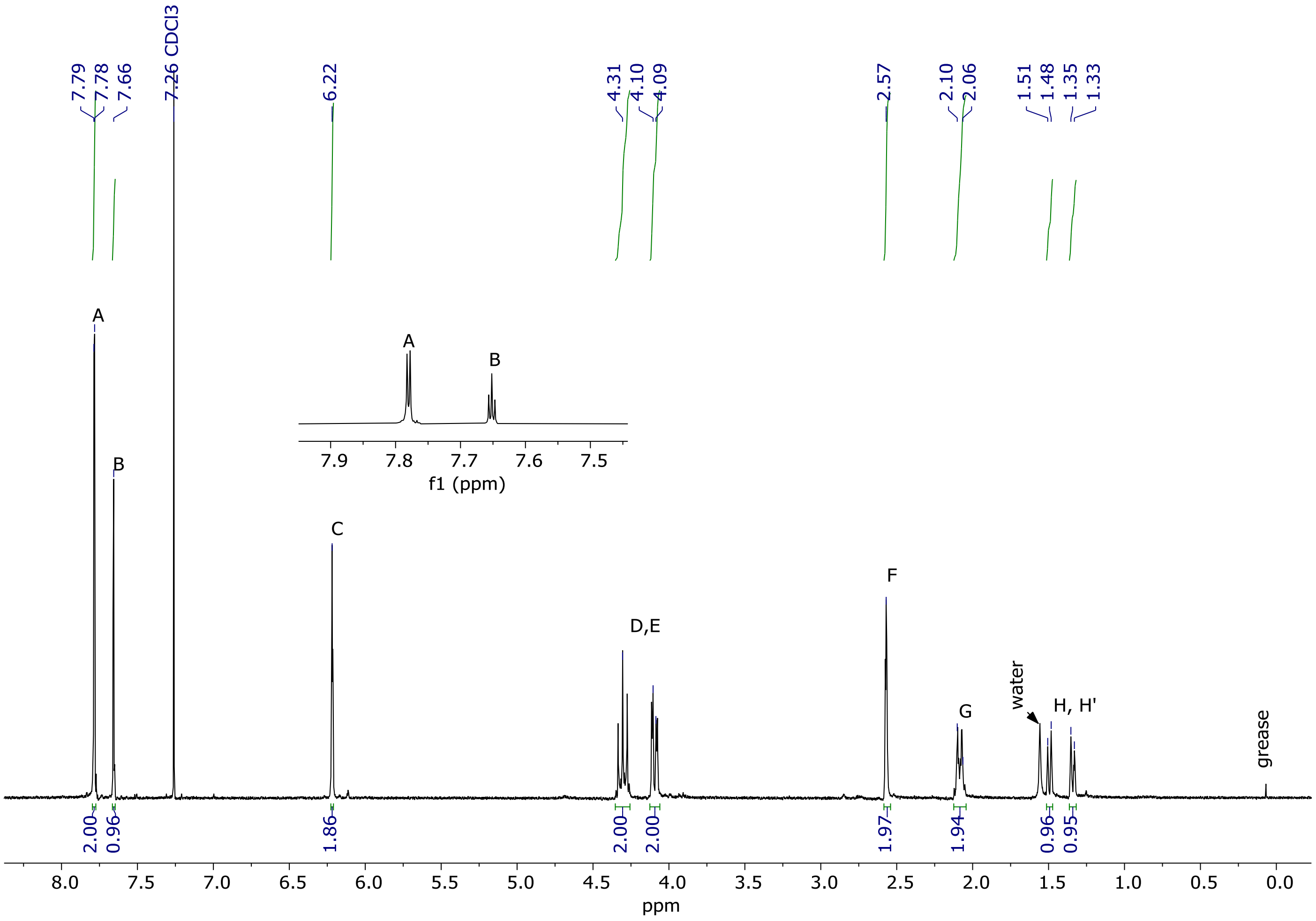

Figure S25. Compound M5 – ^{1}H NMR in $CDCl_3$

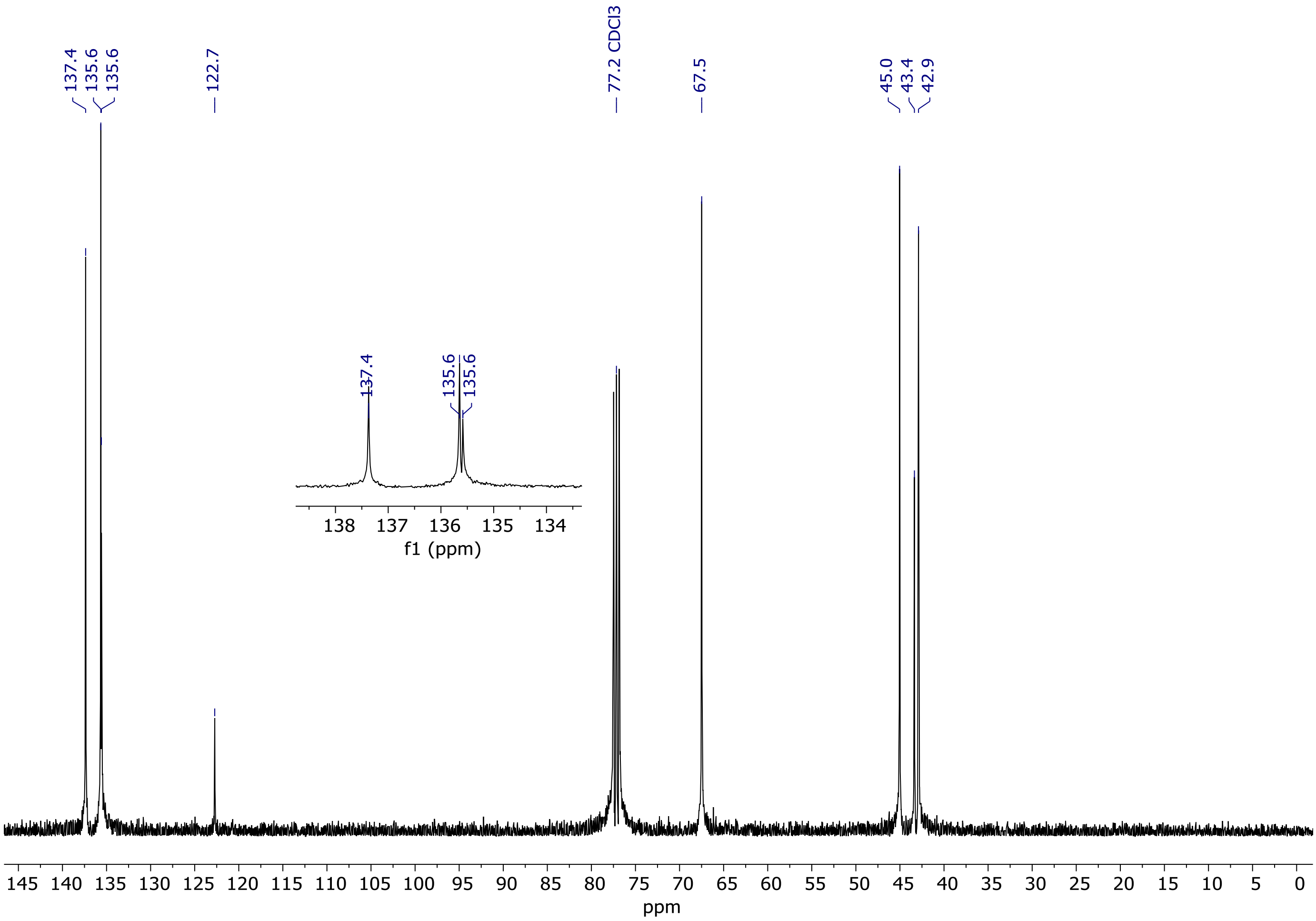

Figure S26. Compound M5 – ^{13}C NMR in $CDCl_3$

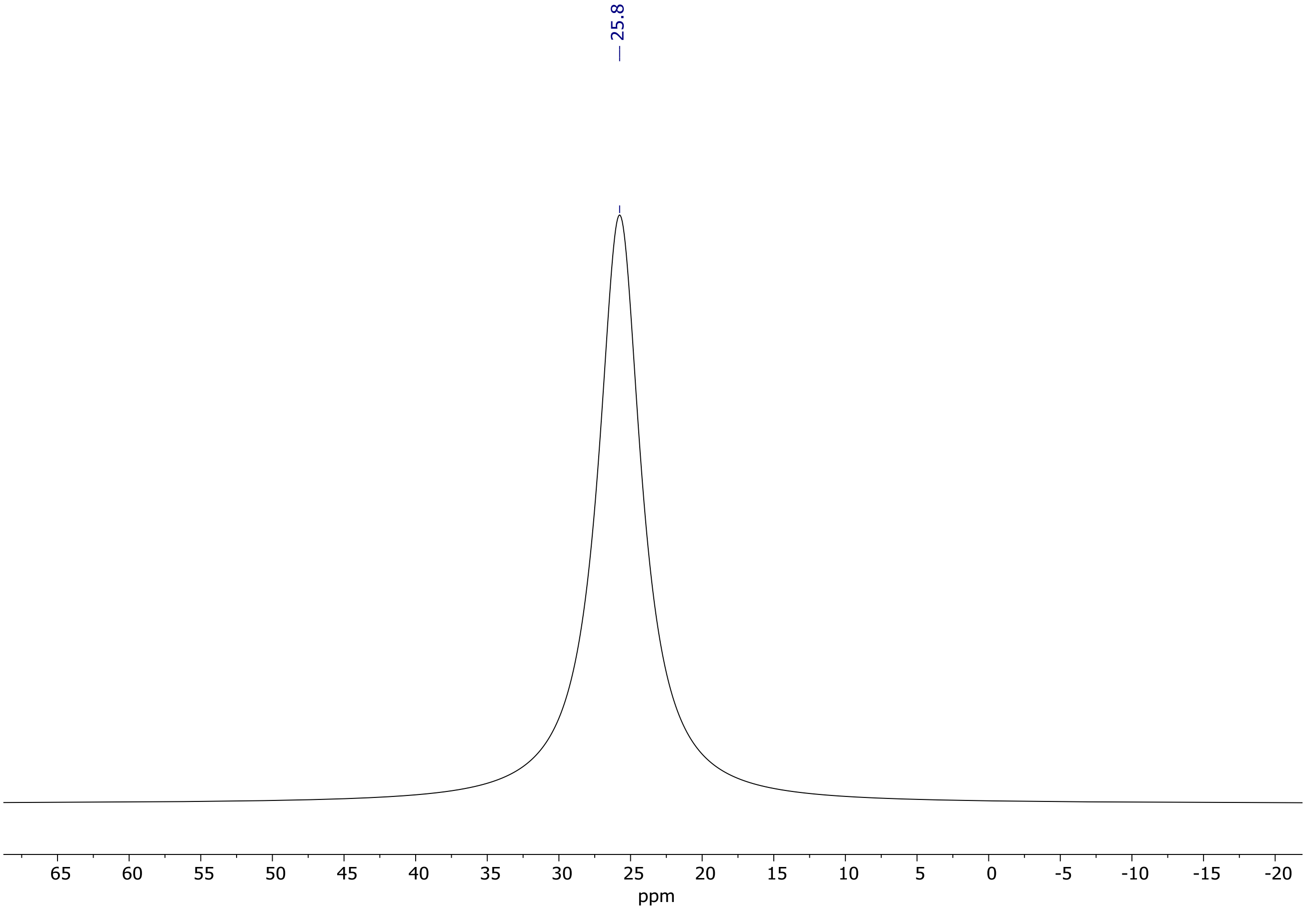

Figure S27. Compound M5 – ^{11}B NMR in $CDCl_3$

Compound M6

5-norbornene-2-*exo*,3-*exo*-dimethyl-[(trans-2-(4-methylphenyl)vinyl)-dioxaborepine]

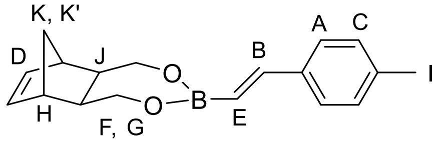

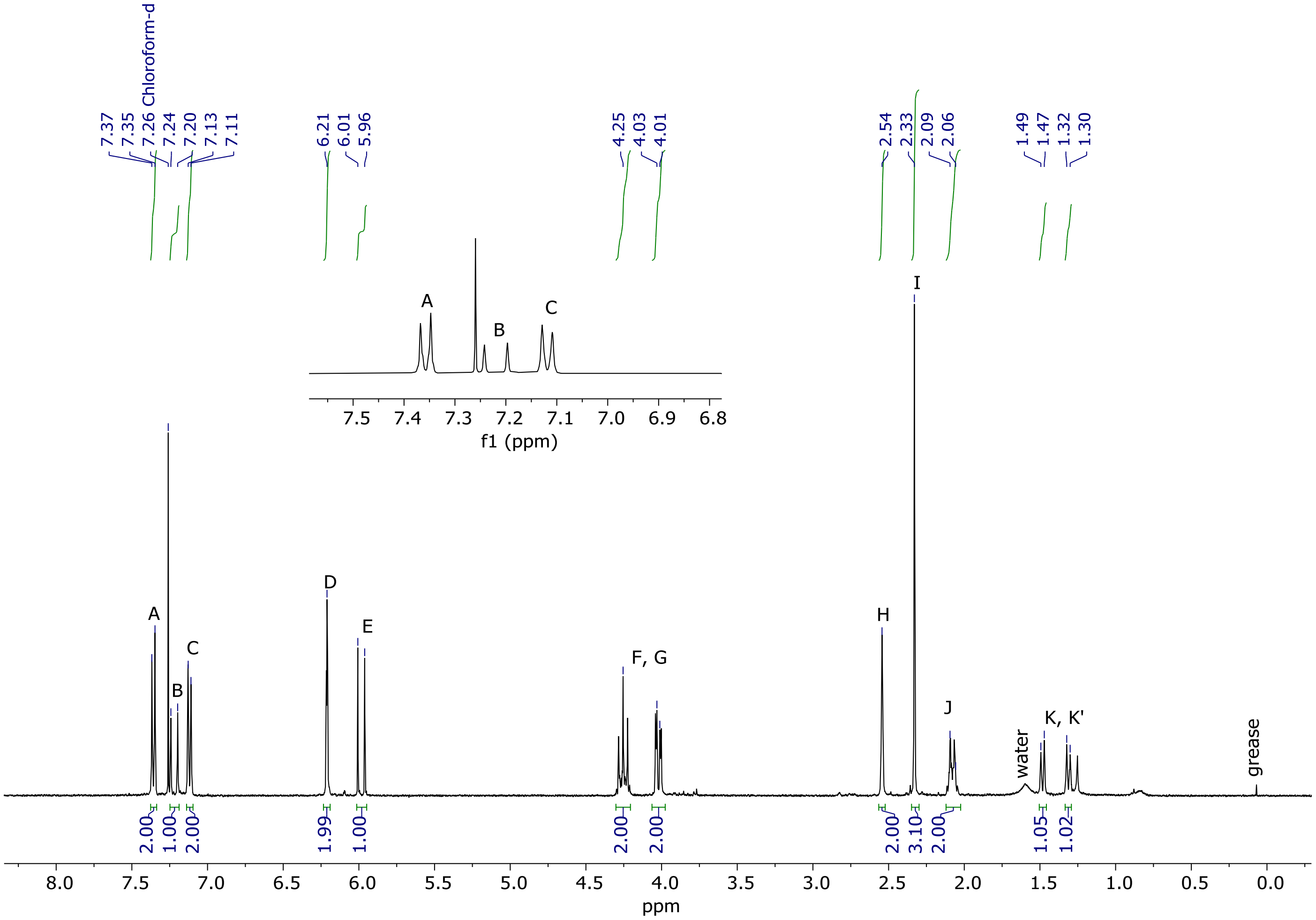

Figure S28. Compound M6 – ^{1}H NMR in $CDCl_3$

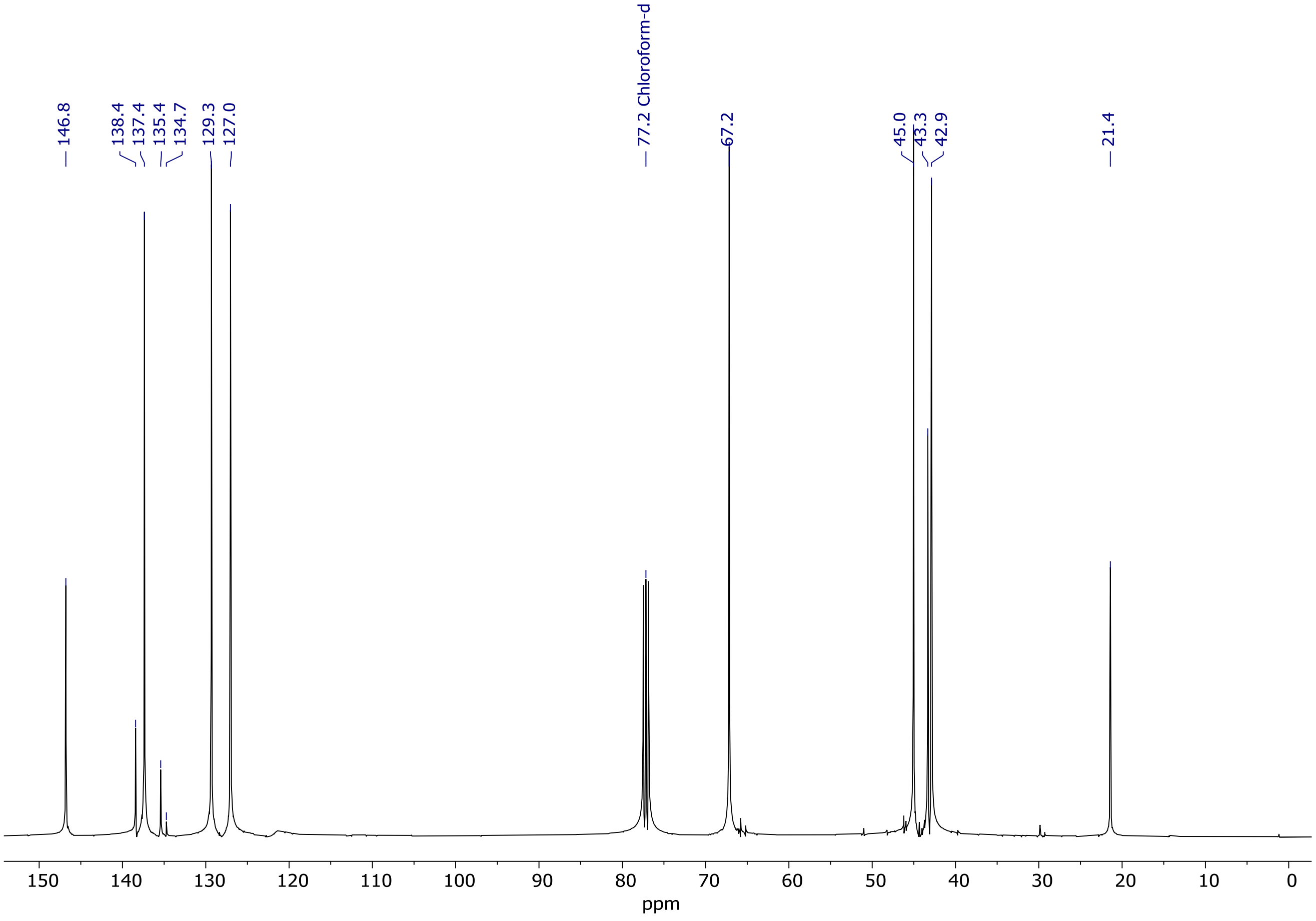

Figure S29. Compound M6 – ^{13}C NMR in $CDCl_3$

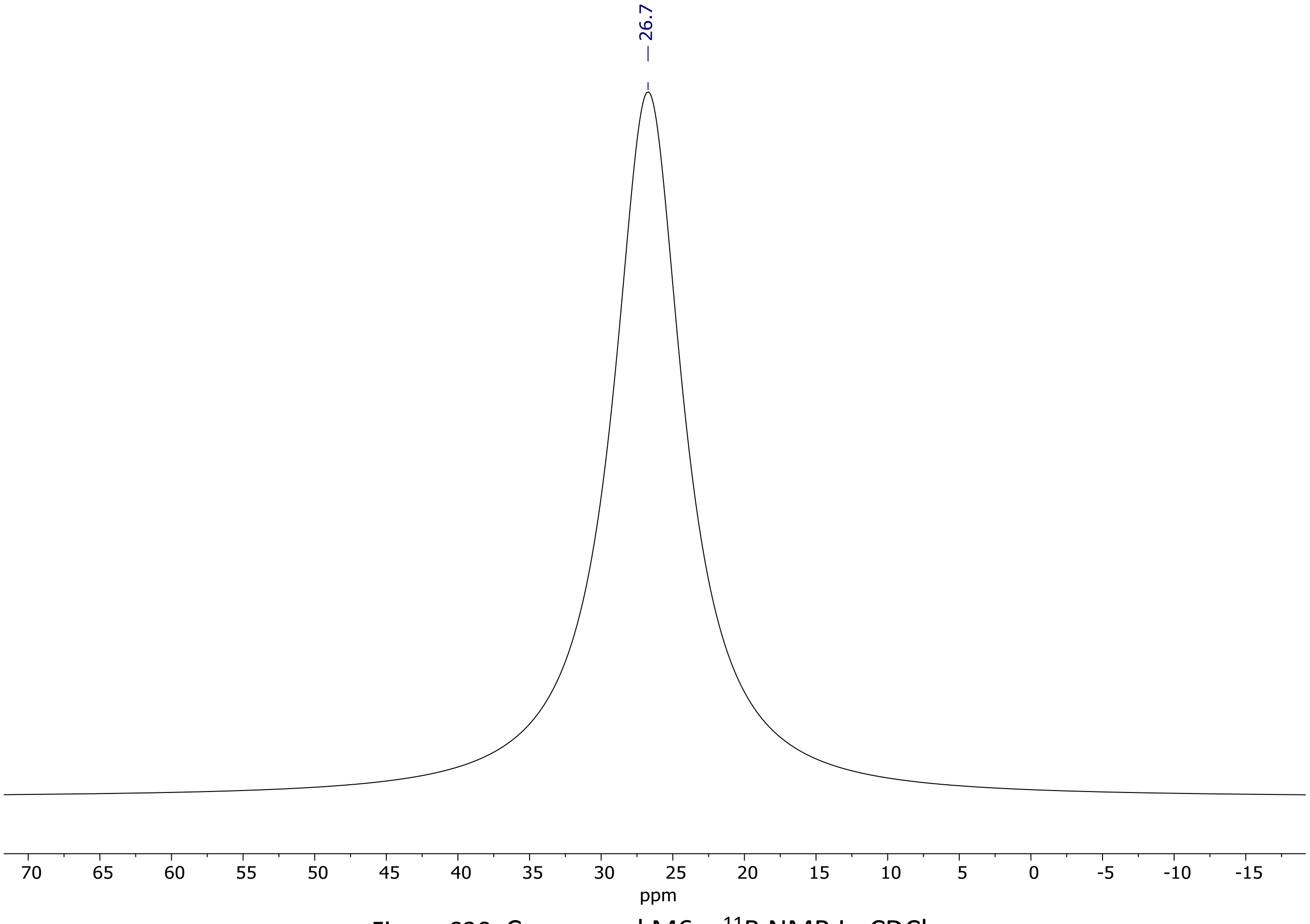

Figure S30. Compound M6 – ^{11}B NMR In $CDCl_3$

Compound M7

5-norbornene-2-*exo*,3-*exo*-dimethyl-[(trans-2-(4-biphenyl)vinyl)-dioxaborepine]

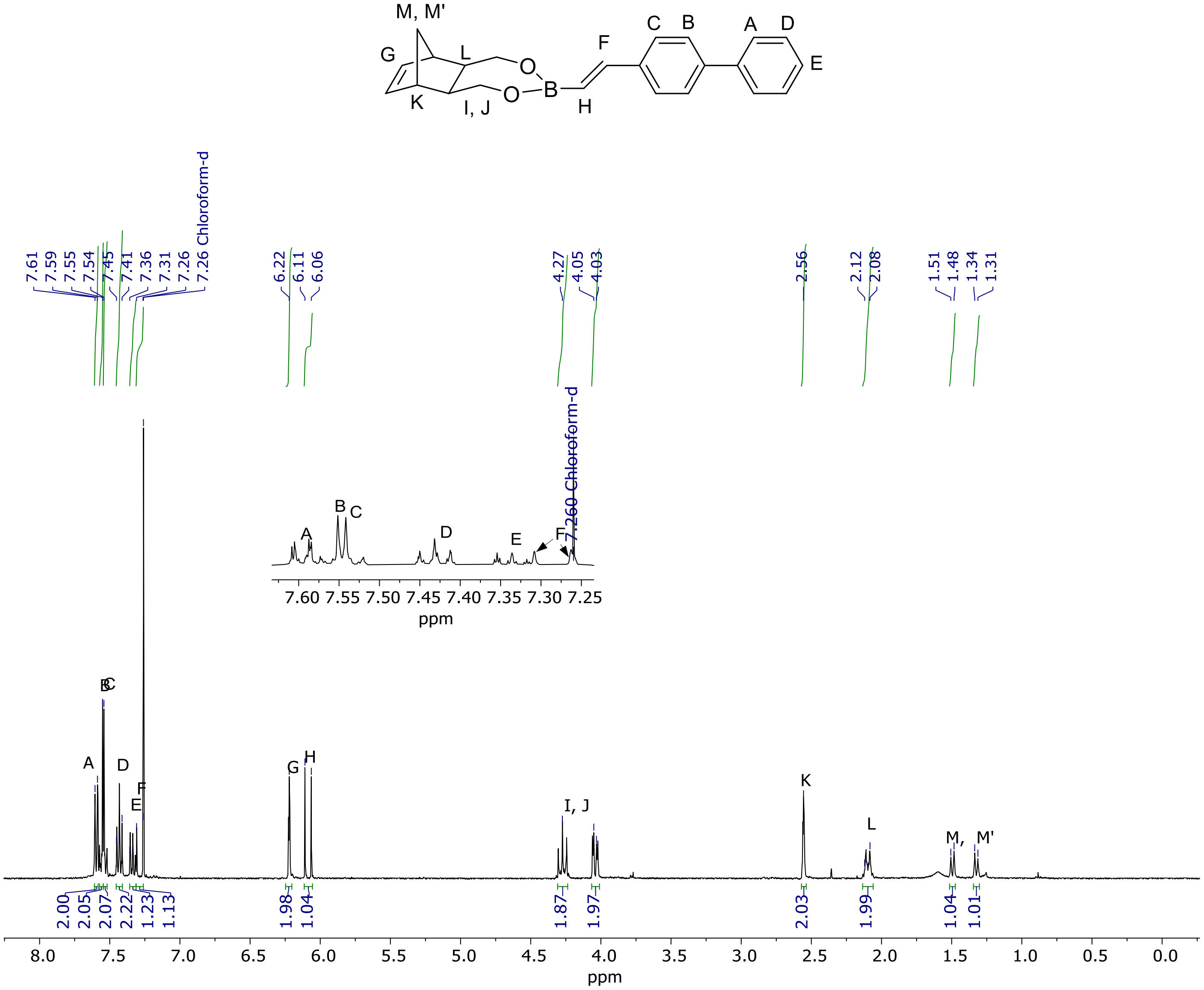

Figure S31. Compound M7 – ^{1}H NMR in $CDCl_3$

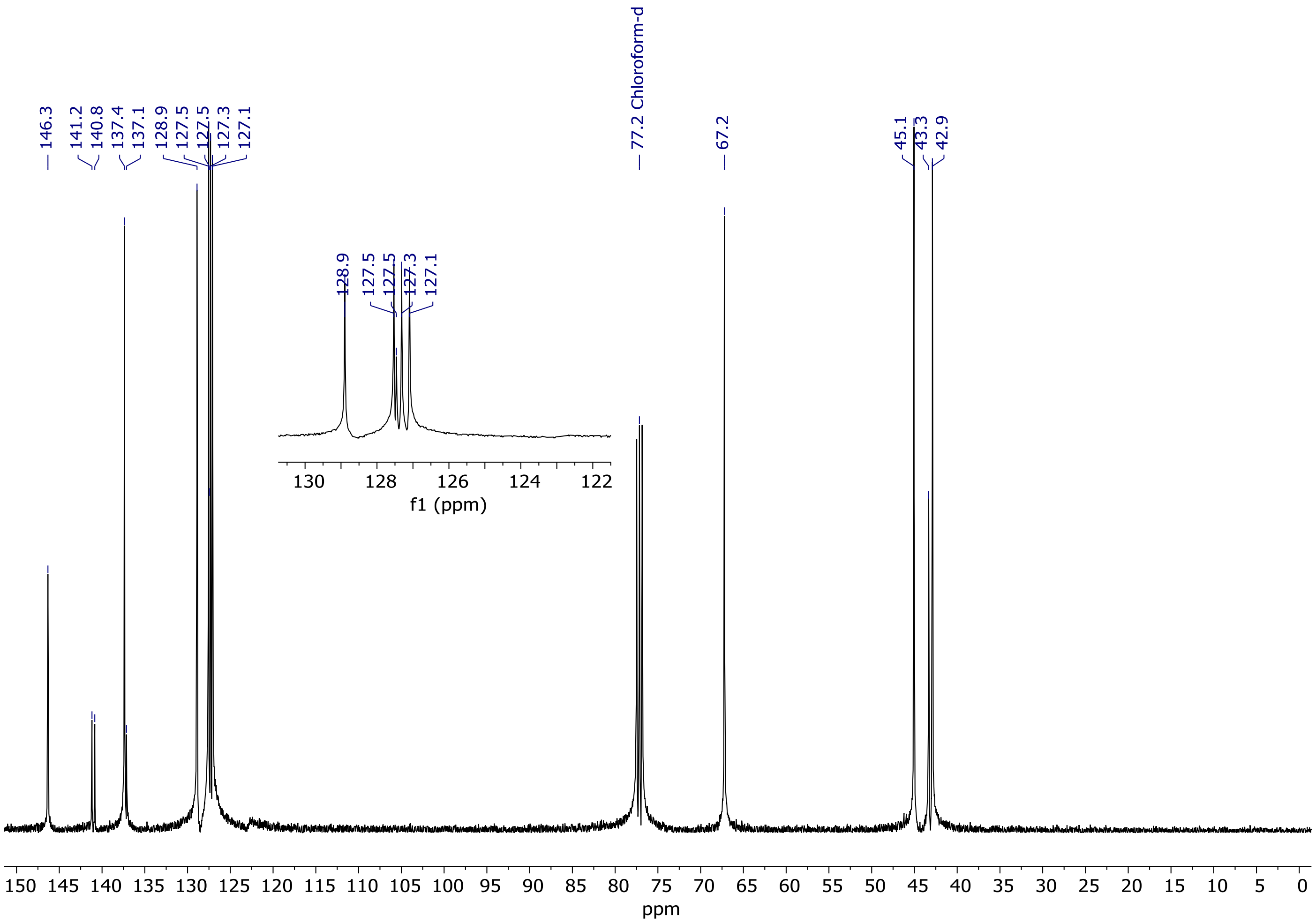

Figure S32. Compound M7 – ^{13}C NMR in $CDCl_3$

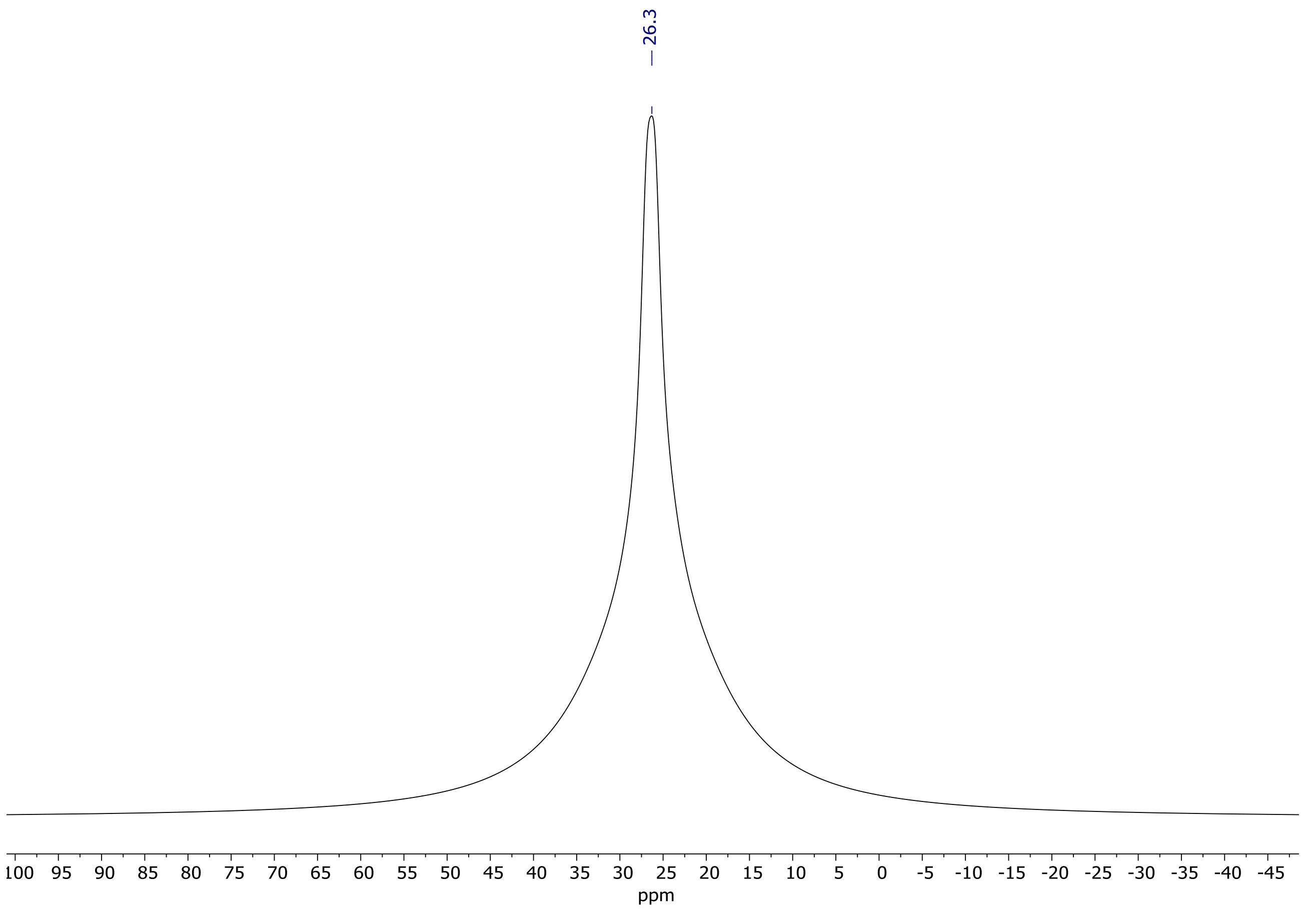

Figure S33. Compound M7 - ^{11}B NMR in $CDCl_3$

Polymers

Compound P1/P1a

Poly(5-norbornene-2-*exo*,3-*exo*-dimethyl-[(phenyl)-dioxaborepine])s – G1

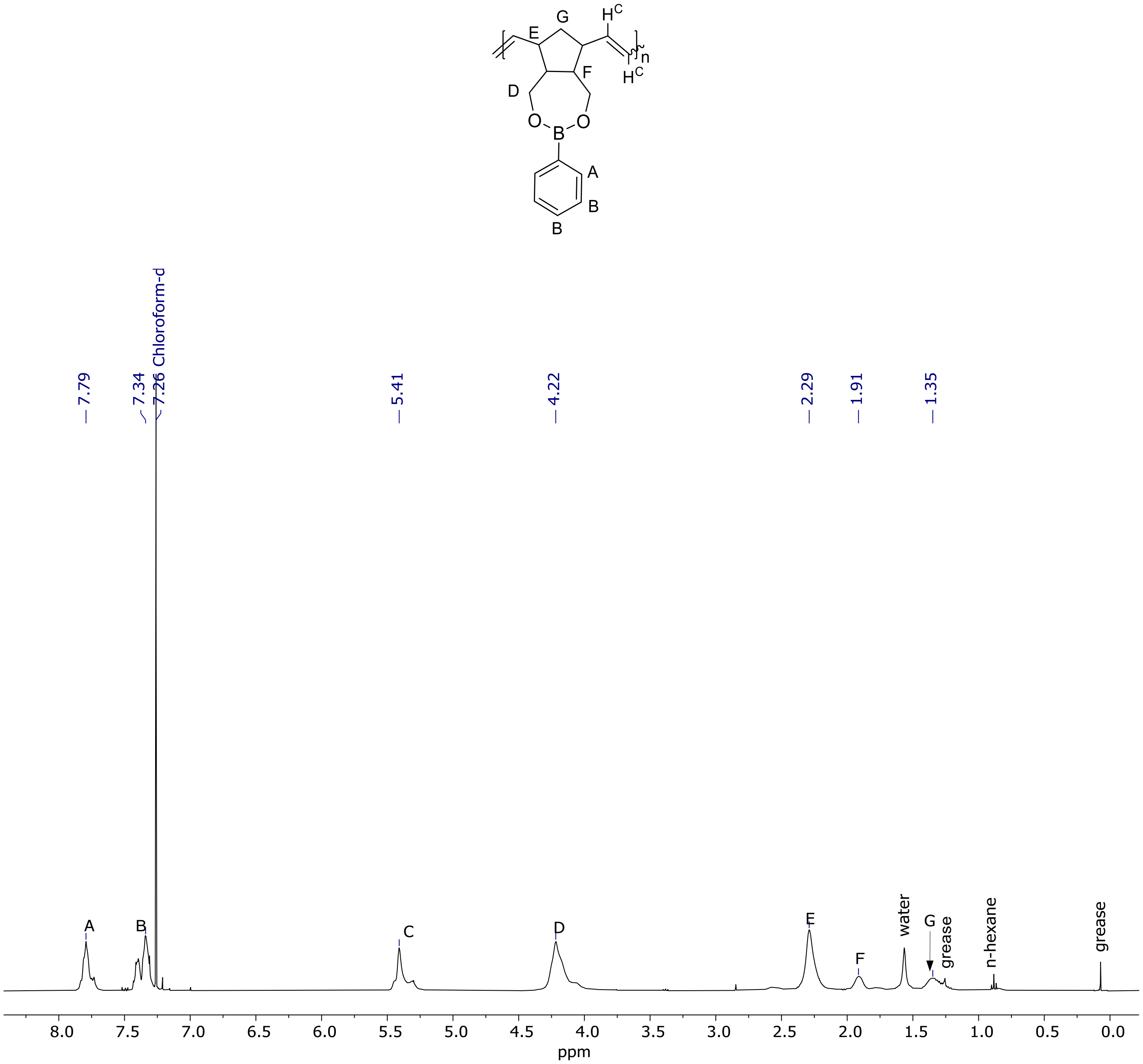

Figure S34. Compound P1/P1a – ^{1}H NMR in $CDCl_3$

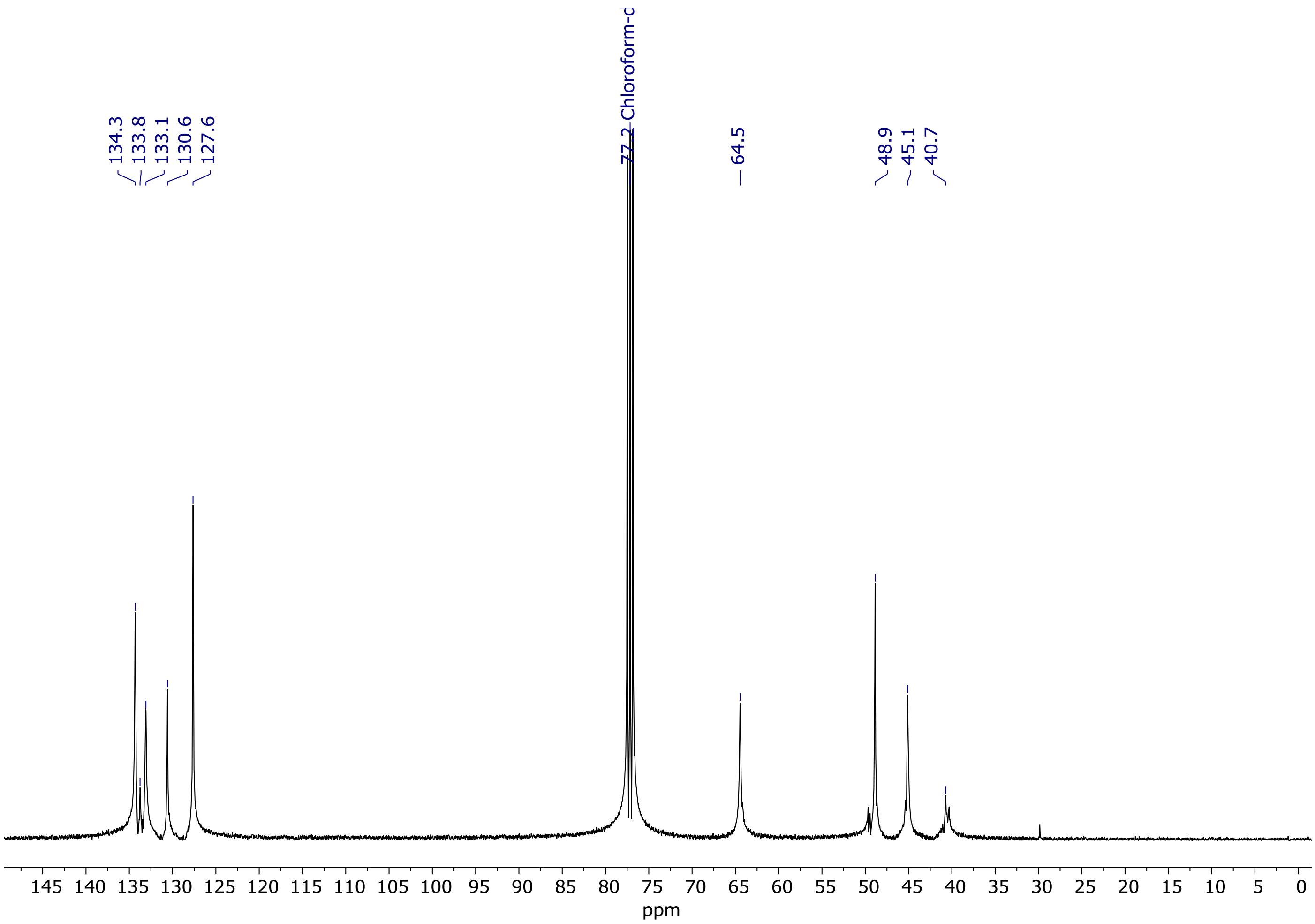

Figure S35. Compound P1/P1a – ^{13}C NMR in $CDCl_3$

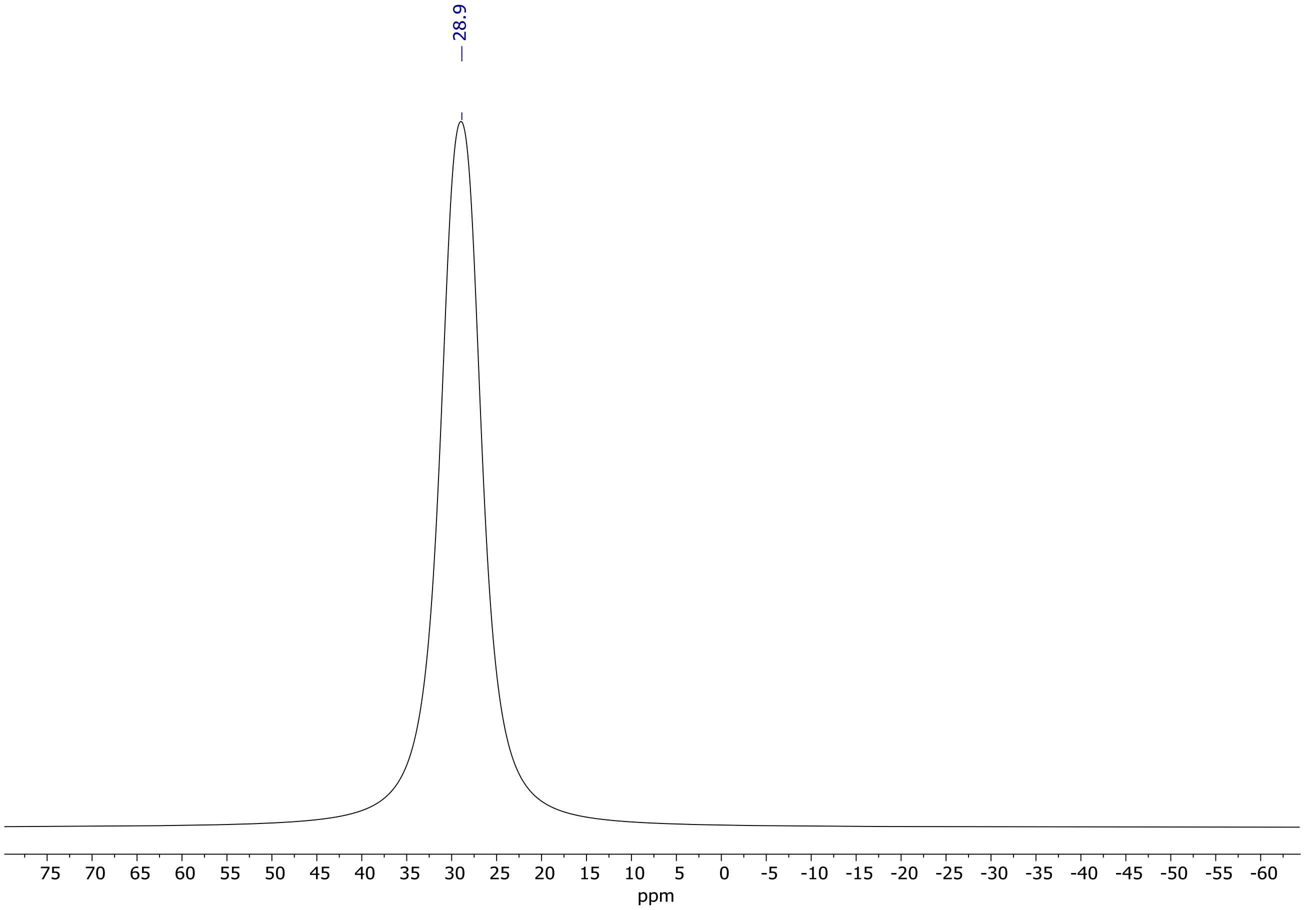

Figure S36. Compound P1/P1a – ^{11}B NMR in $CDCl_3$

Compound P1b

Poly(5-norbornene-2-*exo*,3-*exo*-dimethyl-[(phenyl)-dioxaborepine])s – G2

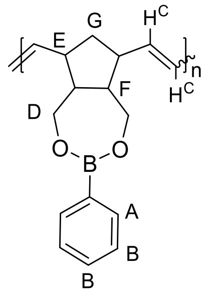

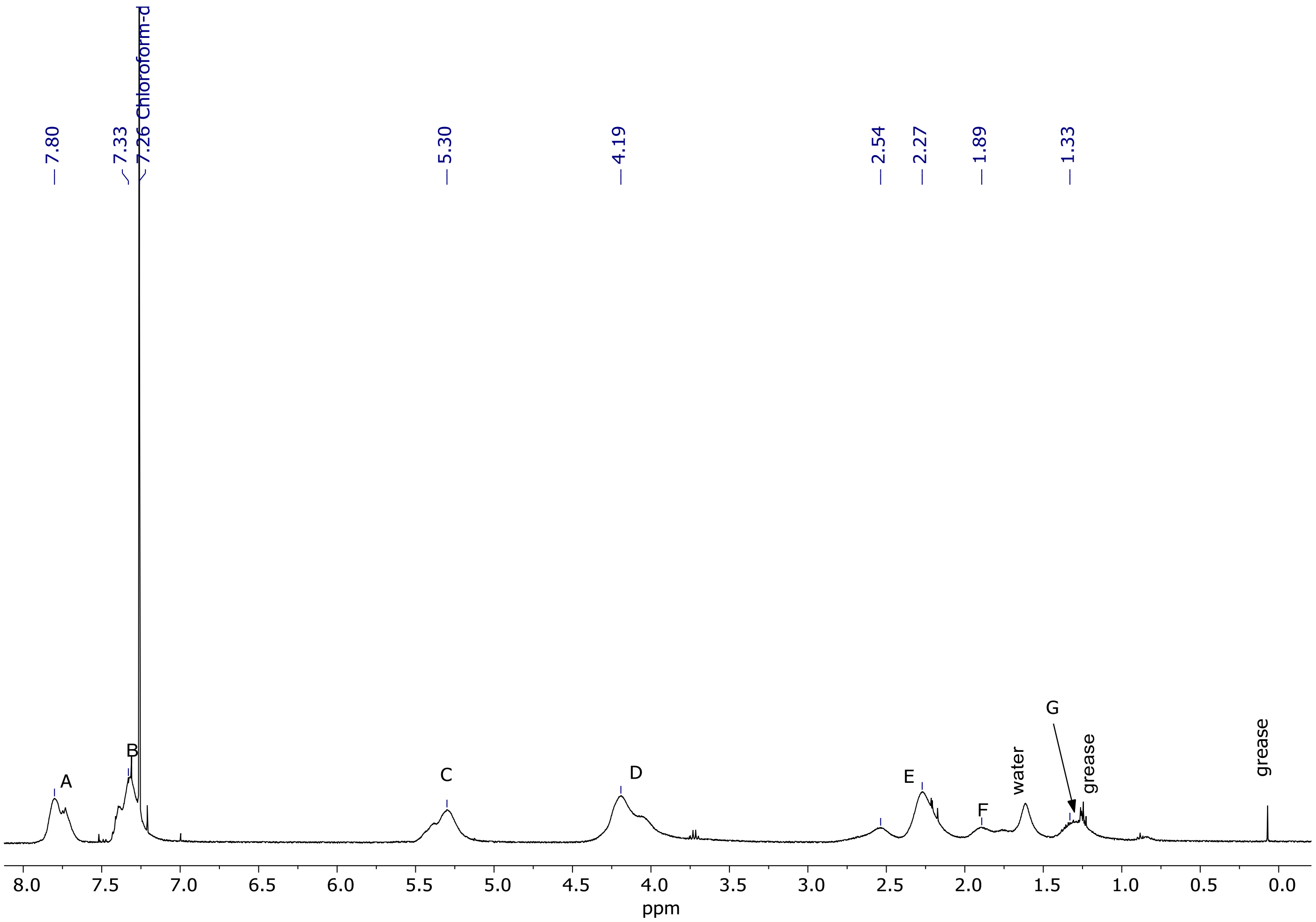

Figure S37. Compound P1b – ^{1}H NMR in $CDCl_3$

Compound P3

Poly(5-norbornene-2-*exo*,3-*exo*-dimethyl-[(9-phenantracenyl)-dioxaborepine])s

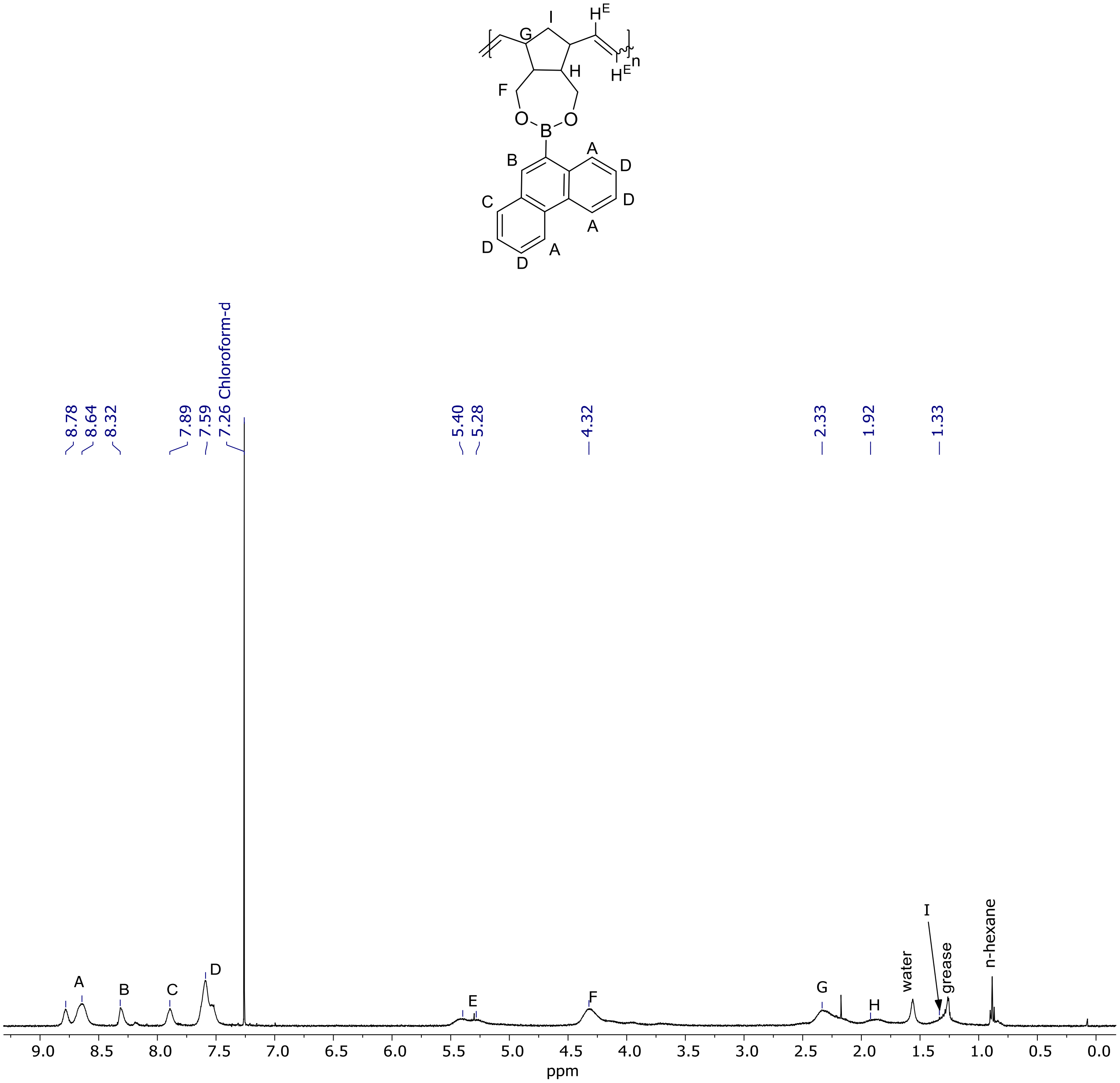

Figure S38. Compound P3 – ^{1}H NMR in $CDCl_3$

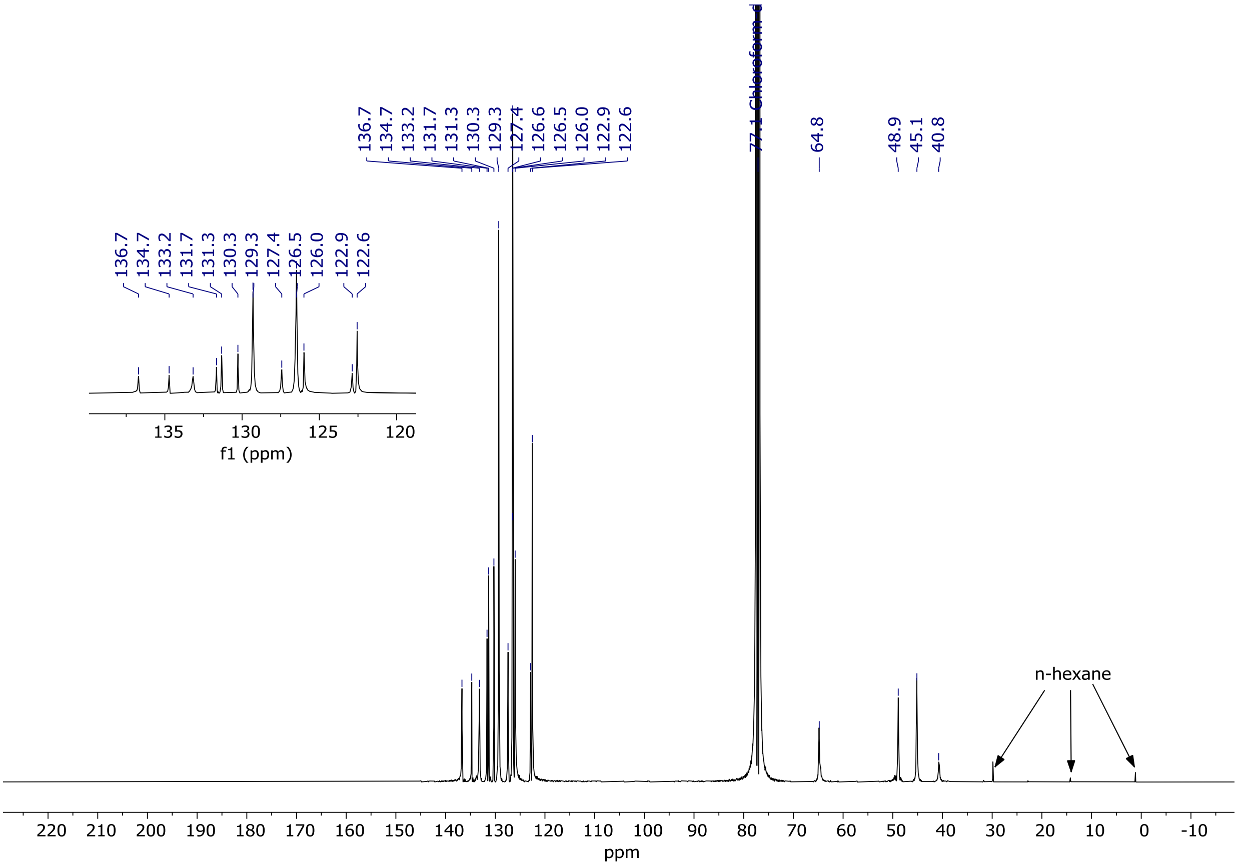

Figure S39. Compound P3 – ^{13}C NMR in $CDCl_3$

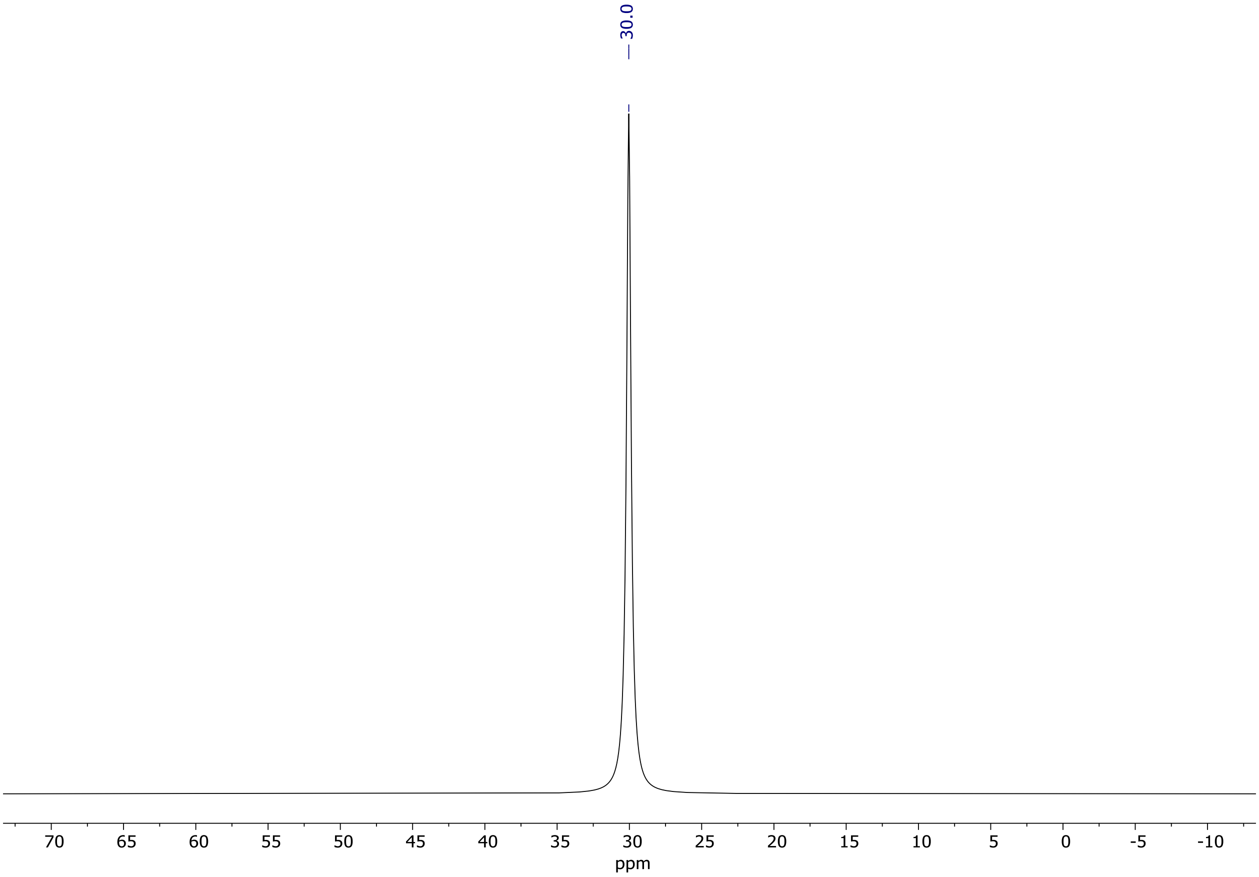

Figure S40. Compound P3 – ^{11}B NMR in $CDCl_3$

Compound P5

Poly(5-norbornene-2-*exo*,3-*exo*-dimethyl-[(3,5-dibromophenyl)-dioxaborepine])s

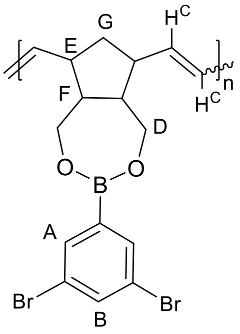

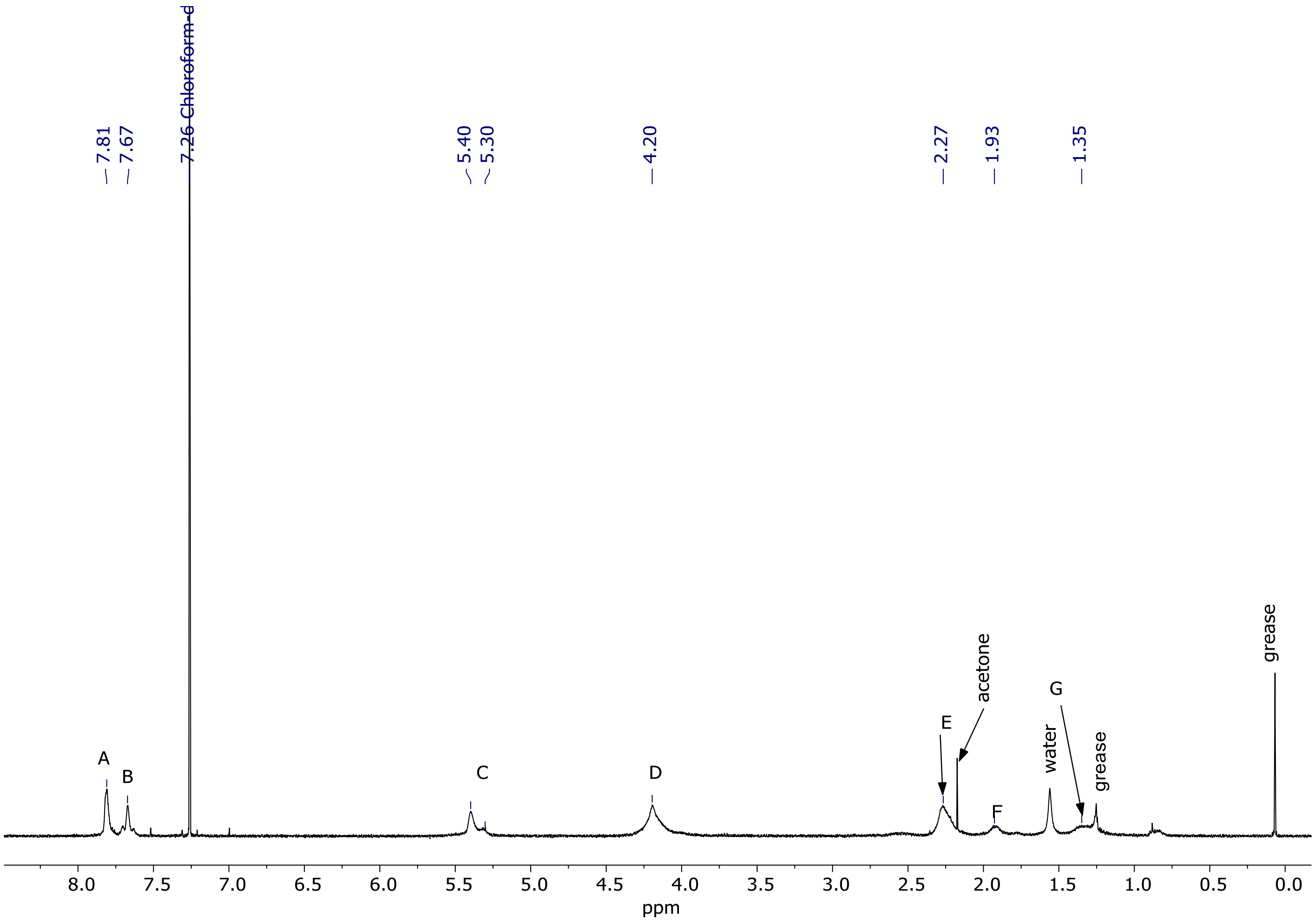

Figure S41. Compound P5 – ^{1}H NMR in $CDCl_3$

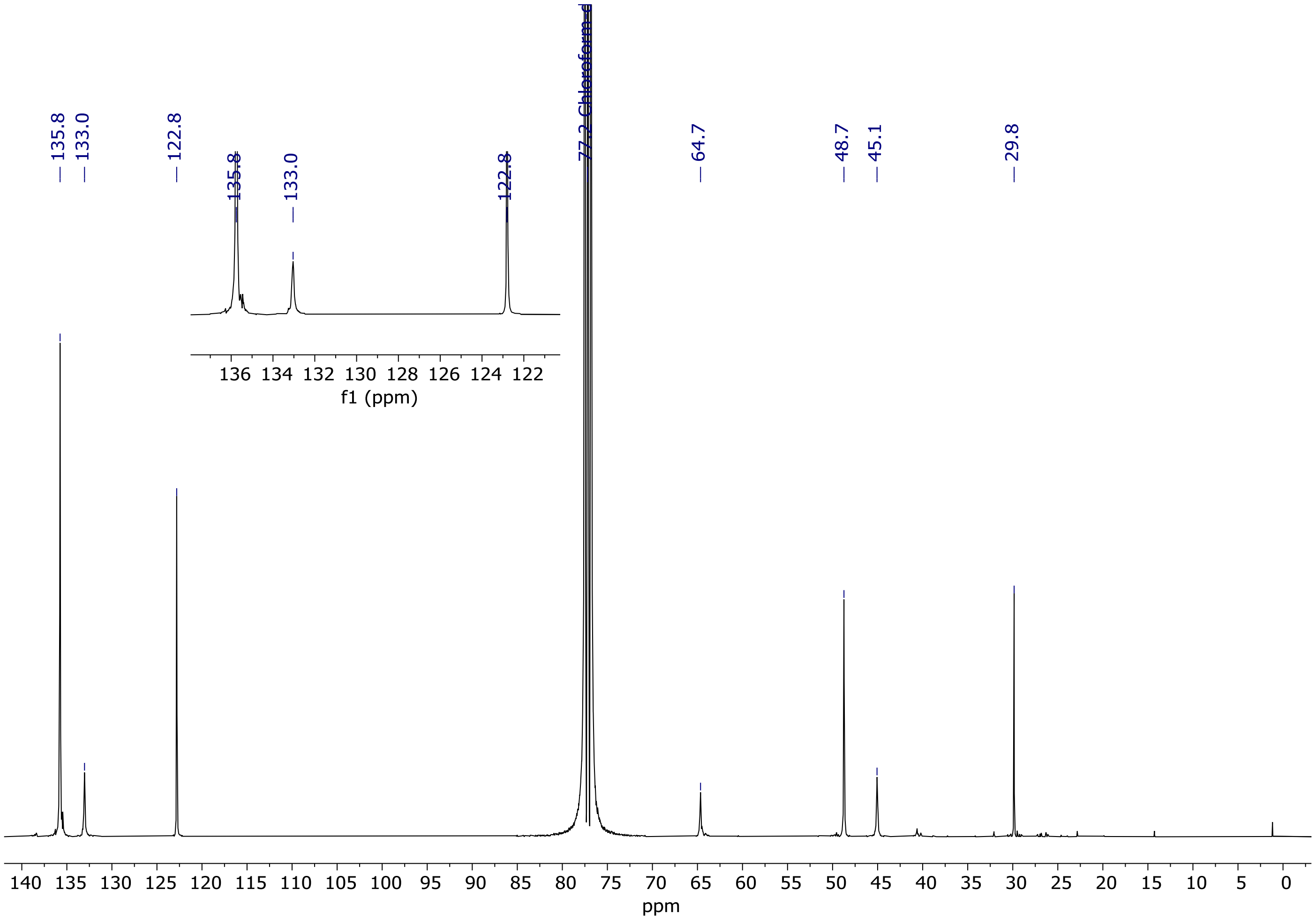

Figure S42. Compound P5 – ^{13}C NMR in $CDCl_3$

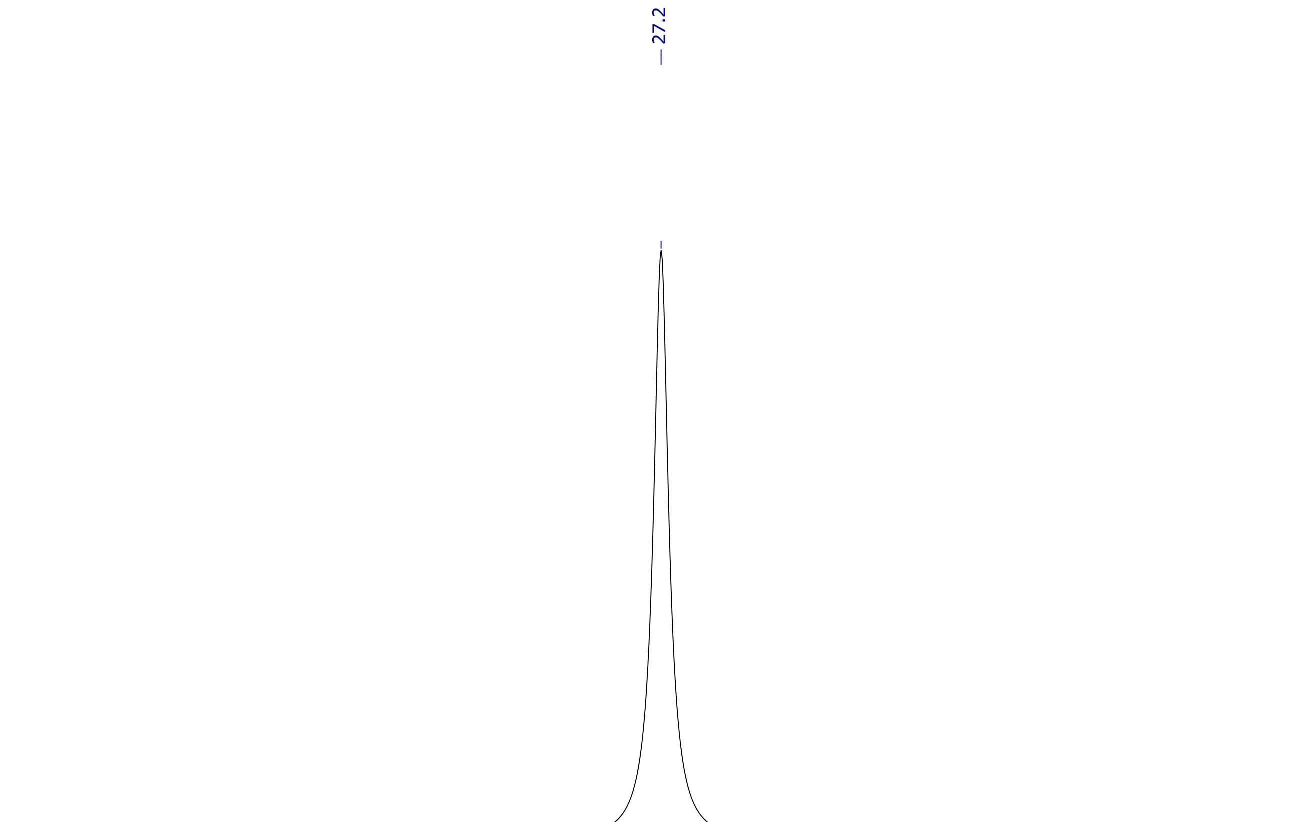

Figure S43. Compound P5 – ^{11}B NMR in $CDCl_3$

Compound P6

Poly(5-norbornene-2-*exo*,3-*exo*-dimethyl-[(trans-2-(4-methylphenyl)vinyl)-dioxaborepine])s

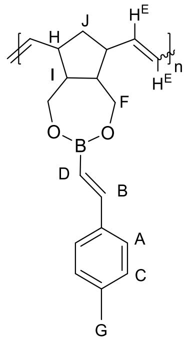

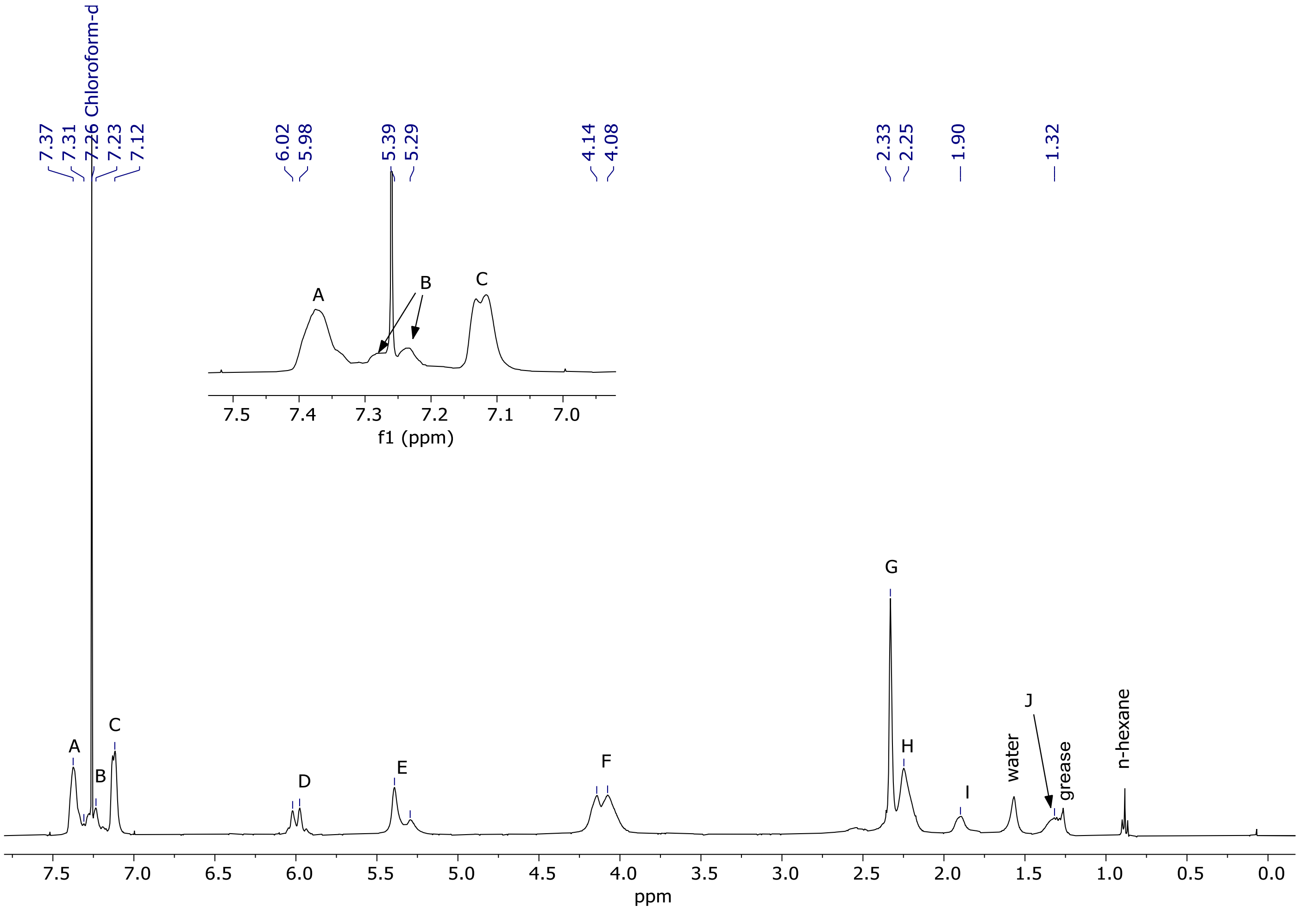

Figure S44. Compound P6 – ^{1}H NMR in $CDCl_3$

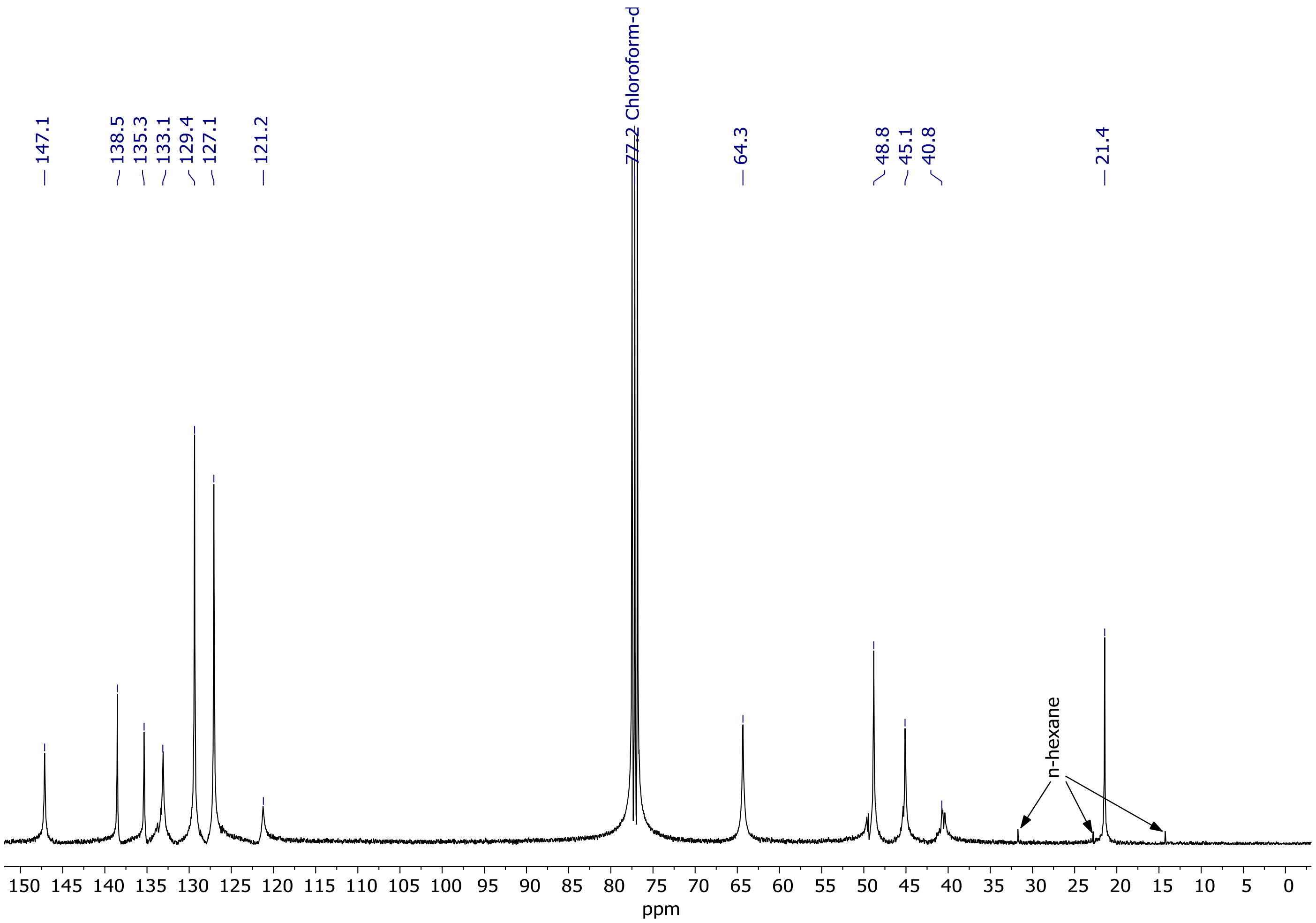

Figure S45. Compound P6 – ^{13}C NMR in $CDCl_3$

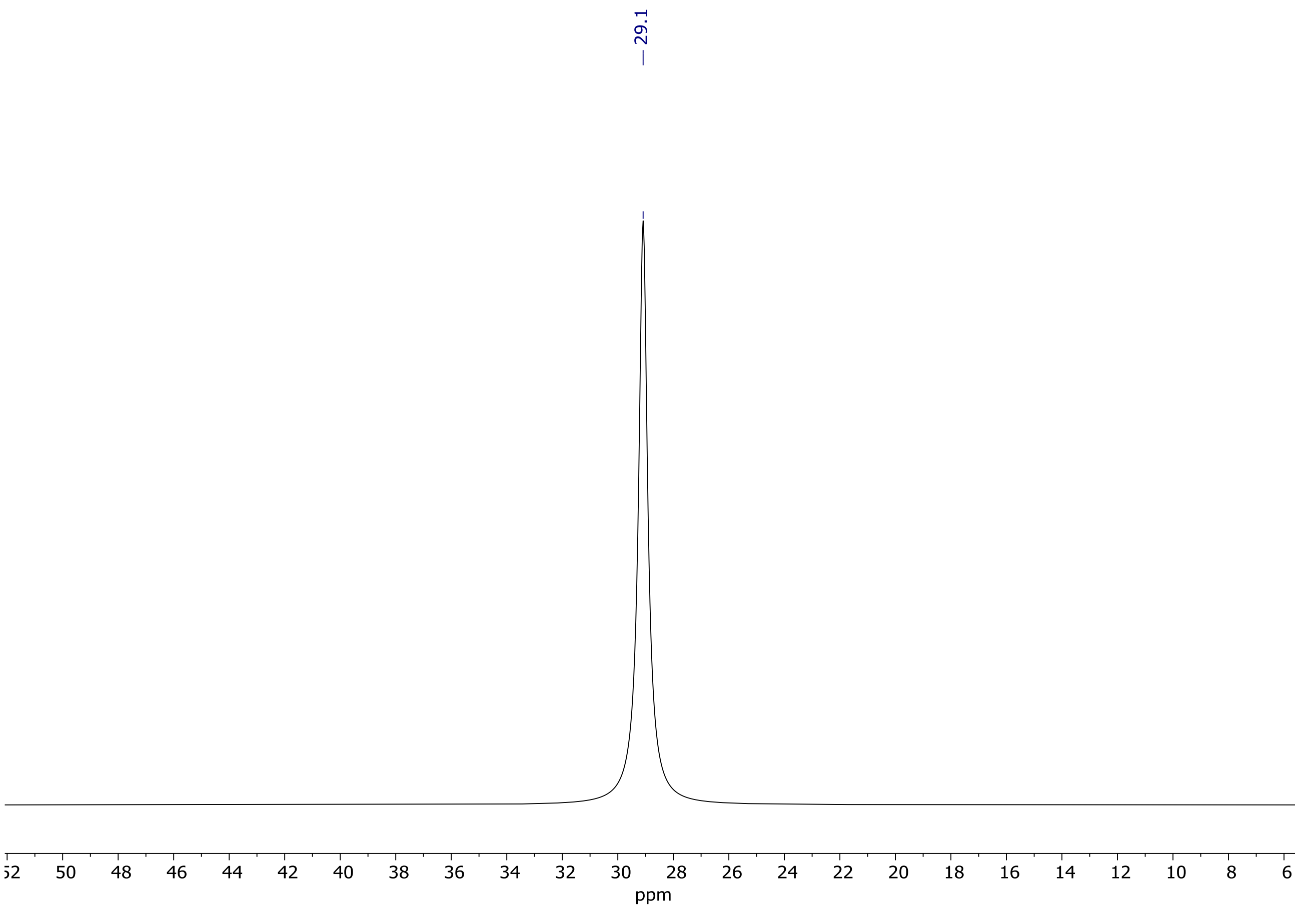

Figure S46. Compound P6 – ^{11}B NMR in $CDCl_3$

Compound P7

Poly(5-norbornene-2-*exo*,3-*exo*-dimethyl-[(trans-2-(4-biphenyl)vinyl)-dioxaborepine])s

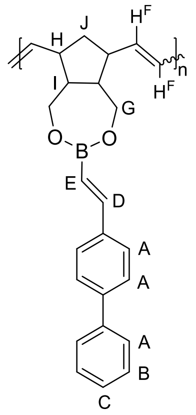

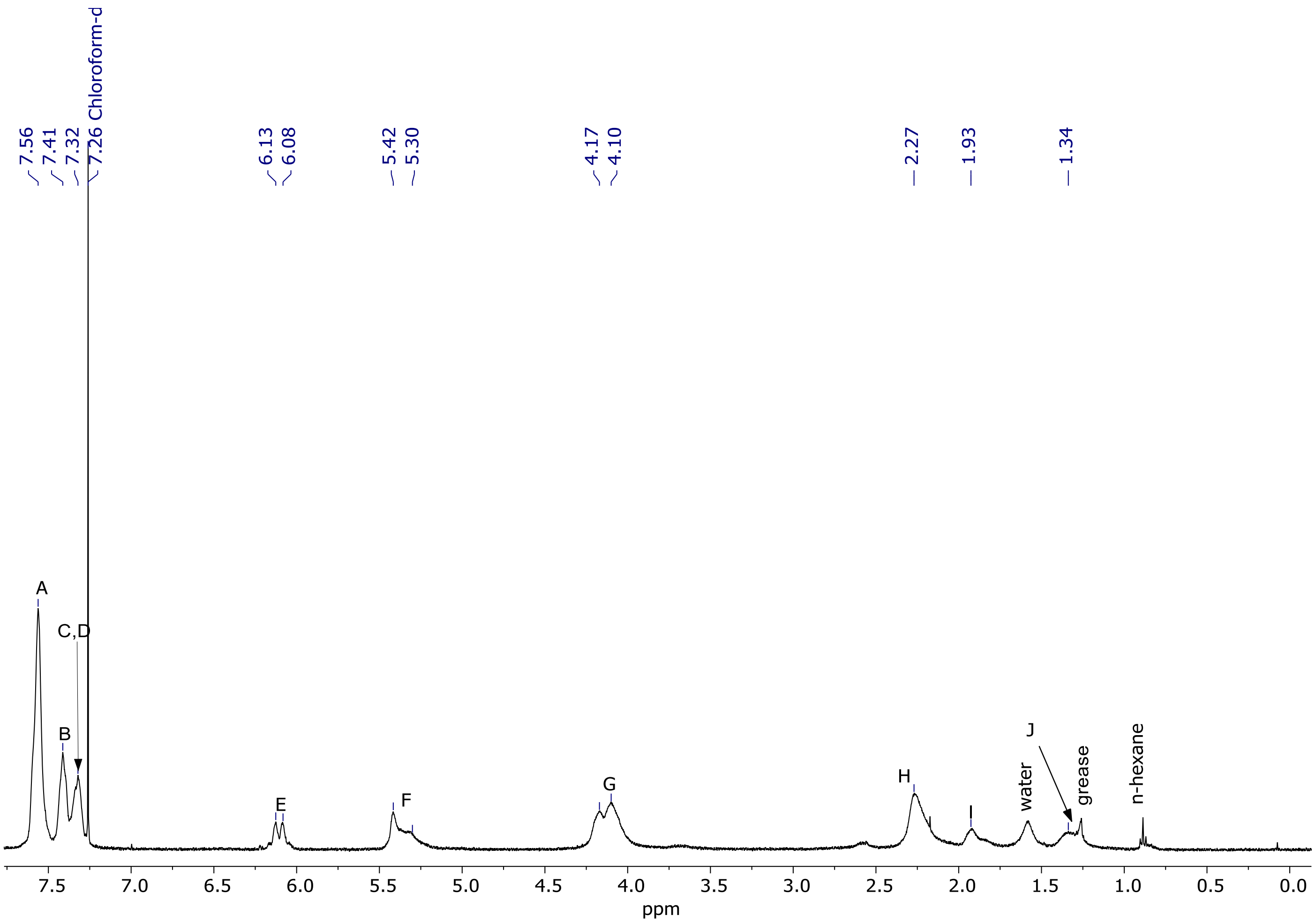

Figure S47. Compound P7 – ^{1}H NMR in $CDCl_3$

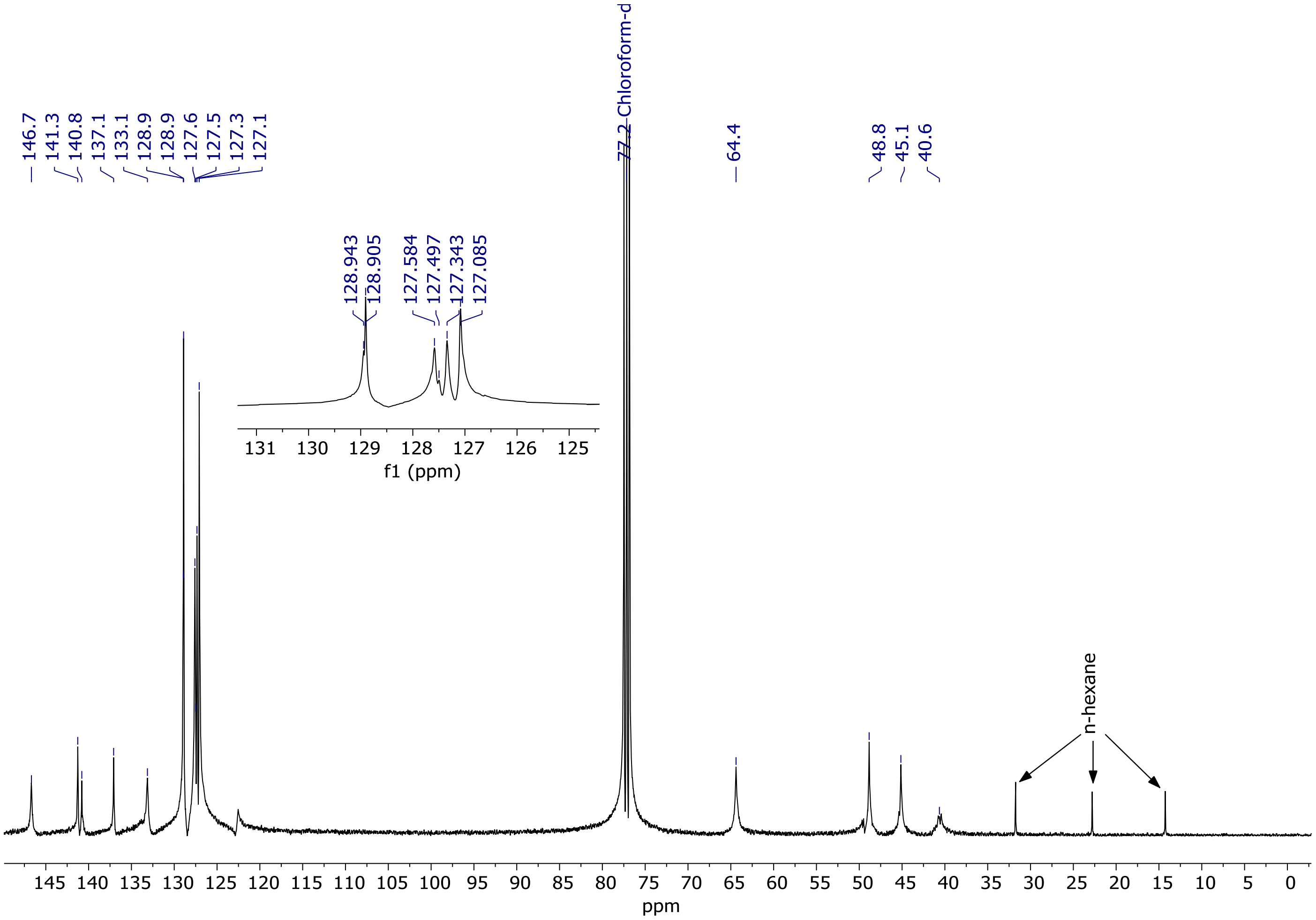

Figure S48. Compound P7 – ^{13}C NMR in $CDCl_3$

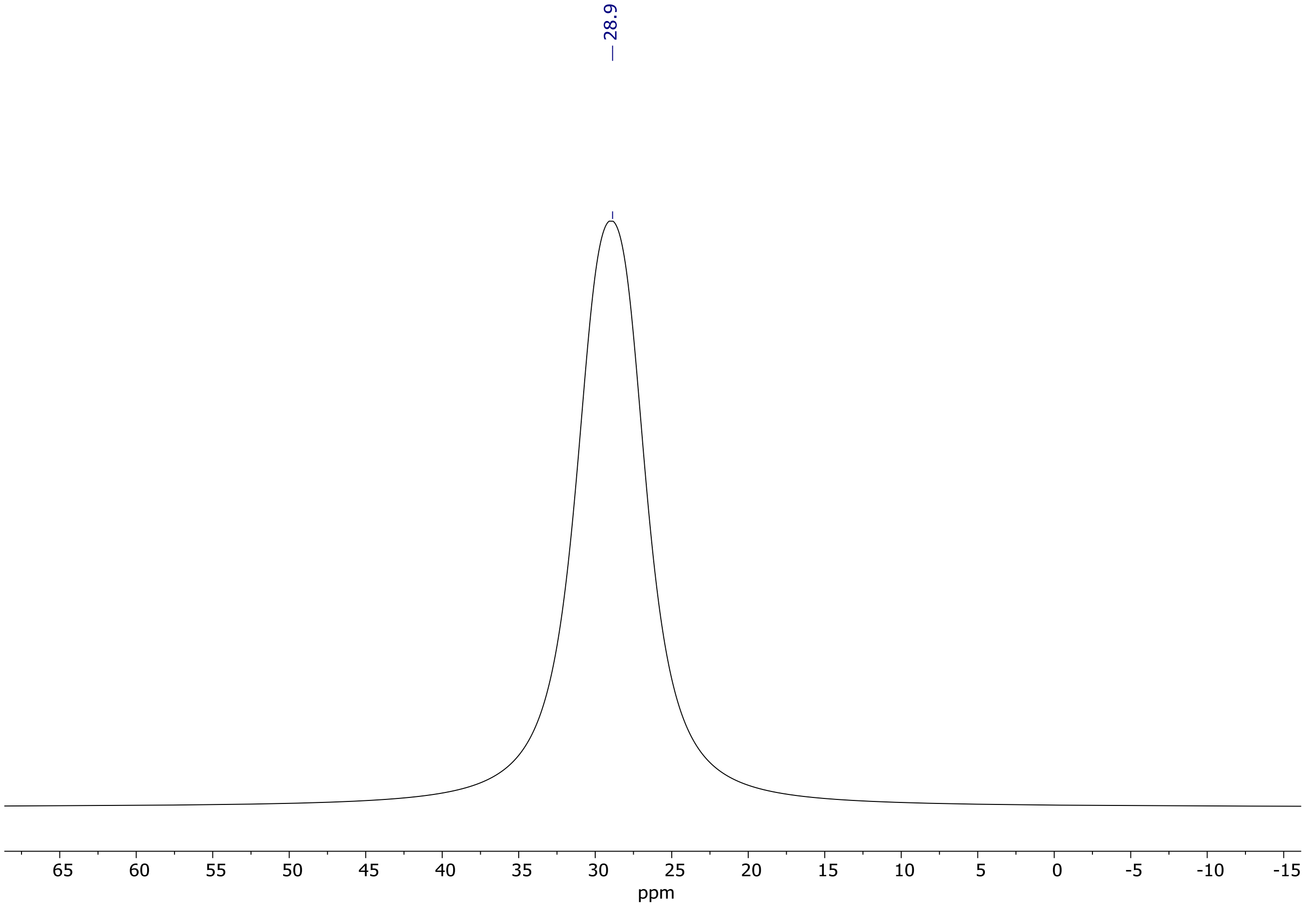

Figure S49. Compound P7 – ^{11}B NMR in $CDCl_3$

Coordination studies

Compound C1

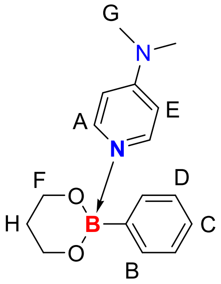

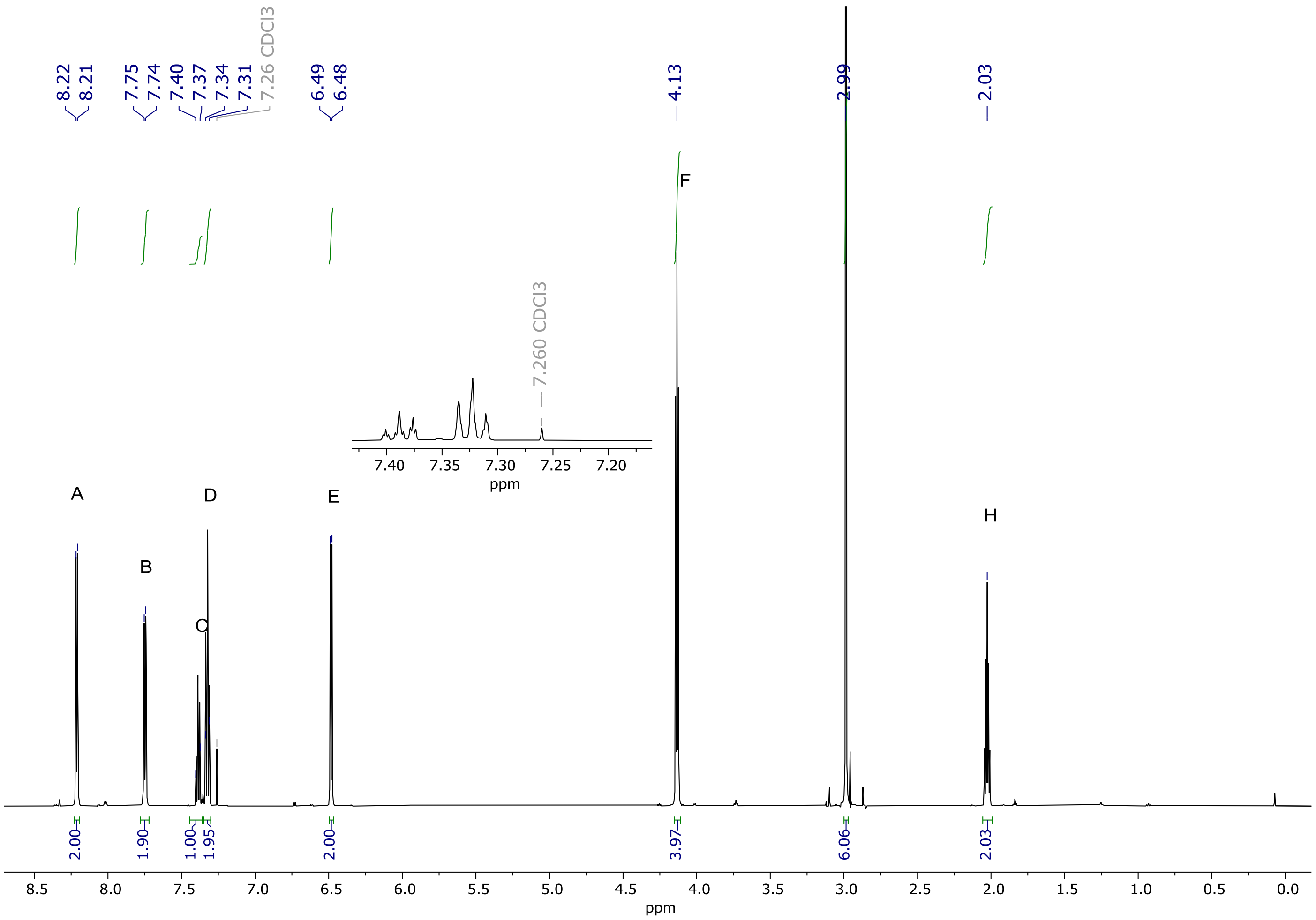

Figure S50. Compound C1 – ^{1}H NMR in $CDCl_3$

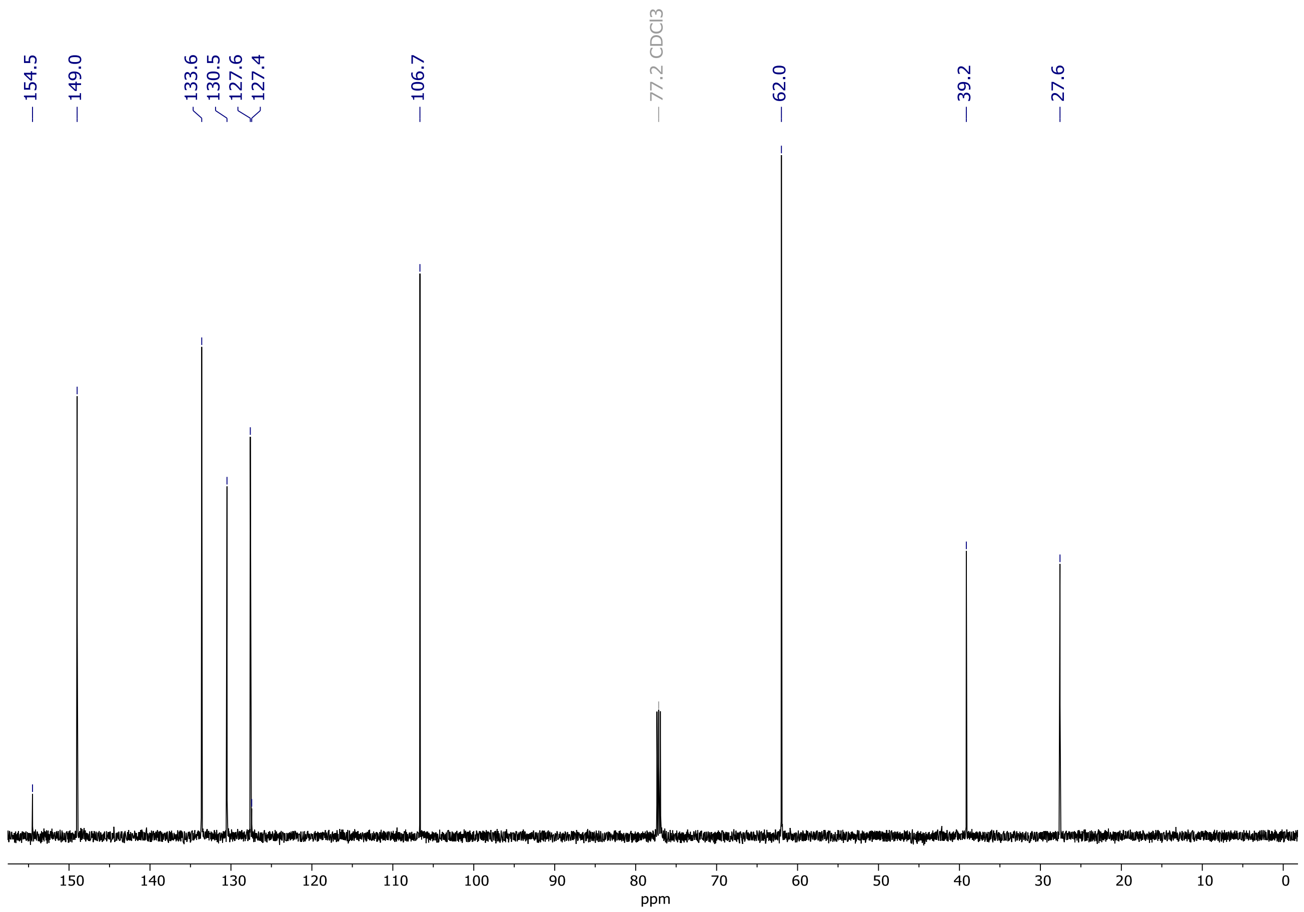

Figure S51. Compound C1 – ^{13}C NMR in $CDCl_3$

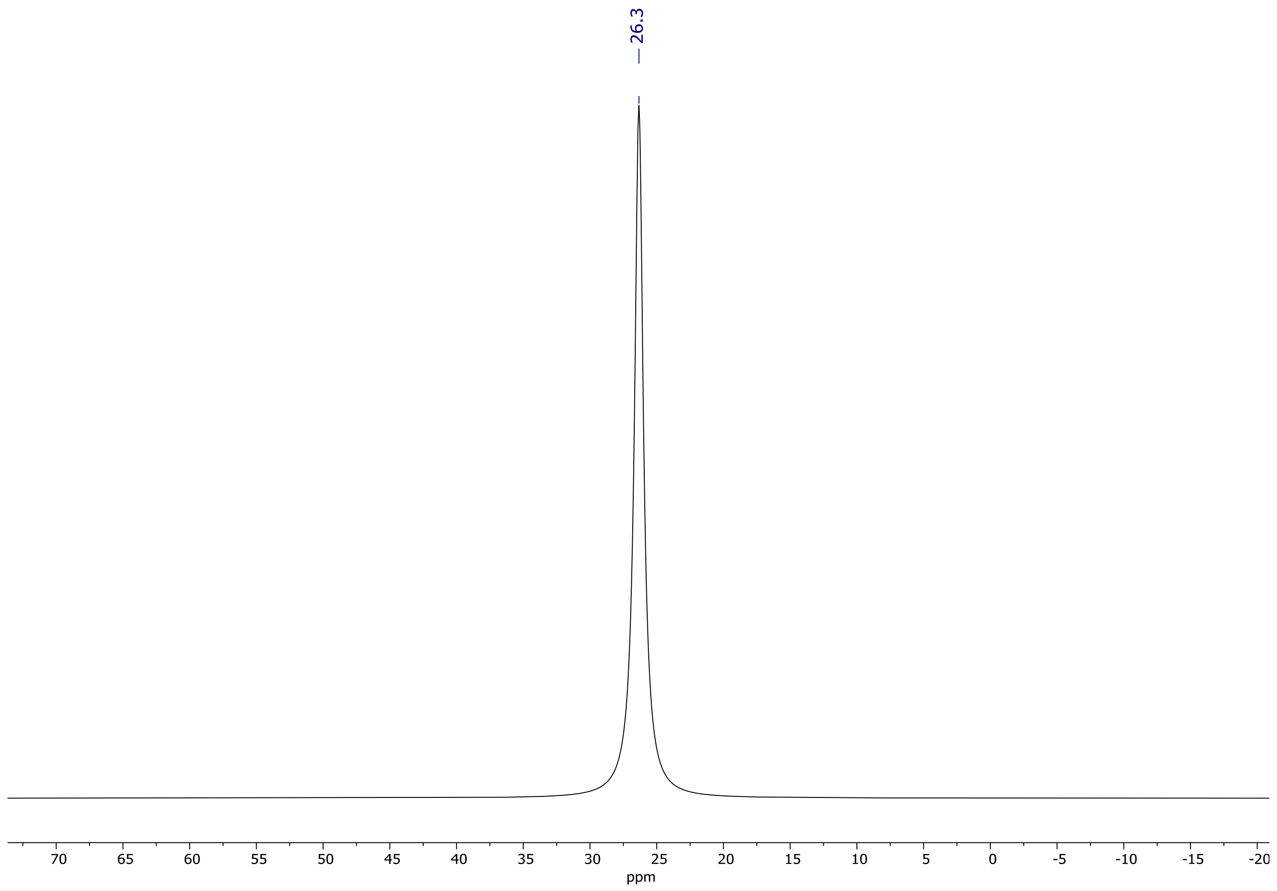

Figure S52. Compound C1 – ^{11}B NMR in $CDCl_3$

Compound C2

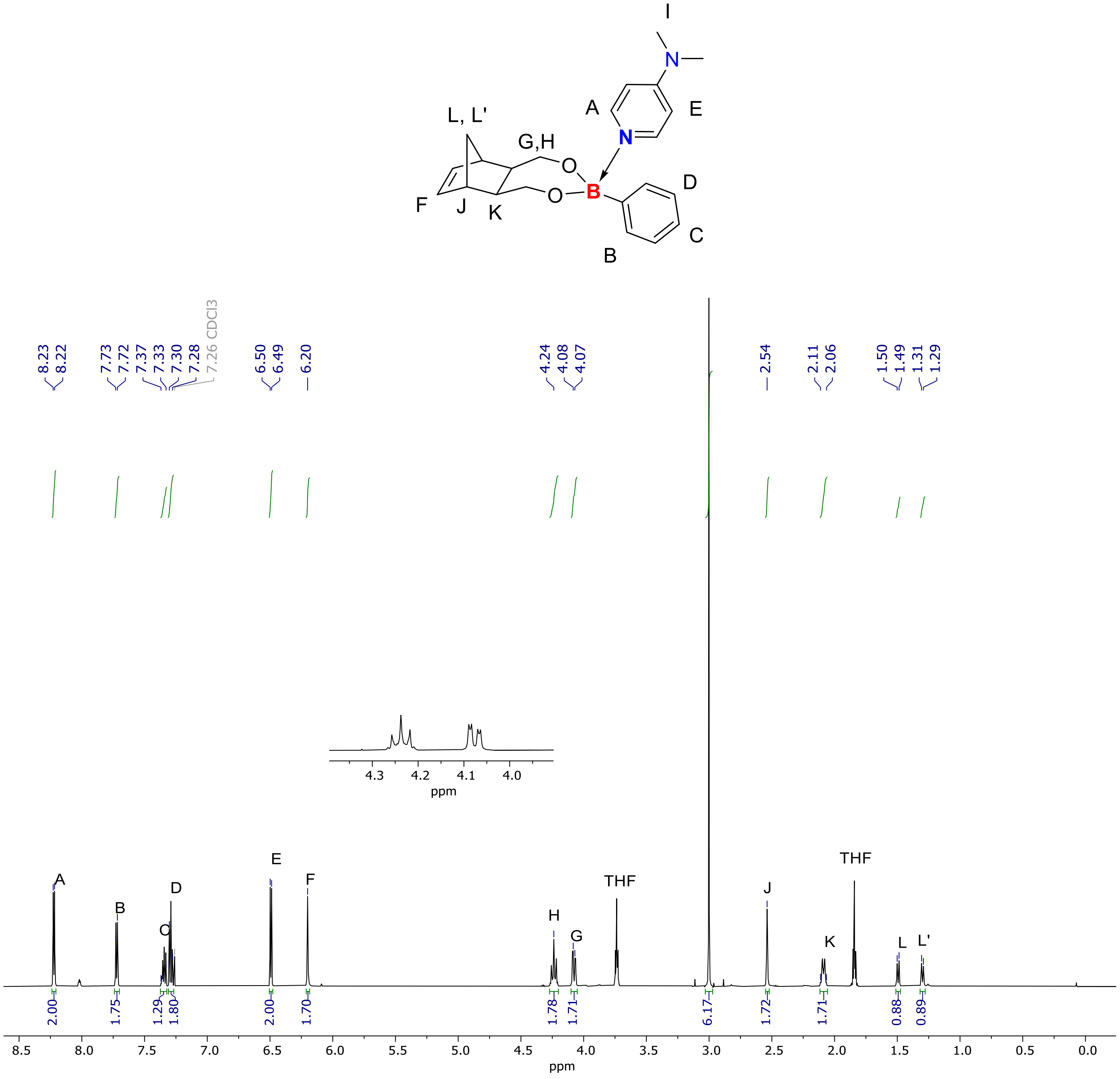

Figure S53. Compound C2 – ^{1}H NMR in $CDCl_3$

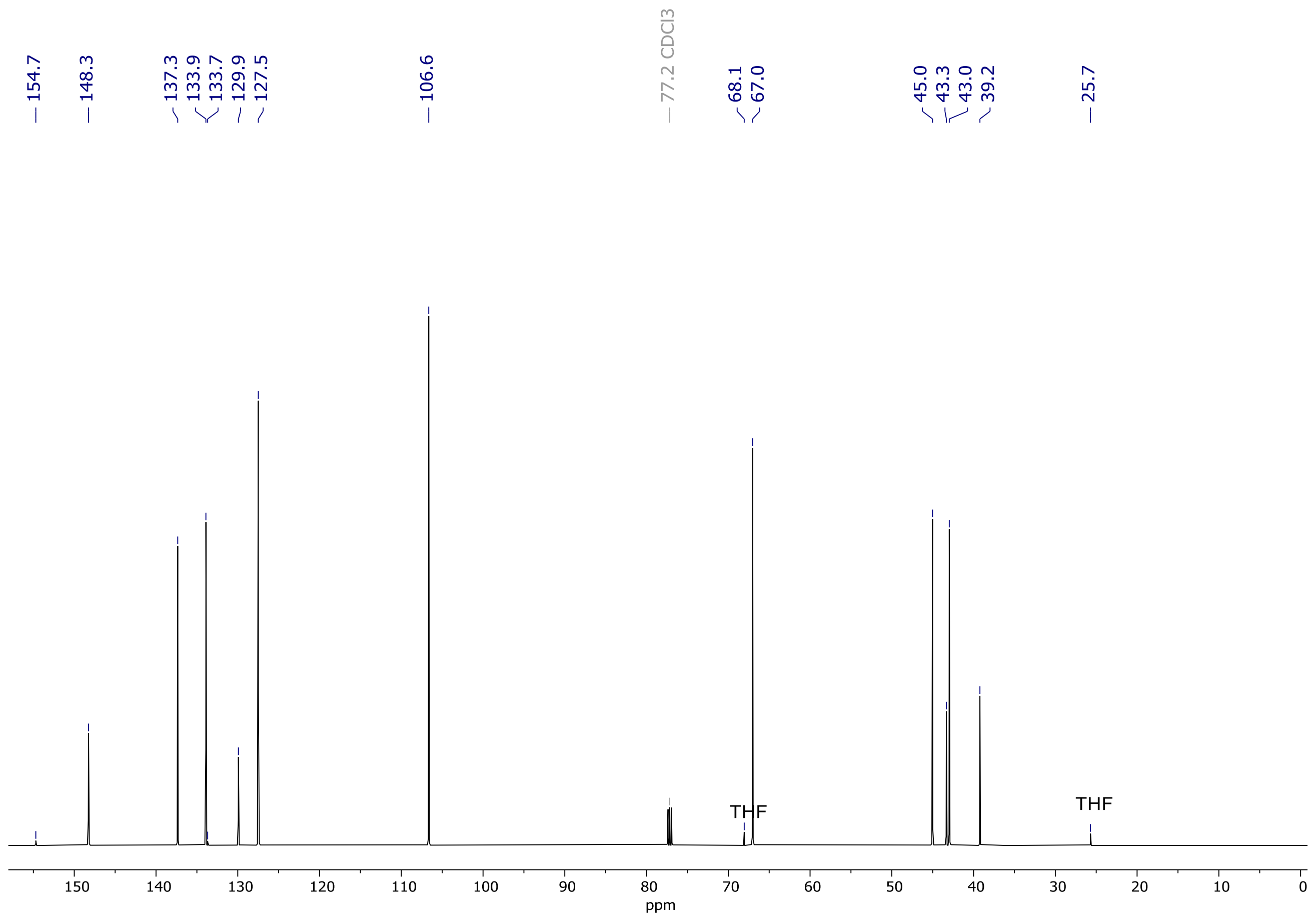

Figure S54. Compound C2 – ^{13}C NMR in $CDCl_3$

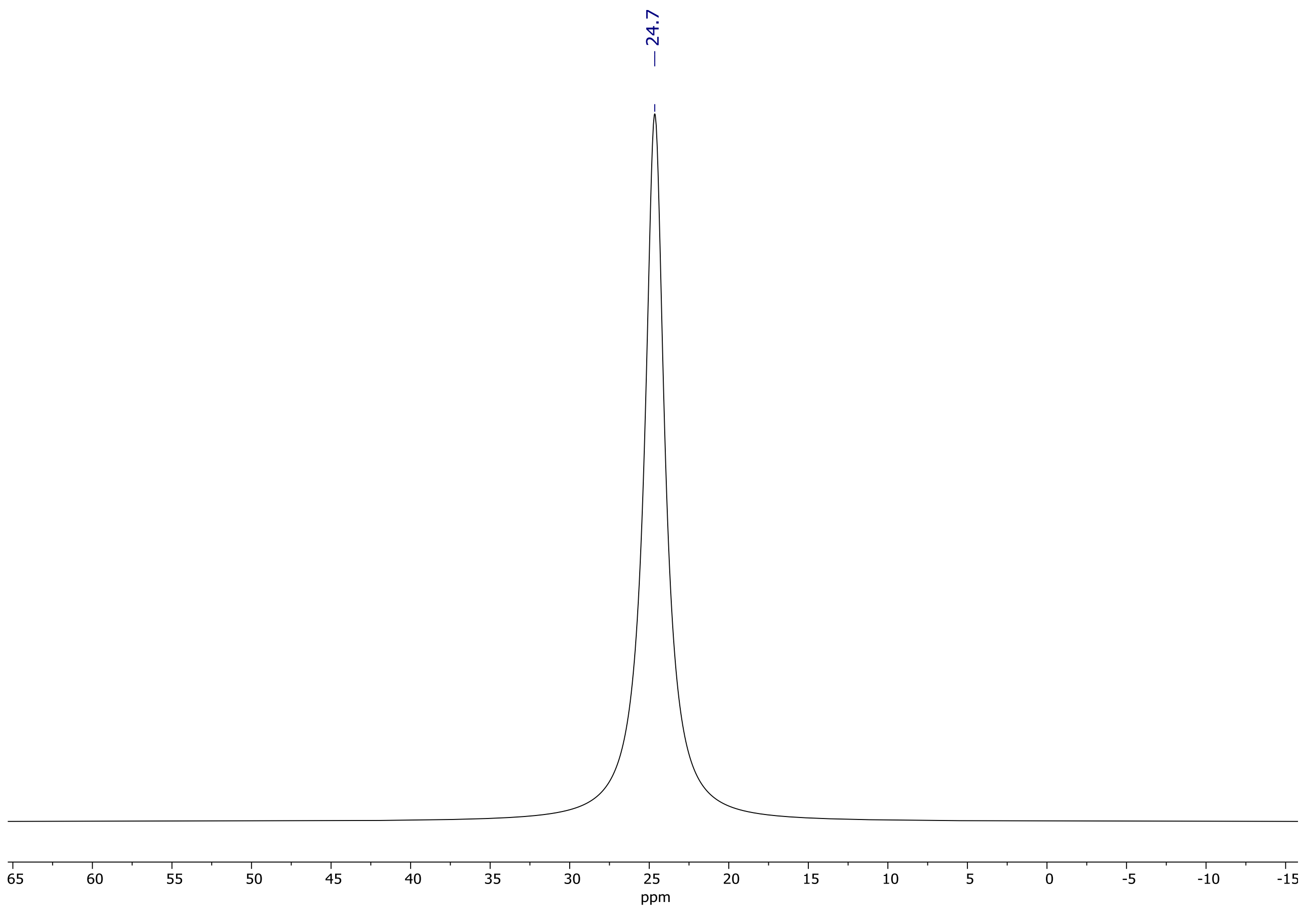

Figure S55. Compound C2 – ^{11}B NMR in $CDCl_3$

Compound C3

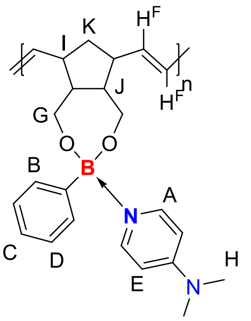

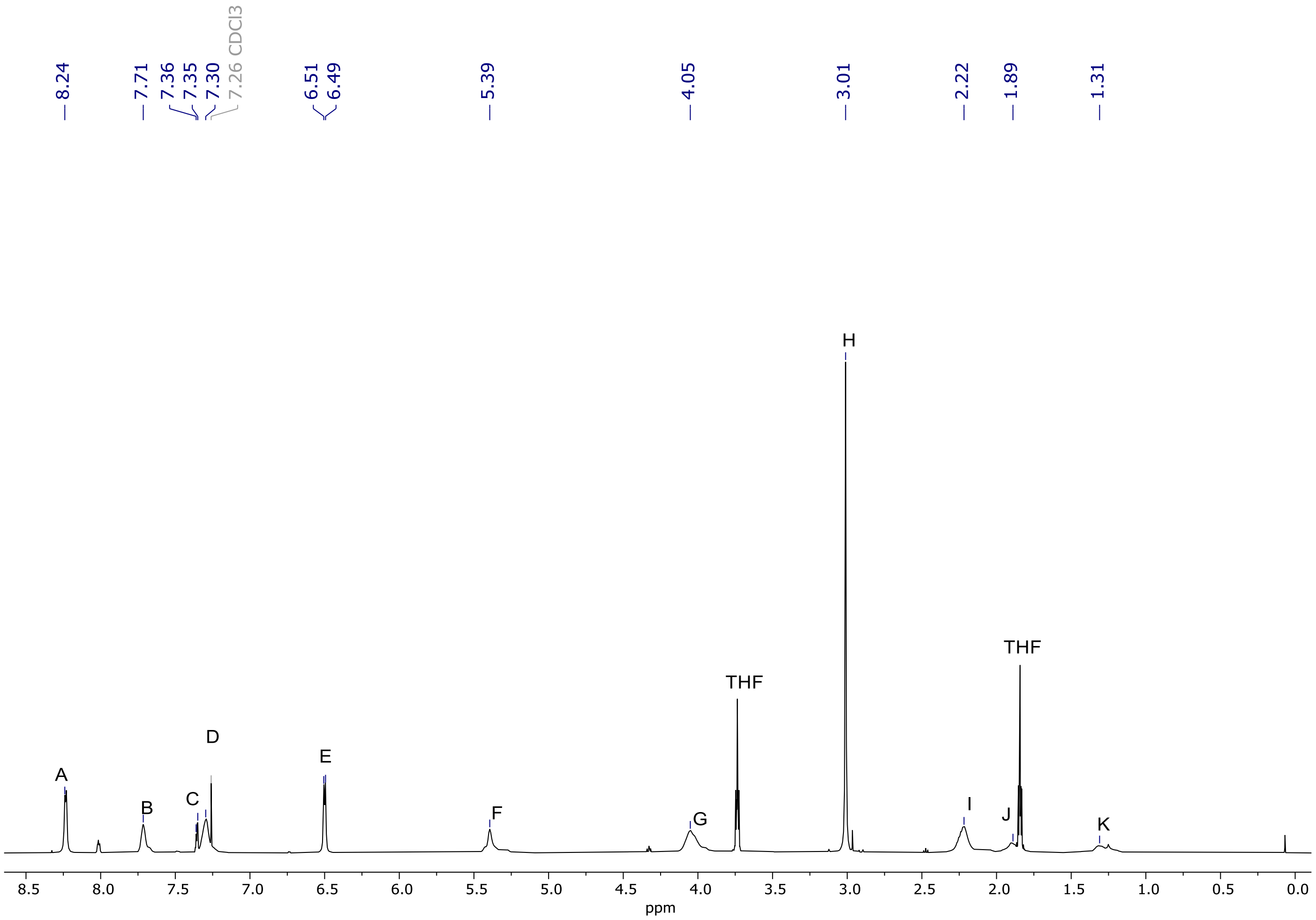

Figure S56. Compound C3 – ^{1}H NMR in $CDCl_3$

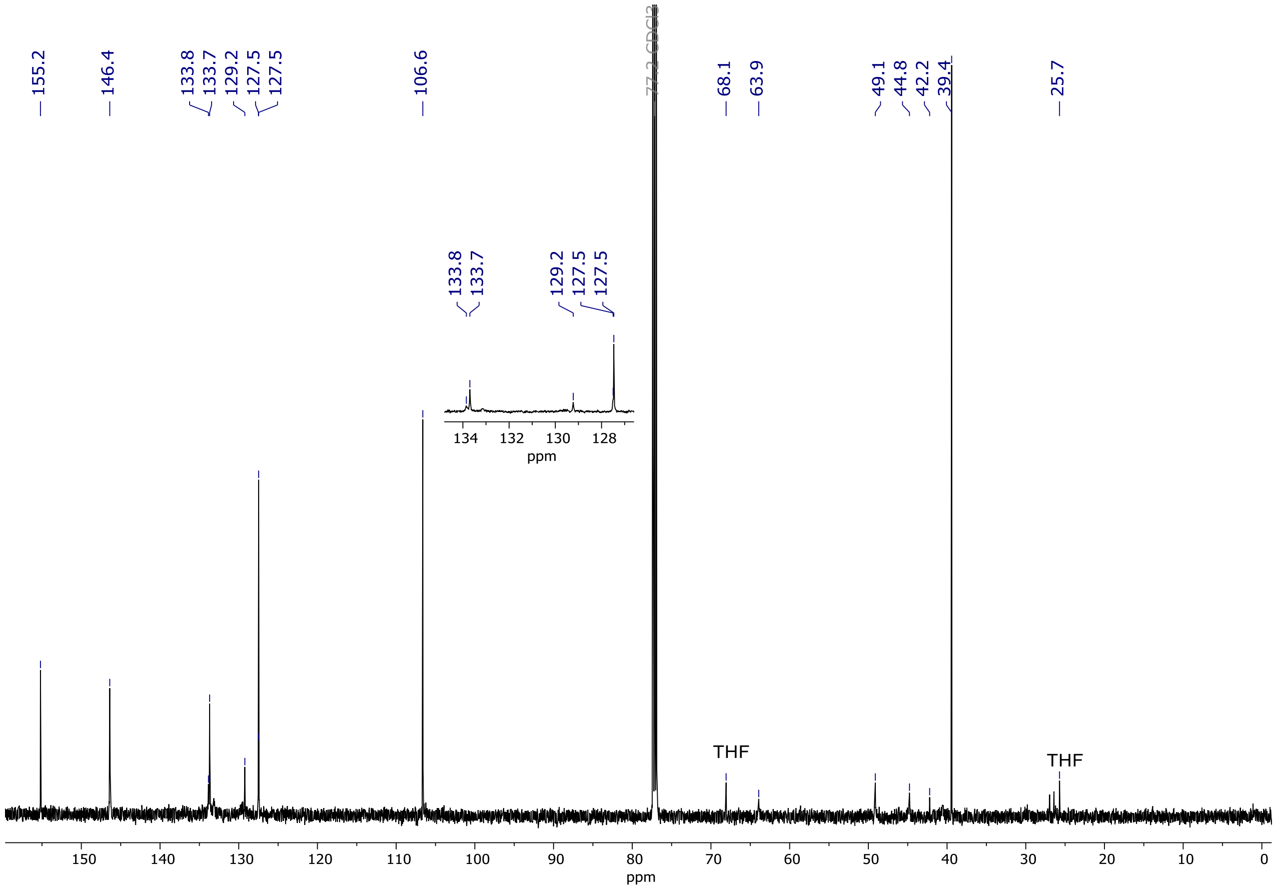

Figure S57. Compound C3 – ^{13}C NMR in $CDCl_3$

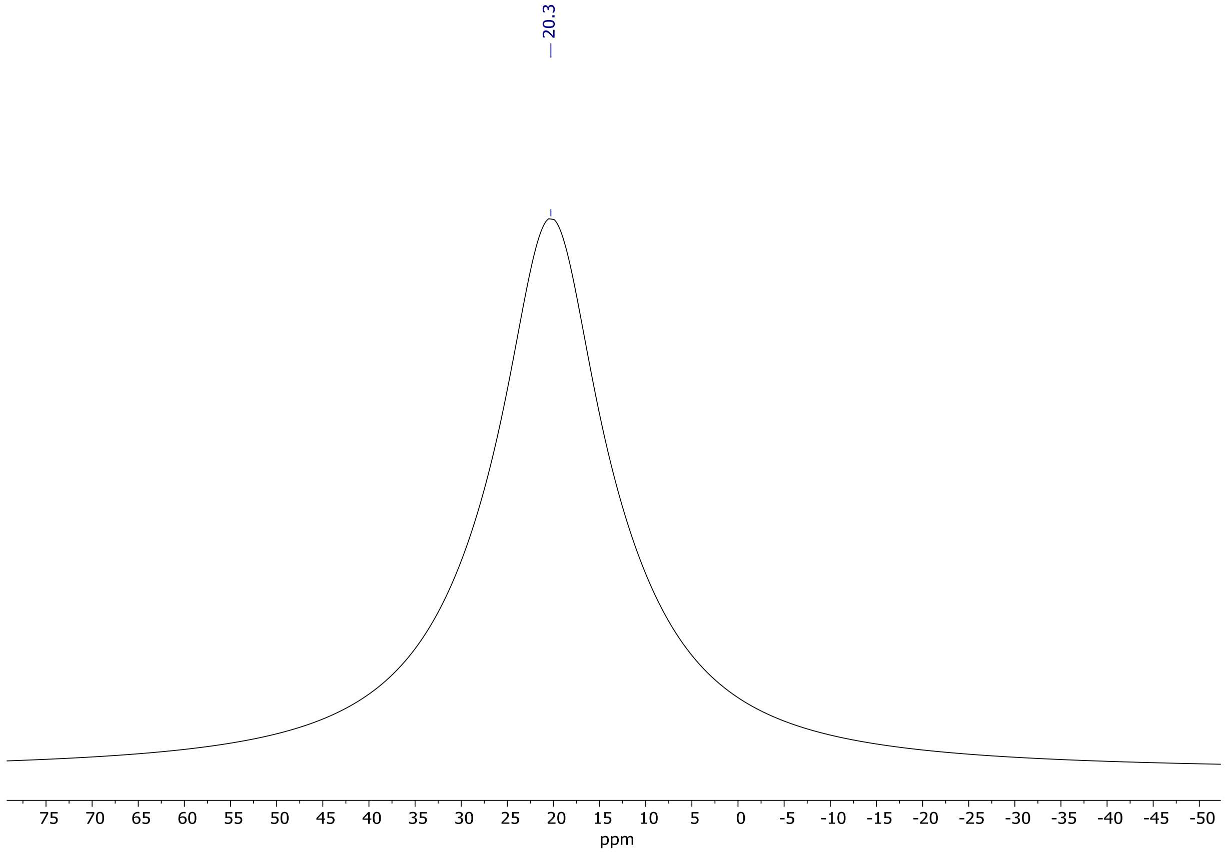

Figure S58. Compound C3 – ^{11}B NMR in $CDCl_3$

Gel permeation chromatography (GPC) analysis (blue line).

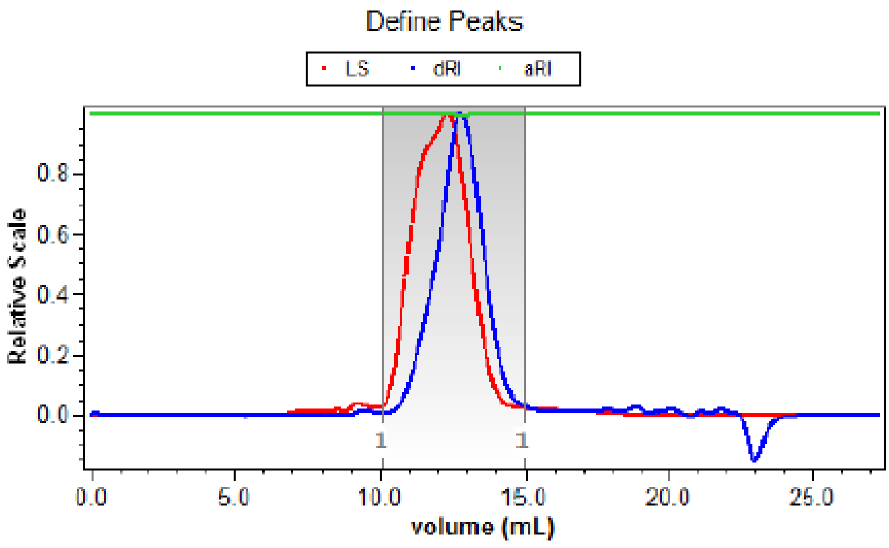

Peak Results

	Peak 1
**Masses**	
Injected Mass (µg)	540.00
Calculated Mass (µg)	534.30
Mass Recovery (%)	98.9
Mass Fraction (%)	100.0
**Molar mass moments (g/mol)**	
Mn	1.119×10^{5} (±13.554%)
Mp	1.200×10^{5} (±13.505%)
Mv	n/a
Mw	1.831×10^{5} (±13.959%)
Mz	3.738×10^{5} (±32.213%)
**Polydispersity**	
Mw/Mn	1.637 (±19.457%)
Mz/Mn	3.342 (±34.948%)
**rms radius moments (nm)**	
Rn	n/a
Rw	n/a
Rz	n/a

Figure S59. GPC chromatogram and data of polymer **P1** (t=21h)

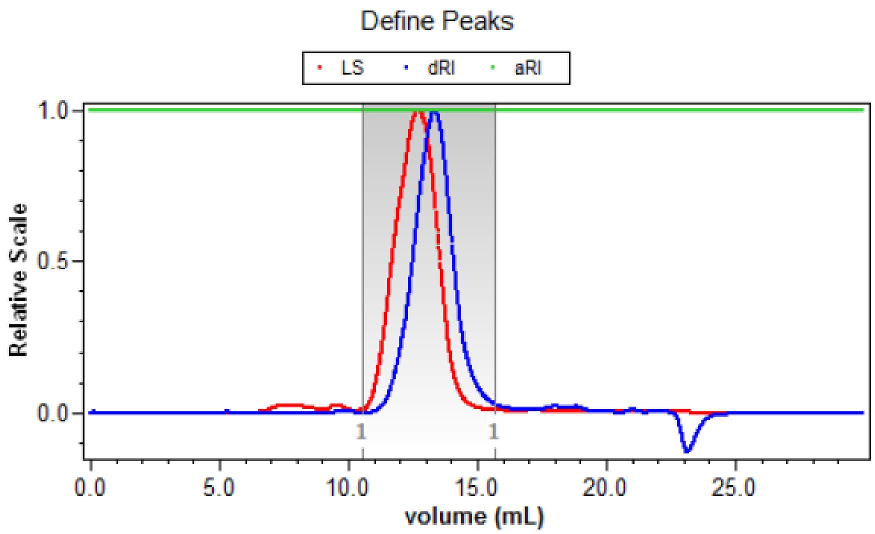

Peak Results

	Peak 1
**Masses**	
Injected Mass (µg)	430.00
Calculated Mass (µg)	424.21
Mass Recovery (%)	98.7
Mass Fraction (%)	100.0
**Molar mass moments (g/mol)**	
Mn	7.942×10^{4} (±13.607%)
Mp	1.036×10^{5} (±13.597%)
Mv	n/a
Mw	1.631×10^{5} (±13.810%)
Mz	3.897×10^{5} (±31.368%)
**Polydispersity**	
Mw/Mn	2.054 (±19.387%)
Mz/Mn	4.906 (±34.192%)
**rms radius moments (nm)**	
Rn	12.6 (±362.4%)
Rw	13.8 (±304.8%)
Rz	17.5 (±193.1%)

Figure S60. GPC chromatogram and data of polymer **P1** (t=5min)

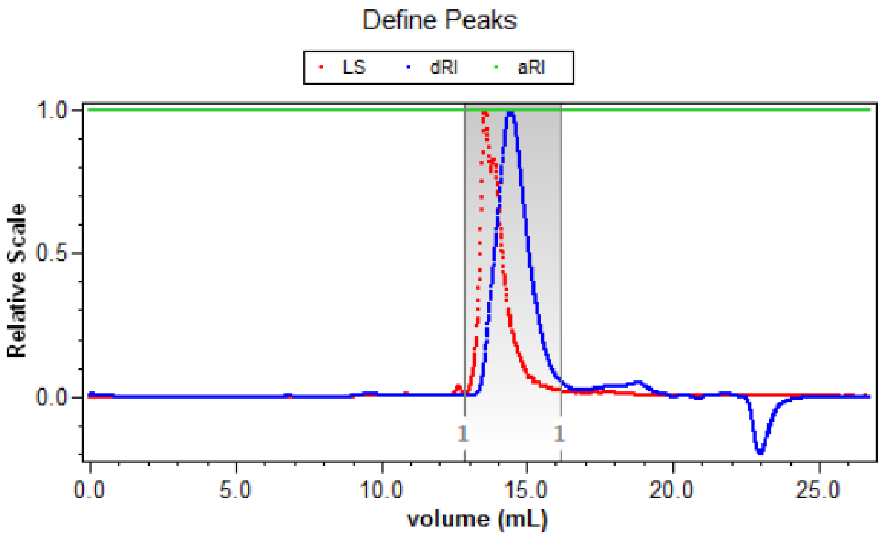

Peak Results

	Peak 1
**Masses**	
Injected Mass (µg)	420.00
Calculated Mass (µg)	290.80
Mass Recovery (%)	69.2
Mass Fraction (%)	100.0
**Molar mass moments (g/mol)**	
Mn	4.284×10^{4} (±27.951%)
Mp	5.843×10^{4} (±28.621%)
Mv	n/a
Mw	1.322×10^{5} (±47.710%)
Mz	4.738×10^{6} (±137.253%)
**Polydispersity**	
Mw/Mn	3.087 (±55.294%)
Mz/Mn	110.608 (±140.070%)
**rms radius moments (nm)**	
Rn	n/a
Rw	61.2 (±28.7%)
Rz	177.0 (±7.5%)

Figure S61. GPC chromatogram and data of polymer **P3**

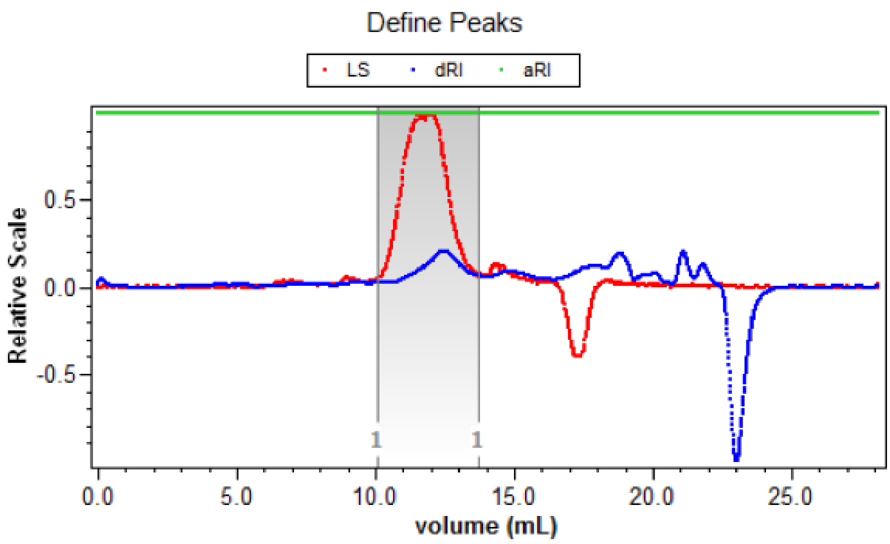

Peak Results

	Peak 1
**Masses**	
Injected Mass (µg)	200.00
Calculated Mass (µg)	12.17
Mass Recovery (%)	6.1
Mass Fraction (%)	100.0
**Molar mass moments (g/mol)**	
Mn	1.011×10^5 (±16.863%)
Mp	1.124×10^5 (±16.642%)
Mv	n/a
Mw	1.697×10^5 (±16.797%)
Mz	2.620×10^5 (±37.611%)
**Polydispersity**	
Mw/Mn	1.679 (±23.802%)
Mz/Mn	2.591 (±41.219%)
**rms radius moments (nm)**	
Rn	n/a
Rw	n/a
Rz	6.8 (±1226.9%)

Figure S62. GPC chromatogram and data of polymer **P5**

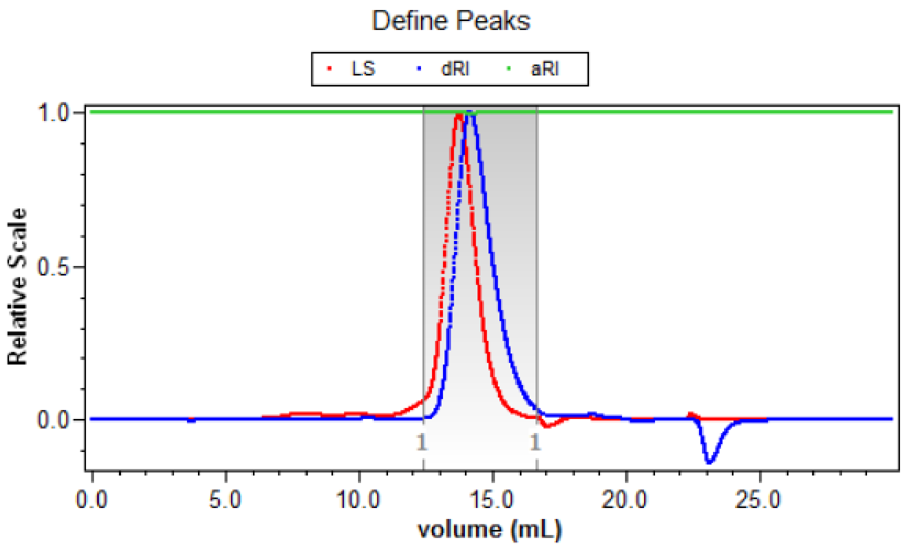

Peak Results

	Peak 1
**Masses**	
Injected Mass (µg)	450.00
Calculated Mass (µg)	439.35
Mass Recovery (%)	97.6
Mass Fraction (%)	100.0
**Molar mass moments (g/mol)**	
Mn	2.331×10^{4} (±12.076%)
Mp	4.359×10^{4} (±12.231%)
Mv	n/a
Mw	4.913×10^{4} (±12.233%)
Mz	1.065×10^{5} (±27.418%)
**Polydispersity**	
Mw/Mn	2.108 (±17.190%)
Mz/Mn	4.567 (±29.960%)
**rms radius moments (nm)**	
Rn	n/a
Rw	15.3 (±224.6%)
Rz	23.5 (±98.2%)

Figure S63. GPC chromatogram and data of polymer **P6**

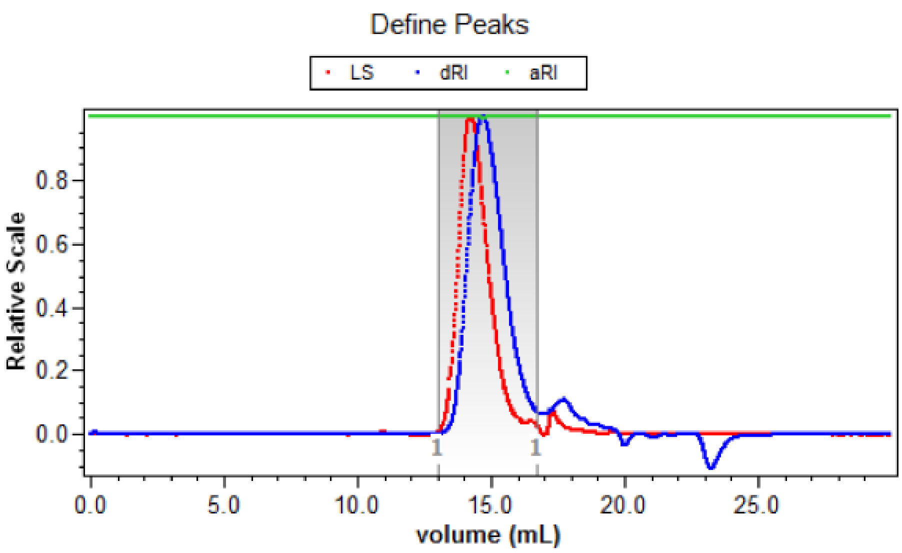

Peak Results

	Peak 1
**Masses**	
Injected Mass (µg)	360.00
Calculated Mass (µg)	329.01
Mass Recovery (%)	91.4
Mass Fraction (%)	100.0
**Molar mass moments (g/mol)**	
Mn	1.537×10^4 (±11.399%)
Mp	2.478×10^4 (±11.938%)
Mv	n/a
Mw	2.816×10^4 (±11.902%)
Mz	5.372×10^4 (±26.750%)
**Polydispersity**	
Mw/Mn	1.833 (±16.480%)
Mz/Mn	3.496 (±29.077%)
**rms radius moments (nm)**	
Rn	n/a
Rw	n/a
Rz	n/a

Figure S64. GPC chromatogram and data of polymer **P7**

*Note for calculation mass parameters

As polynorbornene standards are not commercially available, both mass parameters (M_n and M_w) of the target polymers should be reported in relation to poly(styrene) standards, with the use of an appropriate correction factor. With regard to this specific type of polymer, different values of correction factor can be found in the literature, depending on the eluent used in GPC measurement. These values range from 0.5 (CH_2Cl_2) to 2.2 (toluene) [84-87]. Furthermore, Bielawski et al., suggest that when THF is used as the eluent, the correction factor is unnecessary [85].

TGA analysis

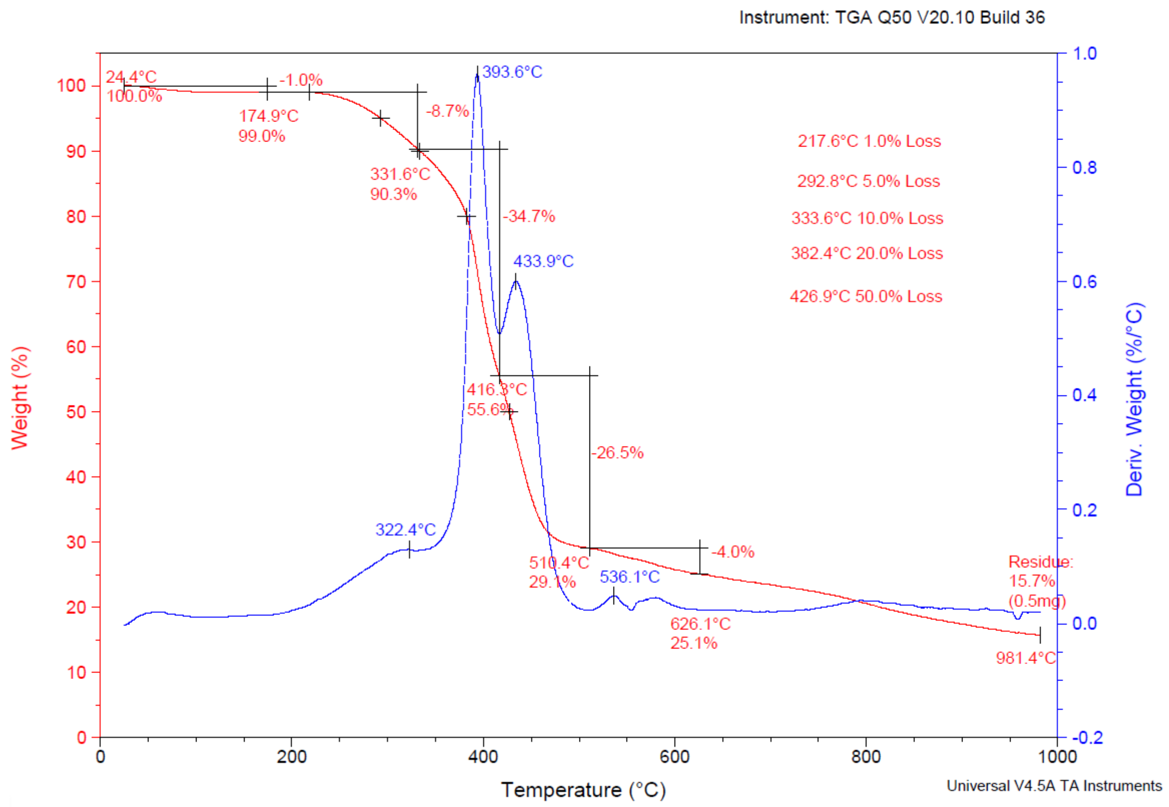

Figure S65. TGA analysis of polymer **P1**

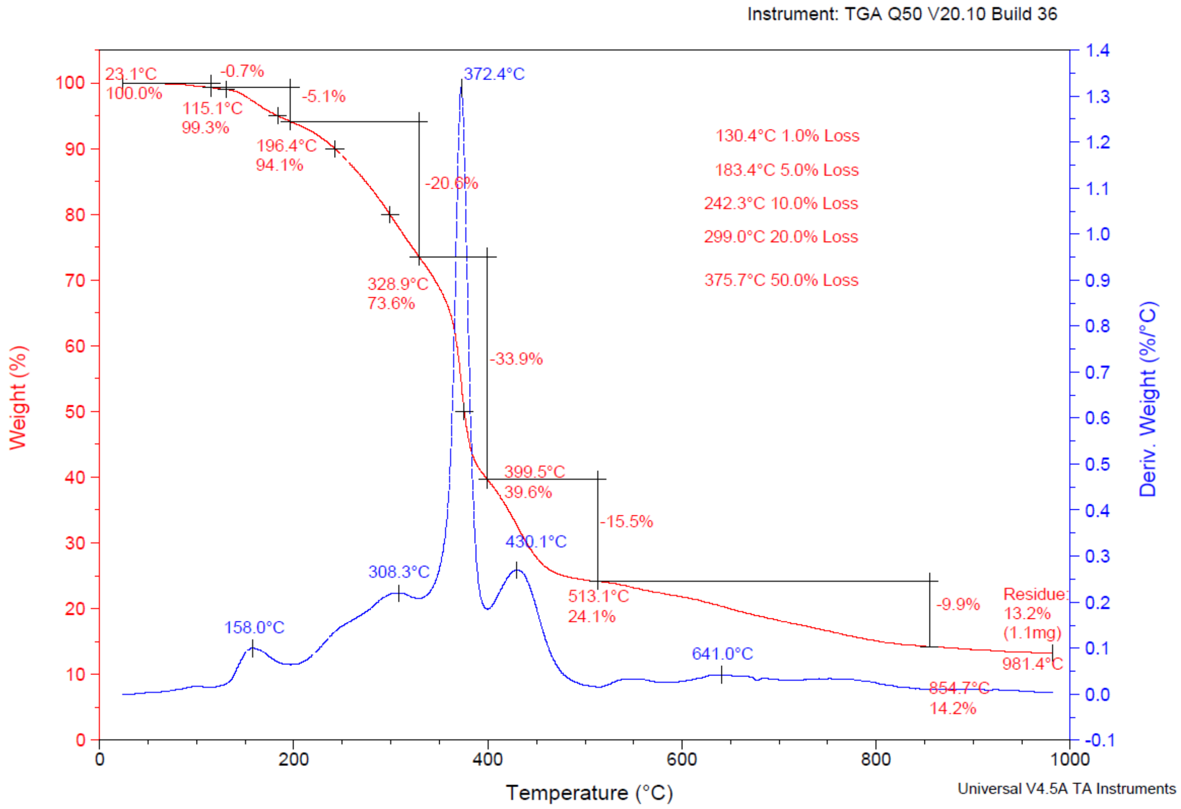

Figure S66. TGA analysis of polymer **P2**

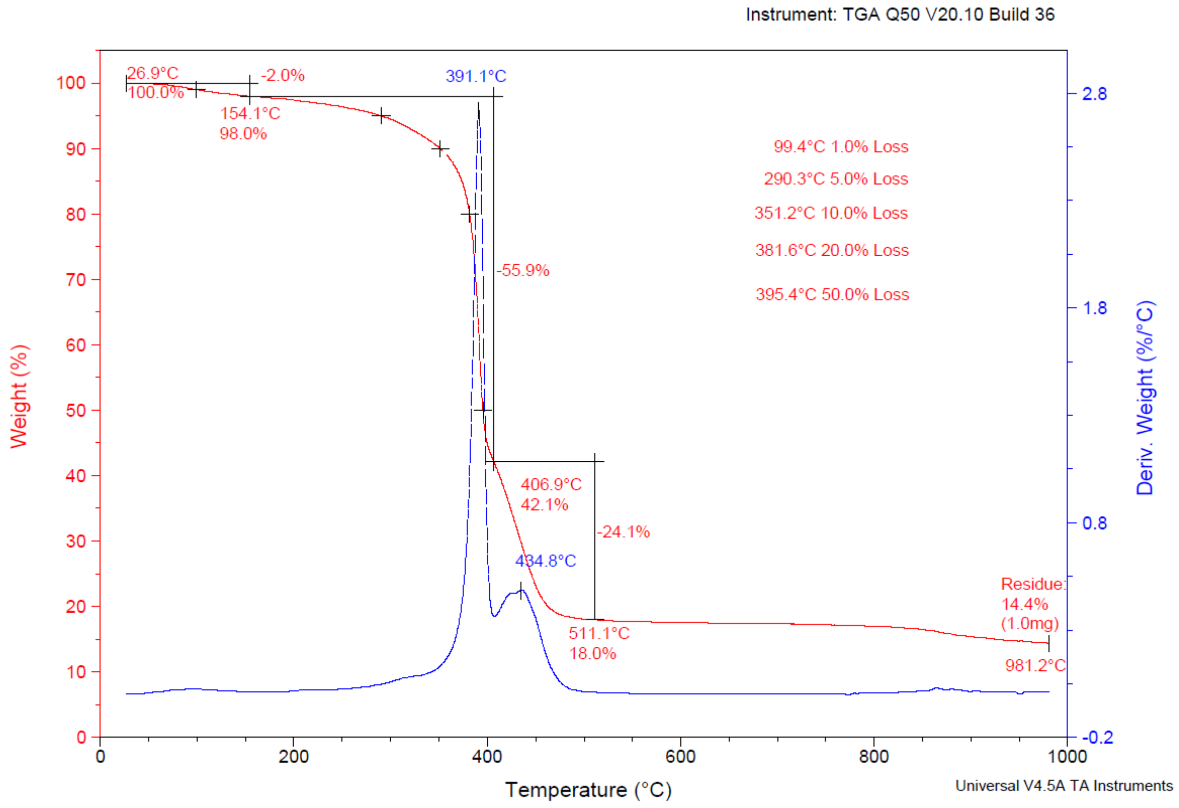

Figure S67. TGA analysis of polymer **P3**

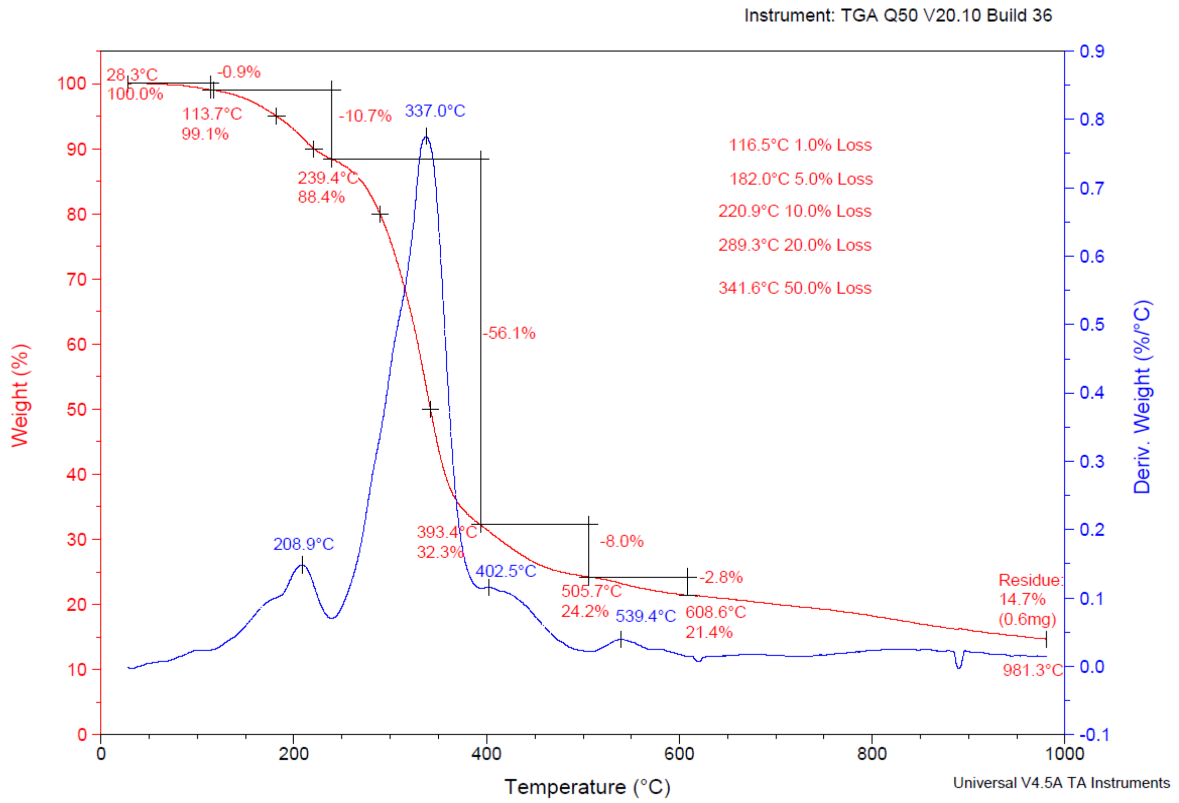

Figure S68. TGA analysis of polymer **P4**

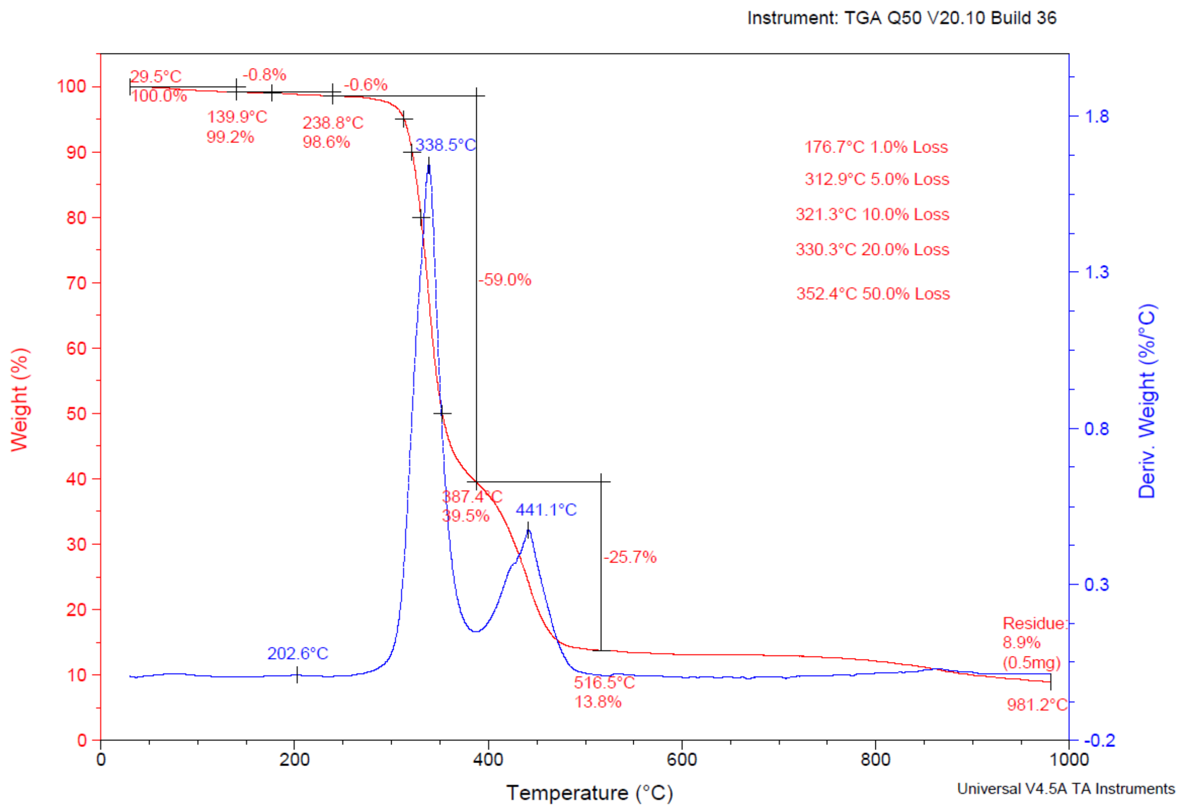

Figure S69. TGA analysis of polymer **P5**

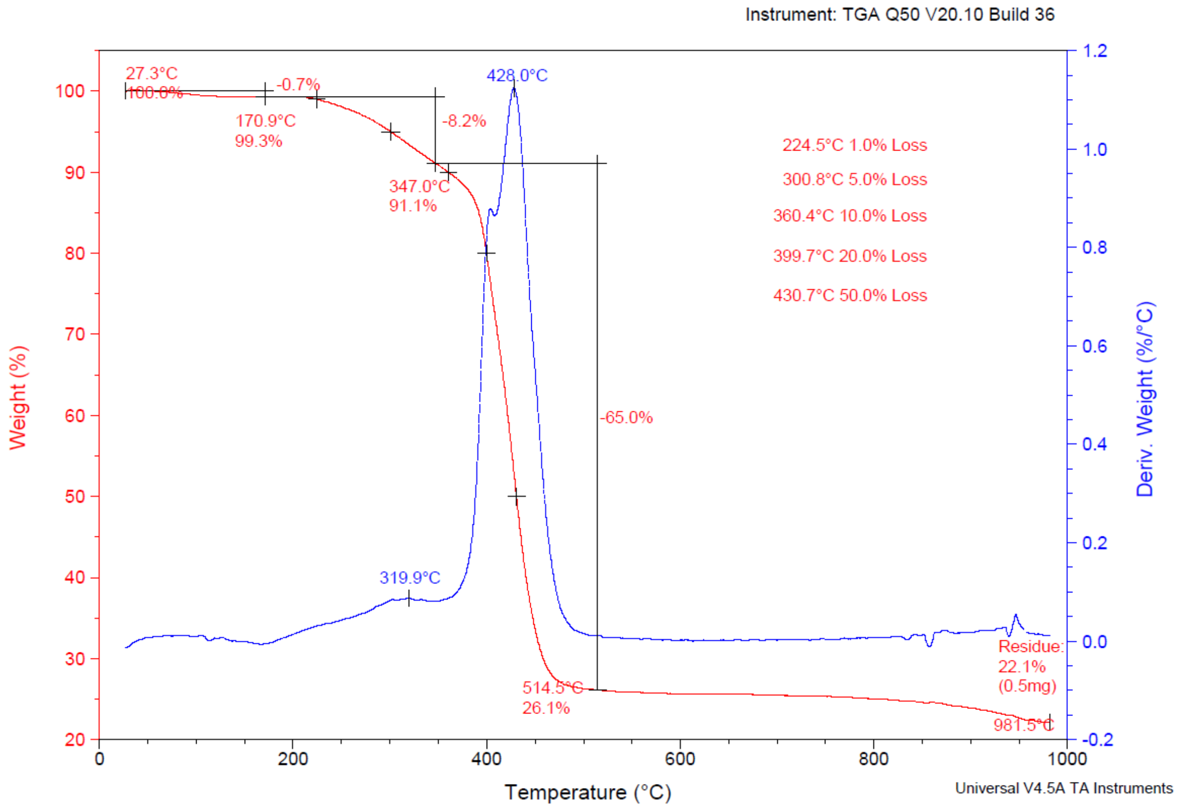

Figure S70. TGA analysis of polymer **P6**

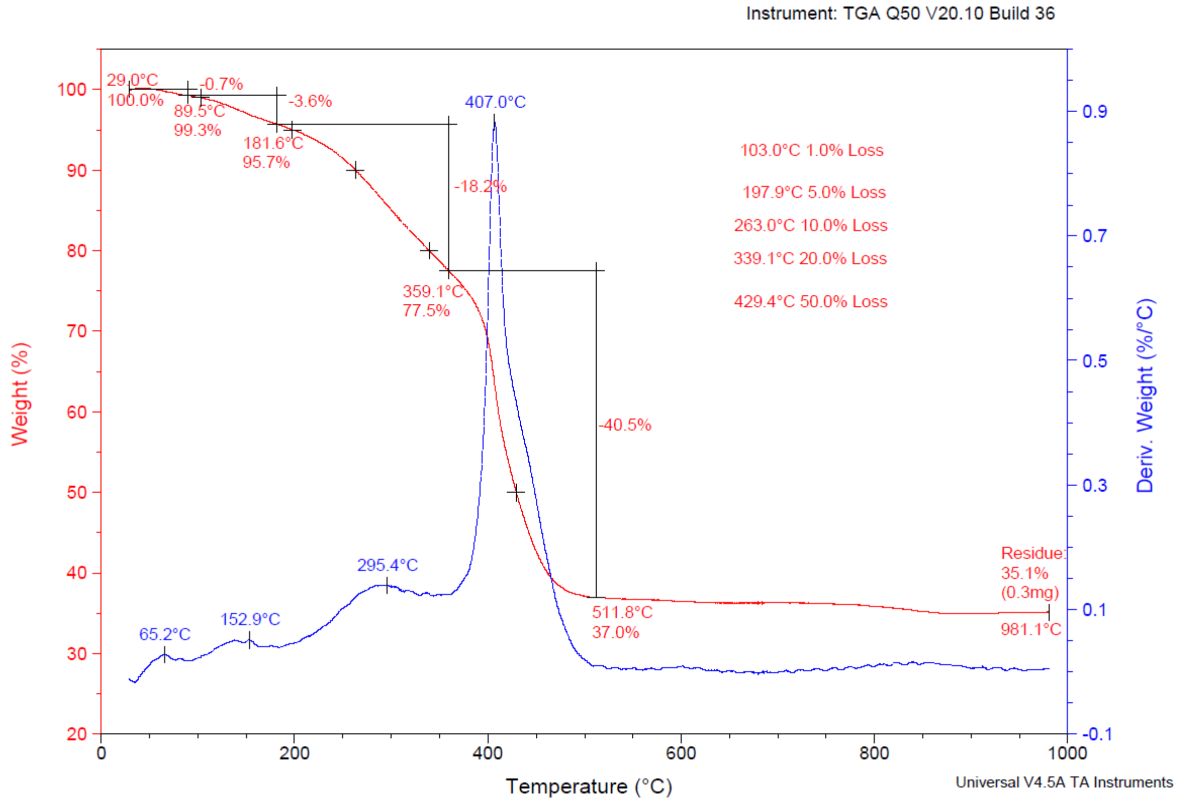

Figure S71. TGA analysis of polymer **P7**

X-Ray data

Table S1. Relevant geometrical parameters (Å,°) with s.u.'s in parentheses (different colours differentiate different types of conformation)

Bond	M1A	M1B	M2	M3A	M3B	M4A	M4B	M5	M6	M7	C2A	C2B
**B-O**	1.362(4)<br>1.353(4)	1.363(3)<br>1.346(4))	1.3583(17)<br>1.3592(17)	1.363(3)<br>1.364(3)	1.362(3)<br>1.365(3)	1.344(4)<br>1.350(4)	1.346(4)<br>1.354(4)	1.351(4)<br>1.351(4)	1.3666(18)<br>1.3682(18)	1.3633(17)<br>1.3634(17)	1.445(3)<br>1.451(3)	1.449(3)<br>1.456(3)
**B-C**	1.548(5)	1.556(4)	1.5840(18)	1.582(3)	1.566(3)	1.587(4)	1.576(4)	1.569(7)	1.563(2)	1.5660(19)	1.623(4)	1.619(4)
**B-N**											1.660(3)	1.652(3)
**C11-C12**	1.306(4)	1.281(5)	1.326(2)	1.324(4)	1.323(4)	1.308(6)	1.315(5)	1.317(8)	1.329(2)	1.3384(18)	1.315(5)	1.310(5)
**O-B-O**	125.4(4)	126.6(3)	126.84(12)	126.1(2)	125.8(2)	127.4(3)	126.6(3)	127.2(5)	126.66(14)	126.76(12)	117.1(2)	116.58(19)
**O-B-C**	117.8(3)<br>116.8(3)	116.7(3)<br>116.7(3)	116.92(11)<br>116.23(11)	120.4(2)<br>113.5(2)	119.9(2)<br>114.3(2)	117.5(3)<br>115.0(3)	116.3(3)<br>117.1(3)	116.4(3)<br>116.4(3)	115.45(12)<br>117.86(13)	116.68(11)<br>116.53(12)	109.5(2)<br>110.3(2)	109.51(19)<br>110.2(2)
**O-B-C-C**	-4.4(4)<br>175.5(3)<br>179.5(3)<br>-0.6(4)	-5.5(4)<br>175.2(2)<br>174.5(2)<br>-4.8(4)	60.38(16)<br>-118.92(13)<br>-120.33(13)<br>60.37(16)	151.6(2)<br>-29.7(3)<br>-33.0(3)<br>145.7(2)	158.9(2)<br>-20.2(3)<br>-20.5(4)<br>160.4(2)	115.0(3)<br>-66.1(4)<br>-69.0(4)<br>110.0(3)	101.7(4)<br>-77.7(4)<br>78.5(4)<br>-102.2(4)	176.8(4)<br>-1.0(6)<br>1.0(6)<br>-176.8(4)	-179.90(13)<br>-1.8(2)	-177.70(12)<br>4.23(19)	-29.4(3)<br>-159.8(2)<br>153.8(2)<br>23.5(3)	160.2(2)<br>30.7(3)<br>-22.7(3)<br>-152.2(2)
**A/B**	61.4	32.3	61.4	32.3	20.3	68.1	77.6	3.6	2.1	9.3	3.7 (ph)<br>82.7 (pyr)<br>0.524A	5.4 (ph)<br>83.4 (pyr)<br>0.525A

Table S2. Crystal data, data collection and structure refinement.

Compound	**01_M1**	**02_M2**	**03_M3**	**04_M4**
Formula	$C_{15}H_{17}BO_2$	$C_{25}H_{21}BO_2$	$C_{23}H_{21}BO_2$	$C_{23}H_{20}BBrO_2$
Formula weight	240.09	364.23	340.21	419/11
Crystal system	triclinic	monoclinic	monoclinic	triclinic
Space group	P-1	$P2_1/n$	$P2_1/n$	P-1
a(Å)	6.2301(10)	7.5384(2)	18.0305(5)	10.1316(4)
b(Å)	12.5291(13)	19.2866(6)	11.3247(3)	13.7811(6)
c(Å)	16.9572(17)	12.3729(4)	18.1014(6)	14.1252(4)
α(°)	91.819(8)	90	90	93.915(3)
β(°)	95.290(11)	93.618(3)	113.987(4)	98.154(3)
γ(°)	98.447(11)	90	90	99.809(3)
$V(Å^3)$	1302.3(3)	1795.31(9)	1588.1(3)	1915.09(13)
Z	4	4	8	4
D_x(g cm^{-3})	1.225	1.348	1.338	1.454
F(000)	512	768	1440	856
μ(mm^{-1})	0.078	0.649	0.647	2.161
Reflections:				
collected	12224	7184	27336	22973
unique (R_{int})	4603 (0.0587)	3538 (0.0119)	25122 (0.0286)	7761 (0.0191)
with I>2σ(I)	2357	3335	23599	5137
R(F) [I>2σ(I)]	0.0678	0.0401	0.0557	0.0461
wR(F^2) [I>2σ(I)]	0.1578	0.1032	0.1421	0.0977
R(F) [all data]	0.1425	0.0424	0.0587	0.0799
wR(F^2) [all data]	0.1961	0.1047	0.1456	0.1124
Goodness of fit	0.965	1.050	1.044	1.016
max/min Δρ (e·Å^{-3})	0.26/-0.24	0.20/-0.16	0.62/-0.22	0.75/-0.67
**CCDC deposition number**	**2283412**	**2283411**	**2283408**	**2283406**

Table S3. Crystal data, data collection and structure refinement.

Compound	**05_M5**	**06_M6**	**07_M7**	**08_C2**
Formula	$C_{15}H_{15}BBr_2O_2$	$C_{18}H_{21}BO_2$	$C_{23}H_{23}BO_2$	$2(C_{22}H_{27}BN_2O_2)\cdot H_2O$
Formula weight	397.90	280.16	342.22	742.54
Crystal system	orthorhombic	monoclinic	monoclinic	triclinic
Space group	$Cmc2_1$	$P2_1/n$	$P2_1/c$	P-1
a(Å)	11.6581(3)	12.1503(5)	10.4466(4)	11.9779(5)
b(Å)	21.1145(7)	5.9690(2)	19.5704(7)	12.5260(5)
c(Å)	6.11316(16)	20.7756(8)	8.8160(4)	16.3292(6)
α(°)	90	90	90	76.655(2)
β(°)	90	100.994(4)	96.742(4)	89.340(2)
γ(°)	90	90	90	82.310(2)
V(Å^3)	1504.78(7)	1479.10(10)	1789.92(12)	2361.89(16)
Z	4	4	4	2
D_x(g cm^{-3})	1.756	1.258	1.270	1.044
F(000)	784	600	728	796
μ(mm^{-1})	5.384	0.079	0.078	0.532
Reflections:				
collected	14200	12843	18531	40455
unique (R_{int})	1556 (0.0319)	3341 (0.0212)	4099 (0.0199)	9197 (0.0641)
with I>2σ(I)	2649	2727	23599	7230
R(F) [I>2σ(I)]	0.0246	0.0422	0.0423	0.0722
wR(F^2) [I>2σ(I)]	0.0570	0.1003	0.0975	0.1758
R(F) [all data]	0.0338	0.0547	0.0509	0.0893
wR(F^2) [all data]	0.0603	0.1080	0.1017	0.1905
Goodness of fit	1.067	1.036	1.038	1.042
max/min Δρ (e·Å^{-3})	0.25/-0.30	0.29/-0.28	0.28/-0.21	0.38/-0.47
**CCDC deposition number**	**2283407**	**2283410**	**2283409**	**2388368**
